# Supplementary material for: The protocol for a cluster randomized controlled trial to evaluate couple-based violence prevention education and its ability to reduce intimate partner violence during pregnancy in Southwest Ethiopia
Source: PLoS One. 2024 May 13;19(5):e0303009. doi: 10.1371/journal.pone.0303009 (PMC11090299; doi:10.1371/journal.pone.0303009)
Supplement: S1 Fig — (DOC) [file pone.0303009.s001.doc]

Figure 1. Recommended content for the schedule of enrolment, interventions, and assessments.*

|  |  | **STUDY PERIOD** | | | | | | | |
| --- | --- | --- | --- | --- | --- | --- | --- | --- | --- |
|  | **Enrolment** | **Allocation** | **Post-allocation** | | | | | | **Close-out** |
| **TIMEPOINT**** | ***-Month_1_*** | **0** | ***Month_1_*** | ***Month_2_*** | ***Month_3_*** | ***Month _4_*** | ***Month5*** | ***Month6*** | ***Month_8_*** |
| **ENROLMENT:** |  |  |  |  |  |  |  |  |  |
| **Eligibility screen** | X |  |  |  |  |  |  |  |  |
| **Informed consent** | X |  |  |  |  |  |  |  |  |
| **Allocation** |  | X |  |  |  |  |  |  |  |
| **Baseline assessment** |  | X |  |  |  |  |  |  |  |
| **INTERVENTIONS:** |  |  |  |  |  |  |  |  |  |
| ***[Couple based violence prevention education]*** |  |  |  |  |  |  |  |  |  |
| ***[Usual Standard of care]*** |  |  |  |  |  |  |  |  |  |
| **ASSESSMENTS:** |  |  |  |  |  |  |  |  |  |
| ***[Background/ demographic ,IPV in the last 12 months and recent pregnancy]*** |  |  |  |  |  |  |  |  | X |
| ***[Knowledge, attitude, controlling behavior toward IPV]*** |  |  |  |  |  |  |  |  | X |
| ***[Autonomy and self-efficacy]*** |  |  |  |  |  |  |  |  | X |

*Recommended content can be displayed using various schematic formats. See SPIRIT 2013 Explanation and Elaboration for examples from protocols.

**List specific timepoints in this row.
