# Supplementary material for: The protocol for a cluster randomized controlled trial to evaluate couple-based violence prevention education and its ability to reduce intimate partner violence during pregnancy in Southwest Ethiopia
Source: PLoS One. 2024 May 13;19(5):e0303009. doi: 10.1371/journal.pone.0303009 (PMC11090299; doi:10.1371/journal.pone.0303009)
Supplement: S2 Table — (DOCX) [file pone.0303009.s003.docx]

S2 Table. Items from the WHO Trial Registration Data Set

| **Category** | **Information** |  |  |
| --- | --- | --- | --- |
| **Primary Registry and Trial Identifying Number** | ClinicalTrials.gov CT05856214 |  |  |
| **Date of Registration in Primary Registry** | May 4, 2023 |  |  |
| **Secondary Identifying Numbers** | None |  |  |
| **Source(s) of Monetary or Material Support** | Jimma and Wachemo University |  |  |
| **Primary Sponsor** | Jimma University |  |  |
| **Secondary Sponsor(s)** | Wachemo,University |  |  |
| **Contact for Public Queries** | BSc, MPH/RH [zeledutamo@gmail.com] |  |  |
| **Contact for Scientific Queries** | BSc, MPH/RH, Jimma University, Ethiopia |  |  |
| **Public Title** | Couple based violence prevention education to reduce intimate partner violence for improving maternal health |  |  |
| **Scientific Title** | Effect of Couple Based Violence Prevention Education on Intimate Partner Violence During Pregnancy in Southwest, Ethiopia: study protocol for a cluster randomized controlled trial |  |  |
| **Countries of Recruitment** | Ethiopia |  |  |
| **Health Condition(s) or Problem(s) Studied** | Intimate partner violence during pregnancy |  |  |
| **Intervention(s)** | Couple-Based Violence Prevention Education(CBVPE) |  |  |
| **Key Inclusion and Exclusion Criteria** | The inclusion criteria during pregnancy will be pregnant women in the first trimester with their husbands , couple whose wife has at least one live birth, couple who lived in the study area at least six months before the study, couple whose wife’s inter-pregnancy interval is less than two years, and couple willing to be visited at home  Exclusion Criteria will be couples with: a severe medical illness, hearing or communication problems, and a plan to move out from the intervention and control clusters in the next 8 months |  |  |
| **Study Type** | Interventional |  |  |
|  | Method of allocation – random, Masking is used - data collectors are blinded, Assignment – parallel |  |  |
|  | Purpose – Behavioural promotion |  |  |
| **Date of First Enrolment** | July 2023 |  |  |
| **Target Sample Size** | 432couples (432 wives + 432 husbands)=864 participants |  |  |
| **Recruitment Status** | Recruitment completed |  |  |
| **Primary Outcome(s)** | The magnitude of intimate partner violence during pregnancy among women |  |  |
| **Key Secondary Outcomes:** | couples' knowledge, attitudes, and husbands’ controlling behavior (AC) toward IPV, women’s autonomy and self-efficacy |  |  |
|  |  |  |  |
|  | | |  |
|  | | |  |
|  | | |  |
|  | | |  |
|  | | |  |
|  | | |  |
|  | | |  |
