# Supplementary material for: The protocol for a cluster randomized controlled trial to evaluate couple-based violence prevention education and its ability to reduce intimate partner violence during pregnancy in Southwest Ethiopia
Source: PLoS One. 2024 May 13;19(5):e0303009. doi: 10.1371/journal.pone.0303009 (PMC11090299; doi:10.1371/journal.pone.0303009)
Supplement: S1 File — (PDF) [file pone.0303009.s004.pdf]

Jimma University Institute of Health

Institutional Review Board

Ref.No- **JUIH/IRB/** 222/22

Date: 08/11/2022

To: Mr. Zeleke Dutamo

Subject: Ethical Approval of Research Protocol

The IRB of Institute of Health has reviewed your research project '**EFFECT OF COUPLE BASED VIOLENCE PREVENTION EDUCATION ON INTIMATE PARTNER VIOLENCE DURING PREGNANCY IN HADIYA ZONE, SOUTH WEST ETHIOPIA: A CLUSTER RANDOMIZED CONTROLLED TRIAL''**

Thus, this is to notify that this research protocol has presented to the IRB meets the ethical and scientific standards outlined in national and international guidelines. Hence, we are pleased to inform you that your research protocol is ethically cleared under the following strict conditions:

1. Any significant deviation from the methodological details indicated in the approved protocol must be communicated to the IRB before it has been implemented.
2. Approval shall be only for a period of twelve months. The principal investigator is required to submit an application for the renewal of the ethical approval.
3. The Committee must be notified, in writing, of any alteration to the project including unforeseen events/circumstances that might affect the acceptability of the approved protocol.
4. The Principal researcher is required to immediately notify the committee in the event of any adverse effects on participants or of any unforeseen events that might affect continued ethical acceptability or amendment to the original consent form.
5. The inability of the Principal Researcher to continue in that role or any other change in research personnel involved in the project should be notified to the committee immediately.

The IRB wishes you every success in your research.

Mr.Gizachew Tilahun
Assistant Professor
Chair,JUIH-IRB

E-mail: ethicsjuirb@gmail.com

ጅማ ዩኒቨርሲቲ ጤና ኢንስቲትዩት
Jimma University Institutional Review board
Jimma University Health Institute

Tel. +251-47 11 114 57
PBX: +251471111458-60-
Fax: +2514711114 50
+251471112040
P.O.Box. 378
JIMMA, ETHIOPIA
e-mail: ero@ju.edu.et
website: http://www.ju.edu.et
