# Supplementary material for: The protocol for a cluster randomized controlled trial to evaluate couple-based violence prevention education and its ability to reduce intimate partner violence during pregnancy in Southwest Ethiopia
Source: PLoS One. 2024 May 13;19(5):e0303009. doi: 10.1371/journal.pone.0303009 (PMC11090299; doi:10.1371/journal.pone.0303009)
Supplement: S2 File — (DOCX) [file pone.0303009.s005.docx]

JIMMA UNIVERSITY

INSTITUTE OF HEALTH

GRADUATE STUDIES

EFFECT OF COUPLE BASED VIOLENCE PREVENTION EDUCATION ON INTIMATE PARTNER VIOLENCE DURING PREGNANCY IN HADIYA ZONE, SOUTH WEST ETHIOPIA: A CLUSTER RANDOMIZED CONTROLLED TRIAL

BY:-ZELEKE DUTAMO AGDE (BSC, MPH/RH)

A PhD RESEARCH PROPOSAL SUBMITTED TO THE SCHOOL OF GRADUATE STUDIES OF JIMMA UNIVERSITY FOR THE FULFILLMENT OF THE REQUIREMENTS FOR THE DEGREE OF DOCTOR OF PHILOSOPHY (PhD) IN PUBLIC HEALTH (REPRODUCTIVE HEALTH)

JULY, 2022

JIMMA UNIVERSITY

JIMMA UNIVERSITY

INSTITUTE OF HEALTH

GRADUATE STUDIES

THE EFFECT OF COUPLE BASED VIOLENCE PREVENTION EDUCATION ON INTIMATE PARTNER VIOLENCE DURING PREGNANCY IN HADIYA ZONE, SOUTH WEST ETHIOPIA: A CLUSTER RANDOMIZED CONTROLLED TRIAL

BY: ZELEKE DUTAMO AGDE (BSc, MPH/RH)

SUPERVISORS

1. MULUEMEBET ABERA (PhD, ASSOCIATE PROFESSOR)

JIMMA UNIVERSITY, ETHIOPIA

1. NEGA ASSEFA(PhD, PROFESSOR)

HARAMAYA UNIVERSITY, ETHIOPIA

1. JEANNETTE H.MAGNUS(PhD,PROFESSOR)

JULY, 2022

JIMMA UNIVERSITY

#

# DECLARATION FORM

Letter for Declaration (Dissertation proposal work)

I, the under signed, declared that this is my bona fide original work, has never been presented in this or any other University, and that all the resources and materials used for the thesis, have been fully acknowledged.

Name: *Zeleke Dutamo Agde*

Signature: _________________________________________

Date: __________________________________________

Place: Jimma University, Jimma Ethiopia

Date of submission: _____________________________________

This dissertation proposal has been submitted for examination with my approval as Candidate’s Promoter (supervisor).

Name: *Muluemebet Abera*  Signature: ____________ Date: _________

Co-promoter (Co supervisor)

Name: *Nega Assefa*  Signature: ____________ Date: ___________

Co-promoter (Co supervisor)

Name: *Jeannette H.Magnus*  Signature: ____________ Date: ___________

Team Leader, Dep’t of Population & Family Health Director, Research Office, IOH, JU

Name: ____________________ Name: _________________

Signature: ________________ Signature: ________________

Date: ____________________ Date: _________________

# ACKNOWLEDGEMENT

I would like to thank my PhD supervisors, Muluemebet Abera (PhD, Associate professor), Nega Assefa (PhD, Professor) and Jeannette H.Magnus (PhD, professor) for the countinous guidance and support throuout this PhD thesis proposal development. I have great appreciation for all my supervisors who are helpful in giving me feedbacks as soon as I need support.

I want to express my deepest thanks to both Jimma and Wachemo University for giming me this PhD chance which will help to develop my future career.

# ABBREVIATIONS

ANC Antenatal Care

AOR Adjusted Odds Ratio

FGD Focus Group Discussion

GBV Gender Based Violence

GEE Generalized Estimating Equations

HEP Health Extension Program

HEWs Health Extension Workers

HSDP Health Sector Development Plan

IPVp Intimate Partner violence in pregnancy

KII Key Informant Interview

OR Odds Ratio

SDGs Sustainable Development Goals

SNNPR Southern Nations, Nationalities and Peoples Region

UN United Nations

VAW Violence against Women

WHO World Health Organization

**Table of Contents**

[6.16.1 Couple education contents and sessions 33](#_Toc148587523)

[6.16.2 Description of education sessions 35](#_Toc148587524)

[6.16.3 Appointment and follow-up 35](#_Toc148587525)

[6.17 questionnaire translation, validity and reliability 27](#_Toc148587526)

[6.18 outcome of interest and measurement 28](#_Toc148587527)

[6.18 Data collectors 31](#_Toc148587528)

[6.19 Training 32](#_Toc148587530)

[6.21 Research team 33](#_Toc148587531)

[6.22 Data collection instruments 33](#_Toc148587533)

[**6.20 Data Quality control** 34](#_Toc148587534)

[7. Work plan 38](#_Toc148587535)

[8. Budget 41](#_Toc148587536)

[8.1 Budget Breakdown 41](#_Toc148587537)

[8.2 Budget summary 42](#_Toc148587538)

[9. REFERENCES 43](#_Toc148587539)

# List of tables

**Tables Pages**

[Table 1: pregnancy screening questions 35](#_Toc148612167)

[Table 2:summary of the intervention package and implementation process 27](#_Toc148612168)

[Table 3:Participants’ timeline of enrolment, implementation and assessment schedule 36](#_Toc148612169)

[Table 4: work plan for effect of couples based violence prevention education on intimate partner violence during pregnancy in Hadiya zone, SNNPR from 2022-2023 39](#_Toc148612170)

[Table 5: Budget breakdown for couple based violence prevention education in Hadiya zone, 2022/2 41](#_Toc148612171)

[Table 6: budget summary for couple based violence prevention education in Hadiya zone, 2022-20223 42](#_Toc148612172)

# List of Figures

Figures Pages

[Figure 1: Outcomes of IPV during pregnancy, Source: World Health Organization (WHO) Information Sheet (2011). 2](#_Toc148612002)

[Figure 2 A Conceptual frame work adapted from Glanz K. et al., 2008. 15](#_Toc148612003)

[Figure 3: diagrammatic representation of sampling procedure 34](#_Toc148612005)

[Figure 4: diagrammatic representation of study activities 37](#_Toc148612006)

[Figure 5: Consort flow diagram 40](#_Toc148612007)

[Figure 6: the schedule for couples education and message contents of intervention group 42](#_Toc148612008)

[Figure 7:Schematic representation of validation process of the questionnaire (IPVp) 28](#_Toc148612009)

# Summary

**Background:** **Background**: A significant proportion of women in Ethiopia suffer from violence by their intimate partner during pregnancy, which has adverse maternal and newborn outcomes. Couple-focused interventions are effective in reducing and/or controlling violence between women and their intimate partners. However, interventions addressing intimate partners of the victims are not well studied, particularly in the Ethiopian setting.

**Objective**: The aim of this study is to evaluate the effect of couple-based violence prevention education on IPV during pregnancy in Hadiya Zone, Southwest Ethiopia.

**Methods and Materials**: the study will be conducted in Hadiya zone, Central Ethiopia. It is located 235 km southwest from the capital, Addis Ababa. The study will be conducted in four districts of Hadiya zone namely: Soro, Lemo, Ana Lemo and Duna districts. The study will take 8 months (6 months intervention+2 months post intervention). We will use a cluster randomized controlled trial to evaluate the effectiveness of couple-based violence prevention education (CBVPE) compared to routine care in reducing intimate partner violence during pregnancy. Sixteen kebeles will be randomly assigned to 8 interventions and 8 control groups. In the trial, 426 couples whose wives are in the first trimester will participate. Health extension workers (HEWs) will provide health education. Data will be collected at baseline and endline. All the collected data will be analyzed using SPSS or STATA version 16.0. We will use the McNemar test to appreciate the differences in outcomes of interest in both intervention and control groups before and after the intervention for categorical data. A paired t-test will be used to compare continues outcome of interest in the intervention and the control groups after and before the intervention. The GEE (Generalized Estimating Equation), will be used to test the independent effect of the intervention on the outcome of the interest. Data analysis will be performed with an intention-to-treat analysis approach. During the analysis, the effect size, confidence interval, and p-value will be calculated. All tests will be two-sided, and statistical significance will be declared at p < 0.05.

**Work plan and budget**: the project will be accomplished within 24 months. One month for participant recruitment and one month for baseline data collection; this starts in June 2022. The intervention will be implemented in 6 months, and end-line data collection will be carried out after two months of the intervention. To accomplish this project, about 276,880 birr (two hundred seventy-six thousand and eight hundred and eighty birr) will be needed for successful accomplishment.

## 1. Itroduction

## 1.1 Background to the study

The most widely spread form of gender-based violence (GBV) is intimate partner violence (WHO, 2007). Intimate partner violence (IPV) refers to “any behavior within an intimate relationship that causes physical, psychological, or sexual harm to the partner” (UN, 1994). The majority of women's abuse is committed by close partners. Intimate partner violence in pregnancy is an important public health agenda and human rights violation (WHO, 2011b). Intimate partner violence is a universal phenomenon; it occurs in all settings and among all socioeconomic, religious, and cultural groups. In 2018, the World Health Organization reported that one in three women experiences physical and/or sexual violence by an intimate partner throughout the world in their lifetime, with the highest proportion in Africa, followed by Southeast Asia (WHO, 2021).

There are different types of intimate partner violence, including physical abuse, sexual assault, coercion, social isolation, emotional abuse, economic control, and deprivation (Dichter et al., 2018). Physical violence or abuse is any act or behavior such as hitting, slapping, burning, or using weapons. Sexual violence can be in the form of rape or forceful, unwanted sexual contact, whereas emotional or psychological violence can be in the form of using words or manipulation to inflict harm. The IPV takes the lion share of violence against women (WHO, 2021).

Violence during pregnancy is a possible risk to not only the pregnant mother but also to the unborn child. Studies in different countries have found that IPV during pregnancy is associated with adverse maternal outcomes including emotional distress, depression, anxiety, insufficient or inconsistent prenatal care, inadequate gestational weight gain, higher rates of smoking, alcohol use, and mortality (Mohammed et al., 2017; WHO, 2011b). IPV in pregnancy is also associated with increased health care utilization and increased health care costs (Bonomi et al., 2009; Daoud et al., 2020; Devries et al., 2010). Violated pregnant women are at higher risk of miscarriage, abruption of placentae, preterm delivery, preeclampsia, and perinatal morbidity and mortality (Alhusen et al., 2015; Belay et al., 2019; WHO, 2021).

Violence during pregnancy is a possible risk to not only the pregnant mother but also to the unborn child. Studies in different countries have found that IPV during pregnancy is associated with adverse maternal outcomes, including emotional distress. Intimate partner violence health outcomes during pregnancy are categorized into fatal and non-fatal outcomes. The fatal outcomes include homicide and suicide, whereas non-fatal health outcomes are categorized as negative health behaviors, reproductive health, and physical or mental outcomes (WHO, 2011b). Adverse maternal and newborn health outcomes are non-fatal health consequences of intimate partner violence during pregnancy (Figure 1).

Intimate partner violence outcomes (IPVp)

Fatal outcomes

Homicide

Suicide

Death

Non-Fatal outcomes

**Negative health behavior**

Antenatal alcohol and drug abuse

Smoking during pregnancy

Delayed prenatal care

**Reproductive health**

Low- birth weight

Pre-mature-birth

Low weight gain

Obstetric complications

STIs /HIV

Miscarriage

Unsafe abortion

**Physical and mental health**

Injury

Physical impairments

Physical symptoms

Depression

Anxiety

Difficulties or lack of attachment to child

Effect on the child

Disability

Figure 1: Outcomes of IPV during pregnancy, Source: World Health Organization (WHO) Information Sheet (2011).

## Studies found that women who reported intimate partner violence were significantly associated with intrauterine retardation and pre-mature labor (Martin-de-Las-Heras et al., 2019; Yu et al., 2018). Women who experience intimate partner violence during pregnancy also found that increased women’s risk of miscarriage and abortion (Afiaz et al., 2020; Alhusen et al., 2015). Intimate partner violence during pregnancy is also associated with a higher risk of antepartum and postpartum depression, anxiety, suicidal attempts, a lack of attachment to the child, and a lower level of breast feeding (Alhusen et al., 2015; Bonomi et al., 2009; Martin-de-Las-Heras et al., 2019; WHO, 2011a). Intimate partner violence is considered a tip of the iceberg, or silent epidemic, since many victims do not reveal their experience of violence from an intimate partner due to barriers (Palermo et al., 2014). Intimate partner violence has a spill-over effect. It affects not only the women and the newborns but also the family, community, society, and the nation at large (Jill Ryan and Roman, 2019).1.2 Statement of the problem

Violence against women is a phenomenon that occurs in all countries around the world (WHO, 2011b). Violence against women has been an important public health, social policy, and human rights concern since the 1993 World Conference on Human Rights and the Declaration on the Elimination of Violence against Women (UN, 1993). The majority of violence against women is committed by an intimate partner. Each year, between 1.5 and 5.3 million women are physically and/or sexually assaulted by an intimate partner (K. M. Devries et al., 2013). In the WHO multi-country study on women’s health and domestic violence against women, it was found that the prevalence of physical intimate violence in pregnancy was one percent in Japan and 28% in Peru (WHO, 2010). According to the analysis of Demography and Health Surveys and the International Violence against Women Report, the prevalence of intimate partner violence during pregnancy is lowest (2%) in Australia, Denmark, Cambodia, and the Philippines, and highest (13.5%) in Uganda. A review of clinical studies from Africa reported in 2011 showed prevalence rates of 23–40% for physical, 3–27% for sexual, and 25–49% for emotional intimate partner violence during pregnancy (Shamu et al., 2011).

Studies conducted in sub-Saharan African and Asian countries showed that the prevalence of intimate partner violence is one of the highest in Ethiopia (74%), following Jordan (87%; Muluneh et al., 2020). In a multi-country study conducted in 10 different countries, a rate ranging from 18.5 to 75.8% was reported; domestic violence by an intimate partner alone had a rate of 15.5 to 70.9%, while violence by non-partners ranged between 5.1 and 64.6% (Oche et al., 2020). Moreover, the study conducted in Eldoret, Kenya, showed that the prevalence of intimate partner violence in pregnancy (IPVp) is 34.1%. In this study, the prevalence of physical or sexual violence in pregnancy is 22.8% among the women included in the study. In this study, up to 46 (36.5%) of the survivors of IPV in pregnancy were slapped or had something that could hurt thrown at them (Luhumyo et al., 2020). The prevalence of intimate partner violence during pregnancy is the highest in Africa (Shamu et al., 2011).

A systematic review and meta-analysis conducted in Sub-Saharan Africa in 2020 indicates that the prevalence of gender-based violence, including intimate partner violence, is higher compared to other regions (Muluneh et al., 2020). In this systematic review, the prevalence of IPV in sub-Saharan Africa is 44%. Similarly, the prevalence of emotional, physical, and sexual intimate partner violence is 29.4%, 25.9%, and 19%, respectively, in Sub-Saharan Africa (Muluneh et al., 2020).

According to study findings in Ethiopia, the magnitude of intimate partner violence during pregnancy is one of the highest in the world, ranging from 26% to 65% (Abay Woday Tadesse et al., 2018, Adhena et al., 2020, Alebel et al., 2018, Alhusen et al., 2017, Berhanie et al., 2019, Gebreslasie et al., 2020).

Intimate partner violence cannot be attributed to a single factor. Individual, relational, community, and societal factors influence the experience of IPV during pregnancy, according to the ecological model of violence against women (Fulu and Miedema, 2015). In most prior studies in Ethiopia, mostly the individual factors of women and their partner were identified, including young age of women, higher age gap between couples, low educational status of women, low educational status of intimate partner, being housewife, partner alcohol use, unplanned pregnancy, previous history of miscarriage, stillbirth or pregnancy loss, and living in a rural setting, which were found to be the predictors of IPVp (Adhena et al., 2020; Al Shidhani et al., 2020; Alebel et al., 2018; Ashenafi et al., 2021; Gebrewahd et al., 2020; Lencha et al., 2019; Musa et al., 2020; Studies conducted in Ethiopia were mostly done in clinical settings and were cross-sectional. Few studies were conducted in the community with small sample sizes (Adhena et al., 2020; Gebrewahd et al., 2020; Lencha et al., 2019). A study conducted in Hossana town revealed that the prevalence of intimate partner violence was very high (58.8%) (Temesgen et al., 2019).

One of the 2030 Sustainable Development Goals (SDGs-5) aims to achieve gender equality and empower all women and girls. The specific target under this goal is the elimination of all forms of violence against women and girls. One of the specific targets is the elimination of all forms of violence against women and girls. Despite the high prevalence and growing international attention, there are limited findings on interventions to reduce or control intimate partner violence in pregnancy to achieve a sustainable development goal in 2030 in Ethiopia (UNDP, 2015).

The Ethiopian Community Health Extension Package (ECHEP) and the Ethiopian Health Sector Transformation Plan (HSTP) even did not address the issue of violence against women, despite its adverse maternal and newborn outcomes (Assefa et al., 2019; Health, 2010). Studies conducted in Ethiopia primarily focused on prevalence, correlates, and adverse outcomes, but no study has been found on the intervention to mitigate intimate partner violence during pregnancy. Therefore, this study aims to evaluate the effect of couple-based violence prevention education on intimate partner violence during pregnancy in Hadiya Zone, South West Ethiopia.

**2. LITERATURE REVIEW**

**Introduction**

Intimate partner violence takes the lion share of violence against women (VAW) and is conceptualized as a gender-based human rights violation (McQuigg, 2016). Violence against women, particularly intimate partner violence, is a global public health issue that affects 1 in 3 women in their lifetime. Globally, one in three women (30%) of all women who had been in relationships experienced physical and/or sexual violence. Intimate partner violence during pregnancy has been associated with adverse fatal and non-fatal health outcomes for pregnant mothers and their newborns (WHO, 2011a). Violence against women not only affects the survivors and their families, but it also has social and economic costs (Rivara et al., 2007). Intimate partner violence during pregnancy has serious short-term and long-term health consequences, including maternal mortality (WHO, 2011a).

**Prevalence of intimate partner violence (IPV)**

In 2010, the global prevalence of physical and/or sexual intimate partner violence was 30.0% among women aged 15 and above in their lifetime (K. M. Devries et al., 2013). According to the 2010 WHO multi-country report, more than 25% of women of reproductive age (15–49) experience intimate partner violence at least once in their lifetime. The highest life-time prevalence of physical and/or sexual intimate partner violence among ever-partnered women in Africa, according to the WHO multi-country report, is about 37%, and the lowest (22%) is in low-income countries (WHO, 2010).

Intimate partner violence is experienced all over the world, but it is unacceptably high in Sub-Saharan Africa (SARA COOLS and KOTSADAM, 2017; Seidu et al., 2021). A multilevel analysis of demographic and health surveys conducted among 25 countries in Sub-Saharan Africa indicates the highest prevalence of physical, emotional, and sexual intimate partner violence at 29.3%, 28%, and 11.5%, respectively. In this multilevel analysis, the highest prevalence of physical and emotional intimate partner violence is observed in Sierra Leone (50.0% and 45.9%, respectively) and the lowest in Comoros (5.7 and 7.9%, respectively) (Seidu et al., 2021). Additionally, systematic reviews and meta-analyses of African studies have shown that the prevalence of intimate partner violence during pregnancy is 15.2%, ranging from approximately 2.3% to 57.1% (Shamu et al., 2011).

Analysis of data from 19 countries on intimate partner violence during pregnancy revealed that the prevalence ranges from 2.0% in Australia, Cambodia, Denmark, and the Philippines to about 14% in Uganda among ever-pregnant women. The analysis revealed that the prevalence of intimate partner violence during pregnancy is higher in Africa and Latin American countries compared to European and Asian countries (Devries et al., 2010). Similarly, an analysis of the Demography and Health Survey of 16 countries revealed that the prevalence of IPV is 30.6%. It is higher (40% in Uganda) but lower (12.7% in South Africa) (Ahinkorah, 2021).

A study in Edoret, Kenya, revealed that the overall prevalence of intimate partner violence during pregnancy was 34.1%. In this study, the prevalence of physical, sexual, and psychological intimate partner violence was 22.8% and 27.4%, respectively (Luhumyo et al., 2020). A Systematic Review and Meta-Analysis of Cross-Sectional Studies by Muluneh et al. (2020) found that the pooled prevalence of IPV among women was 44%, but the past-year IPV was 35.5%. The magnitude of psychological, physical, and sexual abuse was about 30.0%, 26.0%, and 19.0%, respectively, in the review done by Muluneh et al. (2020).

Several prevalence studies on intimate partner violence were conducted in different corners of Ethiopia (Abay Woday Tadesse et al., 2018, Adhena et al., 2020, Alebel et al., 2018, Chernet and Cherie, 2020, Tesfa et al., 2020). A study conducted in Central Ethiopia has shown that the overall prevalence of intimate partner violence among women in their lifetime and recent years is 77.0% and 62.4%, respectively (Tesfa et al., 2020). Another study conducted in Axum town, Northern Ethiopia, found that intimate partner violence during the COVID-19 pandemic was 24.6% (Gebrewahd et al., 2020). In Ethiopia, a systematic review and meta-analysis by Alebel et al. (2018) showed that the prevalence of intimate partner violence during pregnancy was 26.1%. It is somewhat lower than individual empirical studies done in different parts of Ethiopia. Chernet and Cherie (2020) found nearly similar rates of intimate partner violence among women in Ethiopia.

A recent cross-sectional study by Adhena et al. (2020) established that the overall prevalence of intimate partner violence during pregnancy was 37.5%. But variations in the prevalence of psychological (25.1%), sexual (17.7%), and physical (13.4%) intimate partner violence during pregnancy were observed (Adhena et al., 2020). Furthermore, the prevalence of domestic violence was 64.6%. This study revealed that physical, psychological, and sexual intimate partner violence were 44.1%, 39.1%, and 23.7%, respectively (Yohannes et al., 2019). A study conducted among 612 pregnant women revealed that the prevalence of at least one type of intimate partner violence during the recent pregnancy was 59.0%. Separately, the prevalence of psychological and emotional violence, sexual violence, and physical violence was 33.0%, 36.3%, and 20.3%, respectively (Lencha et al., 2019).

A systematic review and meta-analysis on antenatal depression and its adverse outcomes by Fekadu Dadi et al. (2020) revealed that the prevalence of antenatal depression was one of the highest in low- and middle-income countries, at 34.0% and 22.7%, respectively (Fekadu Dadi et al., 2020). Ethiopia is one of the countries with the highest maternal depression rates (Beyene et al., 2021; Getinet et al., 2018). A systematic review and meta-analysis done by Getnet et al. (2018) in Ethiopia synthesized the pooled prevalence of antenatal depression at 23.6% (Getinet et al., 2018).

**Knowledge and attitudes toward IPV**

Evidence recommends that IPV knowledge and attitude influence its experience or perpetration. A study in Gambia revealed that about 77.0% had good knowledge of intimate partner violence. 87 percent of subjects had a negative attitude towards IPV (Jatta and Ouedraogo, 2021). In a study done by Oche et al. (2020) in Sokoto, Northwest Nigeria, about 90.0% of participants had good knowledge of IPV (Oche et al., 2020).

**Factors associated with IPV**

Individual, family, community, and societal factors are related to IPV in pregnancy. Risk factors for intimate partner violence during pregnancy are often almost similar to risk factors for intimate partner violence in general (Al Shidhani et al., 2020; Alebel et al., 2018; Ashenafi et al., 2021; Kiranmai Devineni et al., 2018).

Maternal and male partners' ages have been associated with experience with IPV. Mothers under the age of 30 were at risk of partner violence in the EDHS analysis report (Chernet and Cherie, 2020).

Several factors have been reported as factors associated with IPV. In Sokoto, Northwest Nigeria, it has been found that partner alcohol consumption is significantly associated with the experience of intimate partner violence in pregnancy. In this study, pregnant women whose partners consume alcohol are more likely to experience IPV during pregnancy compared to those whose partners do not consume alcohol (Oche et al., 2020). Similarly, a cross-sectional study conducted among pregnant women in Ofla District, Ethiopia, found that women with alcoholic partners are more likely to experience intimate partner violence in pregnancy compared to women with non-alcoholic partners. This is supported by a cross-sectional study by Testa et al. (2020) in Ambo District, Ethiopia. Women whose partner takes alcohol were more likely to experience intimate partner violence compared to women whose partner doesn’t take alcohol (Tesfa et al., 2020). Pregnant women whose husbands drank alcohol were three times more likely to experience IPV compared to pregnant women whose husbands did not drink alcohol in a cross-sectional study conducted in Bale Zone, South West Ethiopia (Lencha et al., 2019).

In a study done by Luhumyo et al. (2020) in Eldoret, Kenya, it was revealed that lower than tertiary education level is significantly associated with experience of psychological intimate partner violence. Women with lower than tertiary educational levels are more likely to experience intimate partner violence compared to their counterparts with tertiary education (Luhumyo et al., 2020). In an analysis from the 2016 Ethiopia Demographic and Health Survey by Chernet and Cherie (2020), it was found that the primary and secondary educational level of the mothers was the predictor of intimate partner violence in Ethiopia (Chernet and Cherie, 2020).

Intimate partner educational status was seen as a predictor of intimate partner violence. A systematic review and meta-analysis on intimate partner violence and associated factors in Ethiopia has found that pregnant mothers whose intimate male partner is unable to read and write are about three times more likely to experience IPV as compared to their literate counterparts (Alebel et al., 2018). In contrast, in a study done in Oman, there was no significant association between IPVp and pregnant women’s partner's educational status (Al Shidhani et al., 2020).

A systematic review and meta-analysis of intimate partner violence and associated factors among pregnant women by Alebel et al. (2018) in Ethiopia found that motherseducational status, partners’ educational status, and intimate partners’ alcohol use were significantly associated with IPV among pregnant women. In this analysis, the likelihood of intimate partner violence experience was two times higher among mothers who were unable to read and write as compared to their literate counterparts (Alebel et al., 2018).

Unintended pregnancy was the predictor of IPV studies in Oman, Bale, Ofla District, Tiray, Harari Region, and northern Ethiopia (Adhena et al., 2020; Al Shidhani et al., 2020; Gebrewahd et al., 2020; Lencha et al., 2019; Musa et al., 2020). A prospective observational study of pregnant women in Oman revealed that unplanned pregnancy was seen as a risk factor for IPV. Similarly, pregnant women who had an unwanted pregnancy were about three times more likely to encounter IPV than those who wanted the pregnancy, according to a cross-sectional study done in the Bale Zone, Southwest Ethiopia (Lencha et al., 2019). A contrasting result was reported in the systematic review and meta-analysis done by Alebel et al. (2020), which means the likelihood of IPV occurrence was two times higher among mothers’ who were unable to read and write as compared to their literate counterparts. That means unplanned pregnancy was not significantly associated with intimate partner violence (Alebel et al., 2018).

Findings from the WHO multi-country study on women’s health and domestic violence have resulted in the finding that secondary and above education, high socioeconomic status of women, and formal marriage were protective factors of recent intimate partner violence. Whereas, young age, cohabitation, alcohol use, attitudes supportive of wife beating, having outside sexual partners, and experiencing childhood abuse were the risk factors of intimate partner violence (Abramsky et al., 2011). History of adverse pregnancy outcomes, partner age 30 and above, partner aggressive behavior, partner who chews chat and partner smoking status were significantly associated with intimate partner violence during pregnancy in a study done by Lencha B. et al. (2019) in Bale Zone, Southwest Ethiopia. Pregnant women whose partner was aged 30 years and older were about two times more likely to experience intimate partner violence during pregnancy (Lencha et al., 2019).

Studies have found that being single, fear of pregnancy complications, history of chronic illness, unintended pregnancy, history of stillbirth, intimate partner violence, income, occupation, history of the previous mental disorder, antenatal follow-up, complications during pregnancy, age of mother during pregnancy, conflict, and social support were found to be significantly associated with antenatal depression (Agostini et al., 2015; Beyene et al., 2021; Fekadu Dadi et al., 2020; Getinet et al., 2018).

**Intimate partner violence during pregnancy and maternal outcomes**

Experiencing intimate partner violence during pregnancy has been associated with a multitude of fatal and non-fatal health consequences for the mother and her baby (Alhusen et al., 2015; WHO, 2011a). Findings from research have shown that experiencing intimate partner violence during pregnancy leads to delayed early initiation of antenatal care or missed antenatal care appointments compared to non-abused counterparts. Findings on the association between intimate partner violence and maternal health care utilization from 36 national household surveys suggested that women experiencing intimate partner violence decreased maternal health care utilization in developing and middle-income countries. This review revealed that women who experience IPV during pregnancy were less likely to utilize four or more ANC visits, the number of ANC visits, and delivery care compared to their non-experienced counterparts (Leight and Wilson, 2021). In a similar way, a study conducted in Addis Ababa, Ethiopia, has shown that women who experienced physical intimate partner violence from male partners in their relationship are less likely to attend WHO-recommended four-month antenatal care compared to those who did not experience intimate partner violence. Likewise, women who experienced emotional and sexual IPV were less likely to attend the recommended ANC, which was attended by skilled delivery attendants, compared to their non-experienced counterparts (Leight and Wilson, 2021).

Smoking, alcohol use, substance use like chat chewing and inadequate weight gains during pregnancy are well-known risk factors for adverse neonatal outcomes. Similarly, study done by Bailey and Daugherty (2007) in US, found that Physical IPV during pregnancy increased rates of pregnancy smoking, increased rates of alcohol, marijuana, and harder illicit drug use around the time of conception. Moreover, the experience of psychological IPV during pregnancy significantly increased rate of alcohol use and an increased rate of pre-pregnancy obesity (Bailey and Daugherty, 2007). Researches have demonstrated that IPV during pregnancy is significantly associated with maternal depression, anxiety and stress, miscarriages, still birth and abortions (Afiaz et al., 2020, Alhusen et al., 2015, Belay et al., 2019, Kiranmai Devineni et al., 2018)

A prospective observational study conducted among pregnant women in India revealed that intimate partner violence directly contributed to two maternal deaths and six severe acute maternal morbidities (Kiranmai Devineni et al., 2018). Similarly, exposure to intimate partner violence is the leading cause of homicide death in women globally (K. M. Devries et al., 2013). About 38% of all murders of women are committed by intimate partners (WHO, 2011a).

A cohort study and multivariate analysis by Martin-de-Las-Heras et al. (2019) in Spain revealed that psychological IPV during pregnancy was significantly associated with urinary tract infection, vaginal infection, and spontaneous pre-term labor. Similarly, in this follow-up study, physical intimate partner violence was significantly associated with antenatal hospitalization, and mothers who experienced IPV during pregnancy had higher odds of developing complications (Martin-de-Las-Heras et al., 2019). A prospective observational study done in India has shown that women who experienced intimate partner violence in pregnancy were more likely to develop placental abruption, pre-term labor, and severe maternal morbidity compared to women who did not experience IPV during pregnancy (Kiranmai Devineni et al., 2018).

**Intimate partner violence during pregnancy and neonatal outcomes**

Studies suggest that adverse neonatal outcomes, particularly pre-term birth and low birth weight, are associated with IPV during pregnancy (Alhusen et al., 2015; Kiranmai Devineni et al., 2018). Likewise, a case-control study done in Addis Ababa, Ethiopia, found that women who experienced intimate partner violence during pregnancy were more likely to experience low birth weight and preterm birth. In addition to IPV, women who have been exposed to physical intimate partner violence during pregnancy are more likely to experience low birth weight and preterm birth (Berhanie et al., 2019). Contrasting evidence was recorded in a prospective cohort study by Pun et al. (2019) in India. There was no significant association between exposure to any domestic violence during pregnancy and the risk of a low-birth-weight baby or birth by cesarean section (Pun et al., 2019).

Several studies have shown the association between intimate partner violence during pregnancy and prenatal or neonatal death (Ahmed et al., 2006; Ashenafi et al., 2020; Memiah et al., 2020). Evidence from India by Ahmed et al. (2006) has found that women who experienced domestic violence have a higher risk of perinatal and neonatal mortality than births among mothers who had not experienced domestic violence. In contrast, the study found that there is no significant association between experiencing domestic violence and post-neonatal and child mortality (Ahmed et al., 2006). A multi-country analysis of East Africa by Memiah et al. (2020) has also found that children born to women who experienced intimate partner violence in pregnancy are more likely to die as newborns and infants (Memiah et al., 2020). Furthermore, a population-based matched case-control study found that sexual violence during pregnancy and husband-control behavior are significantly associated with neonatal mortality. But physical and emotional intimate partner violence during pregnancy is not significantly associated with neonatal mortality (Ashenafi et al., 2020).

**Interventions for intimate partner violence**

A meta-analysis on psychosocial interventions for intimate partner violence in low and middle-income countries by Turner et al. (2020) found that psychosocial interventions reduced any form of intimate partner violence by 27% at the shortest follow-up and 25% at the longest follow-up. The analysis also revealed that the intervention reduced physical violence by 22% at the shortest and 27% at the longest follow-up, sexual violence by 23% at the longest follow-up, but had no significant effect at the shortest follow-up (Turner et al., 2020). A systematic review of ICT-based interventions for intimate partner violence by El Morr and Layal (2020) evidenced that the intervention was effective in the prevention, disclosure, and screening of intimate partner violence (El Morr and Layal, 2020). Additionally, intimate partner violence during pregnancy and antenatal depression were significantly reduced by a psychosocial intervention done in Tanzania (Mutisya et al., 2018).

Randomized controlled trials of systematic reviews of workplace interventions for intimate partner violence by Adhia et al. (2019) noted that the interventions provided at work have improved awareness of IPV, increased the provision of information to victims, and increased the willingness to intervene if an employee may be experiencing IPV (Adhia et al., 2019). A systematic review of family-centered interventions for intimate partner violence done by Jill Ryan and Roman (2019) evidenced that there was a reduction in IPV (Jill Ryan and Roman, 2019).

A cluster randomized controlled trial to evaluate the effect of gender transformative intervention on intimate partner violence in Tanzania has found that the intervention has a positive effect on women’s beliefs and attitudes towards physical and emotional intimate partner violence (Harvey et al., 2021). Social and financial supports were significantly associated with intimate partner violence among pregnant women in a prospective cohort study in Moshi Municipality, Northern Tanzania. Pregnant women who had no financial support were more likely to experience IPV and repeated episodes of abuse during pregnancy. In this study, social support in the form of communication—talking to a member of the family—decreased the odds of IPV and repeated episodes of IPV during pregnancy (Sigalla et al., 2017).

1. **Theoretical framework**

**3.1. The ecological theory**

The ecological theory will be used as a framework to better understand community perspectives, risk factors, and interventions towards intimate partner violence during pregnancy (Di Napoli et al., 2019). Violence is the result of the complex interplay of individual, relationship, community, and social factors (Adhena et al., 2020).

The ecological perspective is a holistic and multidimensional model for understanding individual, relational, community, and societal factors influencing the experience of intimate partner violence during pregnancy (Gashaw et al., 2019). Understanding how these factors are related to violence is one of the important steps in the public health approach to preventing violence. It is the best recommended theory for planning and implementing interventions controlling and/or reducing intimate partner violence (Etienne G. Krug et al., 2002; Gashaw et al., 2019). This model will be used to explore the community's perceptions of intimate partner violence during pregnancy.

**Individual**

**Socio-demographic**

Age, age at marriage, age gap residence, educational level occupation

Religion,

Household wealth index

**Reproductive**

Pregnancy intension, parity, sex of preceding birth, previous ANC follow-up, previous facility delivers,

**Behavioral**

Smoking, chat chew, smoking

\\

,

Knowledge on IPV

IPVp

Attitude,

Controlling towards IPV

**Relational**

Marriage type

Disparity in educational attainment

Duration of marriage

**Couple Based Violence Prevention**

**Education**

**Community**

Social support

Inequitable gender norms

Autonomy (household decision making)

Self-efficacy

**Societal level**

Socioeconomic status

Figure 2 A Conceptual frame work adapted from Glanz K. et al., 2008.

**4. Significance of the study**

Violence against women is a worldwide public health concern. The study generates evidence that will give insight into work toward achieving the Sustainable Development Goals (SDG-5) target of eliminating all forms of violence in Ethiopia.

The evidence generated by this study may be useful to the health departments at the zonal level, the Regional Health Bureau, the Ministry of Health (MoH) at the federal level, and other stakeholders, including NGOs concerned with maternal health. Based on the study findings, it will open an opportunity to recommend couple-based gender education to be included in the health extension package of Ethiopia (HEP) and will be considered part of the Health Sector Development Plan (HSDP).

**5. Research question, hypothesis, and objectives**

**5.1 Research Question**

What is the difference in magnitude of IPV between couples who receive violence prevention education and couples who receive usual care?

**5.2 Research Hypothesis**

HO: Is there no difference in the magnitude of intimate partner violence between couples who receive violence prevention education and couples who receive the usual care provided by health extension workers?

H_A_: The proportion of intimate partner violence during pregnancy is lower among women who receive Couple-Based Violence Prevention Education (CBVPE) than among couples who receive usual care from health extension workers.

**5.3 Research objectives**

**5.3.1 General Objective**

To evaluate the effect of Couple-Based Violence Prevention Education (CBVPE) on intimate partner violence during pregnancy in Hadiya Zone, South-West Ethiopia

**5.3.2 Specific Objectives**

1. To assess the prevalence and associated factors of IPV during pregnancy
2. To explore the community's perspectives of IPV during pregnancy
3. To evaluate the effect of couple-based violence prevention education on couples knowledge, attitudes, and controlling behavior towards IPV during pregnancy
4. To compare the effect of couple-based violence prevention education on the experience of IPV during pregnancy
5. To compare the effect of couple-based violence prevention education on women’s autonomy and self-efficacy

1. **Methods and Materials**

**6.1 Study area**

We will conduct the study in Hadiya Zone. It is one of the administrative zones in the Southern Nations, Nationalities, and Peoples Region (SNNPR). Its capital, Hossana Town, is located 235 km south-west of the capital city, Addis Ababa. Hadiya Zone has 13 woredas (districts) with an area of 3542.66 square kilometers and a population density of 444 people per square kilometer. The zone has four town administrations and 329 kebeles (sub-districts). According to 2021 Hadiya zone statistics, it has a total population of 1,767, 390 of whom 873,091 (49.4%) are males and 894,299 (50.6%) are females. Hadiya zone is bounded by 4 regions, namely: Oromia, Silte, Gurage, and Kembata, and one special zone, Yem (ZHD, 2021).

About 90% of the population lives in the rural part of the zone, and the dominant ethnic group is Hadiya. The zone has one comprehensive specialized hospital, three primary hospitals, sixty-one health centers, and 311 health posts (ZHD, 2021). The study will be conducted in four woredas of the zone, randomly selected from 13 woredas. Four-woredas (destricts) have 126 kebeles (the lowest administrative unit).

**6.2 Study period**

The study will take 8 months of follow-up (6 months of intervention + endline survey after 2 months of intervention) and be carried out for months from August 1, 2022, to April 30, 2023. The outcome information will be collected at baseline (≤12 weeks' gestation) and end-line (after 8 months of follow-up).

**6.3 Source population**

All couples (male partners and pregnant women of reproductive age) in Hadiya Zone

**6.4 Study population**

All couples whose wife is pregnant (1^st^ trimester) and in the child-bearing age in selected intervention and control clusters (kebeles)

**6.5 Inclusion and inclusion Criteria**

**Inclusion**

- Coupes whose wives are pregnant (1st trimester)
- Couples at least having one live birth
- Couples whose wives are of childbearing age
- Couples who lived in the study area at least six months before the study
- Couples living in the study area until 2 months after giving birth
- Couples whose wives' interpregnancy interval is less than two years
- Couples willing to be visited at home

**Exclusion**

- Couples having severe medical illnesses
- Couples with hearing or communication problems
- Couples with a plan to move out of the intervention and control clusters in the next 8 months

**6.6 Study variables**

**6.6.1 Dependent variables**

**Primary outcome variable**

- Intimate Partner Violence (experience versus not experiencing IPV)

**Secondary outcome variables**

- Knowledge, attitudes, and control towards IPV during pregnancy
- Knowledge: Good versus Poor Knowledge,
- Attitude: supportive versus not supportive attitude toward IPV
- Controlling behavior: yes versus no
- Women’s autonomy and self-efficacy

**6.6.2 Independent or ecological model variables**

**Individual**: wife age, wife age at marriage, spousal age difference, educational status of couple, occupation of couples, woman’s parity, alcohol use, sex of previous children, birth order smoking, previous ANC and place of delivery.

**Relational:** marriage type, duration of marriage, disparity in education level

**Community**: presence of social support and inequitable gender norms

**Societal**: household wealth index

**6.7 Study Design**

Three study designs will be used in the study. Study designs differ according to the study type.

**Qualitative study**: **FGD and in-depth interview (IDI)** methods of qualitative study will be used at baseline. The aim of this objective is to explore community perceptions of intimate partner violence during pregnancy.

**Quantitative study:** A two-arm parallel-group cluster randomized controlled trial with a 1:1 allocation ratio is designed to examine whether couple-based violence prevention education

provided by HEWs reduces intimate partner violence during pregnancy in the Hadiya Zone, Southwest Ethiopia. Clusters will be found in selected districts of Hadiya Zone.

**6.8 sample size**

**Qualitative study:** the sample size for a qualitative study will be determined by information saturation (Dworkin, 2012). But, for planning purposes, eight (8) Focus Group Discussions (FDG)( at least two FGDs from male partners (husbands of pregnant women), two FGDs from religious leaders, two FGDs from community leaders, and two FGDs from the women's development army) will be considered. Moreover, six in-depth interviews (IDI) among pregnant women and 70**participants** will be involved in FGD, and IDI will be involved in order to explore community perspectives towards IPV in pregnancy.

**Quantitative study (CRCT):** sample size is calculated by using the following parameters: The prevalence of IPV during pregnancy (p) was  37.5% in a study conducted in Tigray, Ethiopia (Adhena et al., 2020). The expected decline of IPVp following the intervention will be 20%, with 80% power, a 95% confidence interval, an intra-correlation coefficient (ICC) variation of 0.05, a loss to follow-up of 20%, and a design effect of 2.3. Hence, the sample size is based on Stata version 16.

Design Effect (DE) = 1 + ρ (m-1)

Where,

DE=Design effect,

ρ= intra-cluster correlation coefficient = 0.05

m=number of participants per cluster = 27.

DE=1+0.05(27-1) =2.3

Attrition (20%)

=216 couples per control group and 216 couples per intervention group; sample size =432 couples, making a total of 864 participants.

Total cluster (k) = 16 kebeles (clusters)

## 6.9 Sampling Procedure

We will use the multi-stage cluster sampling to select eligible couples in the study area. Four districts (Soro, Lemo, Analemo, and Duna) are selected randomly from the 13 districts in the zone, which consists of 116 rural kebeles. Distance and geographical accessibility will be considered to select interventions and control kebeles. At least one kebele will be left between the kebele included in the trial to serve as a buffer zone between the two arms in order to avoid information contamination. Out of 116 kebeles, 49 non-adjacent and accessible kebeles are identified. Finally, Out of 49, 16 rural kebeles—the lowest administrative units from the four districts—will be selected by simple random sampling technique for the intervention and control groups. In each cluster, there will be 27 pregnant women with their husbands who will participate, making 216 couples per arm (Figure 4).

16 clusters

Hadiya Zone (13 districts)

By simple random sampling

By simple random sampling

Soro, Lemo, Analemo and Duna districts (116 kebeles)

49 non-adjacent and accessible kebeles (clusters)

8 intervention clusters

8 control clusters

27 eligible couples per cluster

27 eligible couples per cluster control clusters

216 eligible couples per arm

216 eligible couples per arm

Figure 3: diagrammatic representation of sampling procedure

**Qualitative part:** we will deploy purposive sampling techniques for the FGD and IDI. Women support groups, male partners, religious leaders, and community leaders will be selected for the FGD. In-depth interviews (IDI) will be carried out among pregnant women until the information saturation

**5.10 Operational Definition**

**Couple**: a man and woman living together, whether married or not.

**Intimate partner**: a current husband or cohabiting partner (living in the same house without formal marriage)

**Intimate partner violence**: pregnant women who reported any violence in the form of physical, psychological, or sexual violence, or any combination of the three, by their husband during pregnancy (Trott et al., 2017).

**Physical violence** refers to when a pregnant woman reports one or more acts of slapping or throwing something at her that could hurt her, pushing, shoveling, or pulling her hair, hitting her with her fist or with something else that could hurt her, kicking, dragging, or beating her up, attempting to choke or burn her on purpose, and threatening to use or actually use a gun, knife, or other weapon during the most recent pregnancy (WHO, 2010).

**Sexual violence:** refers when pregnant women reports that they experience one or more physically forced her to have sexual intercourse with him when she did not want to, forced her with threats or in any other way to perform sexual acts she did not want and physically forced to perform any other sexual acts she did not want toduring the most recent pregnancy (WHO, 2010)

**Pregnancy Screening**

All women residing in the intervention and control arms will be screened using a pregnancy screening questionnaire adapted from Nega A et al. (2010, Kersa, Ethiopia. “If the answer to at least one of the six pregnancy screening questions is ‘yes’, pregnancy will be ruled out. Women who responded in a way that suggested the possibility of being pregnant will be asked to provide a urine sample for a pregnancy test, which will be done using a dipstick in the respondent’s home (Assefa et al., 2013).

Table 1: pregnancy screening questions

| S. No | Items | Yes | No |
| --- | --- | --- | --- |
| 1 | During the last four weeks, did you give birth? |  |  |
| 2 | Do you fully breastfeed or are you less than six months postpartum and have not experienced menstrual bleeding since bearing your child? |  |  |
| 3 | During the last week, did your last period begin? |  |  |
| 4 | In the last week, did you experience a miscarriage or an abortion? |  |  |
| 5 | Since your last period, have you avoided sexual intercourse? |  |  |
| 6 | Have you been consistently and appropriately using a safe method of contraception (pills, injectables, and Norplant)? |  |  |

**6.11 Recruitment of clusters or study participants**

Clusters included in the intervention and control groups will be selected from the four districts in Hadiya zone, namely Soro, Lemo, Ana Lemo, and Duna woredas. The couple violence prevention education will be done in eight intervention clusters of the four districts. Similarly, eight clusters from the four districts will be assigned to the control group.

**Requirement of the study participants (n=426 couples**)

Intervention group (n=213 couples)

Control group (n=213)

Control group (study 1)

8 clusters (kebeles)

n=213 couples (213 pregnant women+ 213 husbands)

GBVP education (study2)

8 clusters (kebeles)

N=213 couples (213 preg. Women+213 husbands)

Control group (study group 1)

- No intervention will be provided from the research team
- Health Extension workers in the clusters continue to provide HE on Health extension packages.

Violence prevention education (study group 2)

- Monthly couples (pregnant women and husbands) education sessions from 1^st^ trimester to term(delivery)
- Data collection at
- Baseline and end-line(two months after intervention)

 Intervention and determination of IPV experience, Knowledge, attitude and controlling towards IPV and wome’s autonomy and self-efficacy

**Randomization of clusters**

Figure 4: diagrammatic representation of study activities

## 6.12 Randomization

After getting permission from Kebele administrators (gatekeepers), clusters will be used for randomization into intervention and control group. Kebeles in districts will form the units of randomization for the trial. Randomizations of the clusters will be performed before recruiting the participants into the two arms. All kebeles found in four districts will be listed. Each cluster will be given an individual code and an alphabetical list. A statistician who is blind to the study groups will perform the randomization. The statistician will assign the 16 clusters into 8 intervention and 8 control arms by applying permuted block randomization methods. Randomization into two study groups will be based on a 1:1 ratio.There will be adequate buffer zone to avoid information contamination

## 6.13 Blinding

Neither the Health extension workers nor the participants will be informed about the intervention hypothesis

**6.14 Group Information**

There will be two arms or groups of information in the selected districts of the zone. There will be an intervention group (who receive couples-based violence prevention education) and a control group (who receive usual care). Hence, the clusters will be assigned into two arms by randomization. An intensive health education message will be delivered to the intervention arms based on the baseline findings. The type of unit under this study will be clusters. The four selected districts have a total of 116 clusters. Out of 49 eligible clusters, 16 clusters will be selected by simple random sampling. In each cluster, there might be 27 couples (husbands and pregnant wives) who have the potential for the health education message. Therefore, an intervention arm consists of 8 clusters, and a control arm consists of 8 clusters. There will be 27 couples per cluster, forming a total of 216 couples (216 male partners and 216 pregnant wives) in the intervention group and 216 in the control group, making 432 couples, which will be followed in the intervention period.

**6.15 Description of the Intervention**

The purpose of the study is to provide community-based health education to couples on violence prevention to reduce intimate partner violence during pregnancy. A group health education on violence prevention, including intimate partner violence, for couples in the intervention group will be provided in a place center for the participants. 16 kebels found in the four districts will be the study clusters.

A package of health education messages on violence prevention will be provided to couples living in the intervention arm. The package was adapted from a psychosocial education study conducted among couples (Mutisya et al., 2018) and gender transpormative education on intimate partner violence in Tanzania (Harvey et al., 2021). The health education messages will be conducted by health extension workers who are working in intervention clusters (kebeles). Eight health extension workers will be selected for the intervention (CBVP education). The following criteria will be used to recruit health extension workers:

- Health Extension Workers who are female
- able to speak Amharic and the local language (Hadiyisa).
- willing to participate in the study period
- Good interpersonal and communication skills
- Having participated in a survey previously

**Consort Diagram**

The study protocol containing the detailed information is depicted in the consort diagram below (Figure 5).

116 rural kebeles found in Soro,Lemo, Analemo and Duna Districs

49 eligible kebeles

16 kebeles selected

Randomization of 16 kebeles (intervention and control arms)

Allocation

Intervention arm (8 clusters)

Control arm (8 clusters)

Participants will be assessed for eligibility criteria

 Participants will be assessed for eligibility criteria

Enrollment

Informed consent and

Baseline data collection

Informed consent and

Baseline data collection

Intervention group receive

CBVPE

Control group continue to receive routine care

Analysis

8 months follow-up survey

Intervention

Identification of couples whose wife is pregnant

Figure 5: Consort flow diagram

**6.15.1 Couple education contents and sessions**

**Control group (study group 1):** no intervention will be provided for these clusters (kebeles) from the research group. However, the control group will not be restricted from the standard health extension package services provided by health extension workers.

**Couples GBV prevention education group (study group 2):** in this group, the couples (pregnant women and their male partners) will be educated on:

- Gender-based violence
- Violence against women
- Intimate partner violence
- Types of intimate partner violence (physical, sexual, and psychological or emotional violence)
- Magnitude of intimate partner violence in pregnancy
- Inequitable gender norms and consequences
- The relationship between intimate partner violence and power and control
- Common triggers of IPV in pregnancy
- Uhhealthy and healthy relationship
- Common triggers of IPV and ways to challenge the triggers
- Effective communication and conflict resolution in relationship

The couple’s education will be provided every fourth week by the health extension workers, assisted by the researcher. Baseline data will be collected from 10 to 12 weeks of gestation. The education services will be provided in groups, and each participant has six contacts with the educator. The contents and sessions for couple education are illustrated below in Figure 7.

**1^st^ session**

13-16 weeks gestatio

- Gender based violence (what is it)
- Violence against women (what is it)
- Intimate partner violence (what is it )
- Types of IPV
- Physical, sexual and psychological
- Magnitude of intimate partner violence

Schedule by weeks

Content ofcouple based violence prevention education

- Adverse maternal and newborn consequences of IPV during pregnancy
- Economic and social consequences of IPV during pregnancy
- Common triggers of IP
- Ways to challenge triggers of IPV

**2^nd^ session**

17-20 weeks

- Healthy and unhealthy behaviors in marital relationship
- Important characteristics of healthy relationships

**3rd session**

21-24 weeks

- Common gender norms for women and men
- Gender and sex
- Inequality gender norms related to IPV during pregnancy
- The ways to challenge inequitable gender norms

**4^th^ session**

25-28 weeks

- Effective communication skills in relationship
- Nonviolent ways of conflict resolution in relationship

**5^th^ session**

29-32 weeks

- Define power and control
- Healthy power balance within relationship
- Benefits of joint decision making
- Seven strategies to joint decision making

**6^th^ session**

33-36 weeks

Figure 6: the schedule for couples education and message contents of intervention group

**6.15.2 Description of education sessions**

**Control group (study group 1):** participants in the control clusters receive routine standard care from the health extension workers**.** Education on violence prevention, including intimate partner violence, will not be provided by the research team. This group will simply receive the health extension package services provided by HEWs.

**Intervention group (study group 2)**: Pregnant women and their husbands will receive couple-based violence prevention education (CBVPE) from the 13–16 weeks of gestation to delivery for six months. The intervention package is aimed at raising knowledge and awareness of gender-based violence, types of intimate partner violence, adverse maternal and new-born outcomes of IPV during pregnancy, common triggers of intimate partner violence during pregnancy and its management, a healthy relationship in marriage, communication, and problem-solving skills. After adequate training, HEWs will provide health education to couples in the intervention arms at the health post in groups. It will be provided every month for six consecutive times. Each session may take 50-60 minutes. All sessions will contain interactive lectures, take-home exercises, and reflection. During class sessions, flip chart paper and posters will be used. It is designed with the intention that they will be carried out in an enclosed workshop space. Neither the HEWs nor the participants will be informed about the intervention hypothesis.

**6.15.3 Intervention fidelity**

Intervention fidelity refers to the degree to which components of intervention are executed as designed. To enhance the intervention's fidelity, we will implement the following actions: a trainer manual has already developed; extensive training will be provided for interventionists (HEWs); spot checks will be conducted by field supervisors during each intervention session; and feedback meetings with interventionists will be held after each session. A fidelity checklist that addresses adherence and intensity of the intervention will be used to evaluate intervention fidelity at the end of each session.

Table 2:summary of the intervention package and implementation process

| **Session** | **Content of the message** | **Dose** | **Strategy of delivery** | **Frequency** | **Compliance parameters** | **Responsible person** |
| --- | --- | --- | --- | --- | --- | --- |
| Session 1  (13-16 weeks) | - Introducing each other - Gender based violence (what is it) - Violence against women(what is it) - Intimate partner violence(what is it ) - Types of intimate partner violence - Physical, sexual and psychological - Magnitude of IPV - Summarization - Introduction on take home-exercise next - Appointment to next session | 60 minutes | • Brain storming • Lecture • Question and Answer  **.**Take-home exercise and reflections | Once | -% couples participated | Researcher, Supervisors & Health extension workers |
| Session 2  (17-20 weeks) | - Refelection on take home exercise and recap - Maternal and new born consequences of IPV - Economic and social consequences - Family and dependents consequences - Common triggers of IPV during pregnancy - Ways to challenge triggers of IPV - Introduction on take-home exercise - appointment to next session | 60 minutes | • Brain storming • Lecture • Question and Answer  **.**Take-home exercise and reflections | Once | -% -% couples participated | Researcher, Supervisors & Health extension workers |
| Session 3  21-24 weeks | - Reflection on take home exercise - Common gender norms for women and men - Gender and sex - Inequality gender norms related to IPV during pregnancy - The ways to challenge inequitable gender norms - Introduction on take-home exercise and appointment to next session | 60 minutes | • Brain storming • Lecture • Question and Answer  **.**Take-home exercise and reflections | Once | -% -% couples participated | Researcher, Supervisors & Health extension workers |
| Session 4  (25-28 weeks) | - Recap and reflection on take home exercise - Healthy and unhealthy behaviors in marital relationship - Important characteristics of healthy relationships - Introduction on take-home exercise and appointment to next session | 55 minutes | • Brain storming • Lecture • Question and Answer  **.**Take-home exercise and reflections | Once | -% -% couples participated | Researcher, Supervisors & Health extension workers |
| Session 5  (29-32 weeks) | - Recap and Reflection on take-home exercise - Define power and control - Healthy power balance within relationship - Benefits of joint decision making - Seven strategies to joint decision making - Take | 60 minutes | • Brain storming • Lecture • Question and Answer  **.**Take-home exercise and reflections | Once | -% couples participated | Researcher, Supervisors & Health extension workers |
| Session 6  33-36 | - Recap and reflection on take home exercise - Effective communication skills in relationship - Nonviolent ways of conflict resolution in relationship   Summarization and close up | 60 minutes | • Brain storming • Lecture • Question and Answer | Once | -% couples participated | Researcher, Supervisors & Health extension workers |
|  | Provision of printed take-home materials | -Poster | - | Delivered during in each training session | -% of couples received posters | Researcher, Supervisors & Health extension workers |
|  | **End line data collection** | - | - | at 8^th^ month of intervention | % pregnant mother and their husbands interviewed | Researcher, supervisors and data collectors |

**6.15.3 Appointment and follow-up for intervention group**

The contact address of each couple included in the study will be taken. The addresses of the couples, including kebele, gote (sub-kebele), house number (if applicable), and telephone number, will be taken by the researcher and the HEWs (educators). At the end of each session, the educator and the participant will agree on the date of the next visit, which will be recorded by the educator in her diary and on a similar diary that will be left with the participants. Before the next visit, the educator reminds the couples about the next session by calling or messaging them. Either of the couples who missed the classroom sessions will be visited by HEWs at their home to rule out the reason for their absence. Then another classroom session will be arranged. At the end of each session, the number of couples who attended will be recorded. Couples in the intervention clusters will be visited three times in case of absence to improve the participants’ compliance. End-line data collection will be carried out at month 8. Every month, HEWs will screen the couples for any dispute or disagreement during a home visit by asking either of the couples if there is any disagreement or dispute due to intervention. If so, she negotiates with the couples. If the dispute still continues in the second screening, she communicates with the principal investigator, and they both resolve the dispute between the couples as much as possible.

## 6.16 questionnaire translation, validity and reliability

**Quantitative data**: An essential step for the questionnaire's validity is the translation of the original English version into a native language (Wild et al., 2005). We'll follow a methodical procedure suggested by Beaton et al. (Dorcas E. Beaton, 2000) to maintain conceptual, experiential, and idiomatic equivalence. Bilingual translators will translate the instruments into Hadiyisa. The instruments will be translated back into English by two bilingual translators from the Department of Hadiyisa, Wachemo University, who are completely blind to the original English version. To establish semantic equivalence, the back-translation is necessary. To combine all language versions and create the pre-final questionnaire for field testing, an expert committee meeting will be organized (Abdulahi et al., 2018). To verify the translation's accuracy, the original and back-translated English versions of the instruments will be compared. Using a technique developed by Skperber et al. (Sperber et al., 2016), each item in the original and back-translated versions will be ranked in terms of comparability of language and similarity of interpretability for the purpose of certifying the translated instrument. Fluent in English experts from the department of foreign languages and literatures will assess the comparisons using Likert scales from 1 (highly comparable/extremely similar) to 7 (not at all comparable/not at all similar). There will be a formal evaluation of the translation for any mean score greater than 3.

Pilot testing of the translated tool will be conducted among 27 couples who will not be included in the actual trial to assess its understanding, comprehension, and appropriateness. Additionally, subject experts will evaluate the content, and the necessary changes will be made (Figure 8). The tools' internal consistency will be tested using Cronbach's alpha. A tool with a Crombach alpha of ≥ 0.7 will be considered reliable(Sharma, 2016). Finally, the questionnaires will be utilized at the study's baseline and completion.

Content Validity

(IPVp)

Forward translation by public health experts /group-1

Forward translation by Language experts/group-2

Backward translation by

Public health experts/group-3

Backward translation by language experts /group 4

Review of 4 version of the questionnaire and final versison (IPVp)

Determine face validity of the pre-final version (IPVp) and revise the questionnaire if necessary

Conduct a pre-test study on the pre-final version (IPVp) and revise the questionnaire if necessary

Final-version (IPVp)-field work

Figure 7:Schematic representation of validation process of the questionnaire (IPVp)

**6.17 outcomes of interest and measurement**

**Dependent variables:** experience of intimate partner violence (IPV) in the form of physical, psychological, or sexual violence from the male partner is an independent variable of the study among couples.

**Physical intimate partner violence**: During data collection, pregnant women will be asked six physical intimate partner violence questions: (i) “Slap or throw something at her that could hurt her”, (ii) “Pushed shoved her or pulled her hair”, (iii) “Hit her with his fist or with something else that could hurt her”, and (iv) "Kicked, dragged, or beaten her up." (v) “Attempted to choke or burn her on purpose” and (vi) "threatened to use or actually use a gun, knife, or other weapon against her” (Abramsky et al., 2011) in the last 12 months and during the most recent pregnancy. If the mother responds to one or more of these, dichotomous dependent variables will be created from these questions.

If a pregnant mother answers one or more of these physical intimate partner violence questions, 1=yes (experienced physical intimate partner violence), and otherwise, 0=not experienced physical intimate partner violence.

**Sexual intimate partner violence**: A pregnant woman will be asked for three sexual intimate partner violence questions: whether her husband (i) physically forced her to have sexual intercourse with him when she did not want to”, (ii) “forced her with threats or in any other way to perform sexual acts she did not want,” and (iii) "physically forced to perform any other sexual acts she did not want to” (Abramsky et al., 2011) in the last 12 months and during the most recent pregnancy. If the pregnant mother answers one or more of these sexual intimate partner violence questions, 1=yes (experienced sexual intimate partner violence), and otherwise, 0=no (not experienced any sexual intimate partner violence since the start of this pregnancy).

**Emotional/psychological violence: a** pregnant mother will be asked the following three psychological violence questions: (i) “say or do something to humiliate her in front of another person”, (ii) "threat to hurt or harm her or someone she cares about,” and (iii) insult or make her feel bad about herself” in the last 12 months and during the most recent pregnancy (Abramsky et al., 2011). If the mother responds to one of these emotional or psychological IPV questions, it will be recorded as 1 = yes; otherwise, 0=no (she has not experienced any emotional or psychological intimate partner violence during this pregnancy).

**Social support: The** Oslo-3 Social Support Scale (OSSS-3) will be used to measure the social support of women. The OSSS-3 consists of three items assessing the level of social support among women. Pregnant women will be asked, **Oslo 1**: How many people are so close to you that you can count on them if you have great personal problems? The responses will be: 1. 'None, 2. ‘1–2’ 3. ‘3–5’ 4, ‘5+’; **Oslo 2:** How much interest and concern do people show in what you do? The responses will be "none."2 ‘little’ 3 ‘uncertain’ 4 ‘some’ 5 ‘a lot’ and **Oslo 3**: How easy is it to get practical help from neighbors if you should need it? The response will be 'very difficult. 2 ‘difficult’ 3 ‘possible’ 4 ‘easy’ 5'very easy. The sum scores range from 3 to 14. Then the sum scores will be categorized as 3–8 (**poor social support**), 9–11 (moderate**social support),** and 12–14 (strong**social support**).

**Knowledge of IPV:** couples will be asked about nine items of knowledge questions on IPV. Couples who scored about 75% or above of the correct answer will be scored as “good knowledge,” and those who scored less than 75% will be scored as “poor knowledge (Oche et al., 2020).

**Attitude towards IPV**: To measure attitude toward intimate partner violence during pregnancy, couples will be asked, “In your opinion, is a husband justified in hitting or beating his wife in the following situations?" (a) “If she goes out without telling him?” (b) “If she neglects the children?” (c) “If she argues with him?” (d) “If she refuses to have sex with him?” (e) “If she burns the food?” Responses of “supportive attitude toward IPVp” to one or more of the scenarios were coded 1; “not supportive attitude toward IPVp" to all scenarios was coded '0'. (Trott et al., 2017).

**Controlling behavior**: couples will be asked six questions: (i) “jealous if she talked with other men”, (ii) ”accused her of unfaithfulness”, (iii) “did not permit her to meet her friends,(iv) “tried to limit her contact with family,(v) “insisted on knowing where she is, and “did not trust her with money(Antai, 2011). Couples responded “yes” to one or more of the control questions coded as 1. Otherwise, it will be coded 'no as 0.

**Women’s autonomy**: women's decision-making Autonomy will be assessed by asking couples who decide on household purchases, including small and large ones, and who decides on visiting relatives and friends. Who decides how to spend the wife's earnings? Who decides the number of children to have? And who decides to obtain health care for themselves? The response options will be “husband only’’, wife only’’ or ‘’both husband and wife”. Women will be considered to participate in decision-making if they make decisions alone or jointly with their husbands. A composite measure will be created using the sums of equally weighted binary input variables. Women will be scored one (1) for answers to each variable that included her (alone or jointly) in decision-making; otherwise, they will be scored zero (0). The index of household decision-making power (autonomy) contains five variables; thus, the respondents will be scored from 0 to 5. Two binary measures from these indexes will be created to indicate women with higher versus lower autonomy. The high and low categories will be created by dividing the sample indexes into approximately half, in which those who score below the mean score are considered to have low autonomy or lower involvement in household decision-making, and those who score above the mean score are considered to have higher decision-making autonomy or higher involvement in household decision-making (Uthman et al., 2009).

**Women’s Self-Efficacy: The** Generalized Self-Efficacy Scale (GSES), which was developed by Albert Bandura, will be used. The General Self-Efficiency Scale (GSES) consists of 10 items scored on a 4-point Likert scale, from 1 (not at all true) to 4 (exactly true). The total scale ranges from 10 to 40, with higher self-efficacy (score of ≥20) and lower self-efficacy (score of <20) (Liping Peng et al., 2020).

**6.18 Data Collectors**

**Data collectors for quantitative study:** 10 diploma-level female nurses will be selected to collect data from the respondents as recommended by WHO to enhance the disclosure of IPVp (WHO, 2007). Data collectors will be trained by the researcher for three days. The selection criteria include:

- Certified diploma female nurses
- Able to fluently speak Amharic and the local language (Hadiyisa).
- Having good interpersonal and communication skills.
- Willing to collect data at the baseline and end of the study

**Data collectors for the qualitative study**: two female moderators will be selected to facilitate FGD. The moderators will be female master’s holders in public health, and those with previous experience in qualitative data collection will be recruited. The same number and criteria as the FGD moderators will be used for the in-depth interview (IDI). The recruitment for the FGD moderators and IDI (interviewers) includes the following criteria: having a master’s degree in the area of public health, being able to speak Amharic and local languages (Hadiyisa), and having previous experience in qualitative data collection.

**Supervisors for quantitative study**: Two supervisors will be recruited for quantitative data collection. When recruiting the supervisor, a master’s degree in a related field and, preferably, previous supervision experience will be used. Their role in the field includes: monitoring health educators and data collectors; making sure education is properly implemented; helping educators and data collectors with any challenges faced during field work; and informing the researcher of any concerns.

**6.19 Training**

The data collectors will be trained by the principal investigators study aim and objectives, roles and responsibilities, research ethics, and interviewing skills. The role plays and demonstration of the interviewing skills will be carried out among the enumerators to improve the interviewing skills and how to record the respondent’s response on the questionnaires.

Three days of training will be provided to qualitative data collectors on moderation skills, the FGD guide, and the interview guide. Demonstration and role play will be an integral part of the training.

Three-day training will be provided to health extension workers by the researcher. The training contents will be based on WHO gender-based violence prevention guidelines, and they are adapted to violence against women education for health extension workers. The health education content for male partners is also included. The training will cover

- Gender-based violence
- Violence against women
- Intimate partner violence
- Types of intimate partner violence (physical, sexual, and psychological or emotional violence)
- Magnitude of intimate partner violence in pregnancy
- Inequitable gender norms and consequences
- The relationship between intimate partner violence and power and control
- Common triggers of IPV in pregnancy
- Uhhealthy and healthy relationship
- Common triggers of IPV and ways to challenge the triggers
- Effective communication and conflict resolution in relationship

**6.20 Research Team**

The research team for this study consists of a principal investigator and three supervisors.

**6.21 Data collection instruments**

**Questionnaires**

**Quantitative study**: structured-interviewer-administered questionnaires adapted from the WHO multi-country study questionnaire will be used to collect information on intimate partner violence in pregnancy (WHO, 2010). The English version of the questionnaire will be translated into the local language (Hadiyisa) by fluent speakers. Then, the questionnaire's local language will be translated back to English by another expert to ensure consistency and accuracy. Four types of questionnaires will be administered at baseline and at the endline for couples (pregnant wife and husband). The data will be collected using a mobile-based application called KoBo Collect. The content of the information under each questionnaire will be as follows:

**Questionnaire for pregnant mothers at baseline and end-line**: the questionnaire will be administered to all pregnant women in the two groups (intervention and control groups) after obtaining informed verbal and written consent. The questionnaire contains individual characteristics of mothers, including age, levels of education, occupation, marriage type, parity, pregnancy intention, and household characteristics. Relational characteristics: duration of marriage and disparity in educational attainment

Moreover, the relational characteristics include the presence of social support and religion. The societal characteristics of pregnant women include women’s autonomy in addition to male involvement in antenatal care. All questionnaires will be administered to the intervention and control groups before any education session. Outcome measures: couples' knowledge, attitude, and control towards IPV; self-efficacy; pregnant women’s experience of IPV in the last 12 months and during the recent pregnancy

**Questionnaire for husbands at baseline and end-line:** similarly, these questionnaires will be administered to husbands of pregnant women in two study groups. The questionnaire consists of information in terms of male partners individual characteristics, including age, levels of education, religion, occupation, marriage type, current alcohol use, current smoking status, women’s autonomy, self-efficacy, and household characteristics. Relational characteristics include the duration of marriage and disparity in educational attainment; community characteristics include the presence of social support. The societal characteristics of male partners include also decision-making power outcome measures such as partner’s knowledge, attitude, and control towards IPV.

**Focus Group Discussion (FGD) guides:** The guide will be used during FGD to collect qualitative information on male partners perspectives towards intimate partner violence, awareness and consequences of IPVs, risk and protective factors of IPV in pregnancy, and myths and misconceptions about intimate partner violence. Themes will be followed by several probes and questions to get a deep insight into the perpetration of IPV. The group discussion may take 60–90 minutes. Besides, the interview guides, tape recorder, and note book will be used during FGDs.

**6.22 Data Quality Control**

To assure the quality of the data, data collectors and supervisors will be trained thoroughly for 3 days. Regular supervision and follow-up will be provided by supervisors and the principal investigator, respectively. In addition, a regular check-up for the completeness and consistency of the data will be made on a daily basis. Every day, collected data by Kobo Collect will be sent to the server (KoKo Toolbox). The questionnaire will be translated into an Amharic version and then back to English by translators who are blind to the original questionnaire. To make the questionnaire standard, reliability and validity tests will be done for items of intimate partner violence, women’s autonomy and self-efficacy, knowledge, attitude, and controlling behavior towards IPV. A pre-test will be carried out in the kebele that will not be included in the actual study in order to validate and standardize the study procedure and research tools.

**6.23 Data Management**

**Qualitative data**: Data transcription and translation will be carried out on the field while the data collection is being conducted. An audio recording of the interview will be transcribed immediately after the interview. The transcription will be translated into English on the same day as the interview. The translation will be read line-by-line for a deep understanding of the data.

**Quantitative Data:** Regular supervision and follow-up will be done by supervisors and the principal investigator. A regular check-up of the data will be made on a daily basis by supervisors. Every day, data collected by KoBo Collect (the mobile application) will be checked for completeness and consistency before being sent to the server (KoBo Toolbox). The supervisor and principal investigator will consider the following points during the checkup of the collected data: the length of time to finish each survey, the collection of Global Positioning System (GPS) points, and their random distribution. Trial participants who are loss to follow up will be listed along with their reasons. Only the study team will have access to the data set. The anonymous record and coding of personal data will not be disclosed to any third party to ensure strict secrecy. Data will be stored on a password-protected data server (KoBo Toolbox) and only accessible by the research team.

**6.24 Data processing and analysis**

**Qualitative Data:** The complete data will be exported to ATLAS Ti. 7.0 for data analysis. The codes will be created initially by independent coders. The codes will be merged after discussion to reach consensus codes. At least two coders will be involved in the coding process and categorization of the codes. These codes will be merged into larger categories and then set into sub-themes. Sub-themes will be reviewed further to develop overarching themes**.** Then the data will be summarized and reported with some important direct quotes.

**Quantitative data:** A complete questionnaire with individual code and specific cluster will be exported from server to SPSS or STATA for analysis. At baseline, descriptive statistics such as proportions, percentages, tables, diagrams, and figures will be used to describe the study population at the intervention and control arms before the intervention starts. The baseline characteristics of the participants and their prior exposure to intimate partner violence will be examined and compared between the intervention and control groups. The chi-square test and independent sample t-test will be used to compare the baseline characteristics for categorical and continuous variables, respectively. After the end-line survey, the proportion of women who experienced intimate partner violence during the trial period will be calculated with a corresponding confidence interval.

We will use the McNemar test to appreciate the differences in outcomes of interest in both intervention and control groups before and after the intervention for categorical data. A paired t-test will be used to compare continues outcome of interest in the intervention and the control groups after and before the intervention. A model of longitudinal data analysis, the GEE (Generalized Estimating Equation), will be used to test the independent effect of the intervention on the outcome of the interest. Data analysis will be performed with an intention-to-treat analysis approach. During the analysis, the effect size, confidence interval, and p-value will be calculated. A model with the smallest Quasi-likelihood under the Independence Model Criterion (QIC) will be used to select the best-fitted model. All tests will be two-sided, and statistical significance will be declared at p < 0.05.

**6.25 Timeline of the study**

Both primary and secondary outcomes will be assessed as illustrated participants’ timeline of enrolment, implementation and assessment schedule (Table 3)

Table 3:Participants’ timeline of enrolment, implementation and assessment schedule

| Activity | Study period | | | | | | | | | |
| --- | --- | --- | --- | --- | --- | --- | --- | --- | --- | --- |
|  | Enrolment | Allocation | Intervention | | | | | | | Close-out |
|  | Month 1 | Month 2 | Months | | | | | | | Month 11 |
|  |  |  | 3 | 4 | 5 | 6 | 7 | 8 | 9 |  |
| Enrolment |  |  |  |  |  |  |  |  |  |  |
| Eligibility screen |  |  |  |  |  |  |  |  |  |  |
| Informed consent |  |  |  |  |  |  |  |  |  |  |
| Allocation |  |  |  |  |  |  |  |  |  |  |
| Baseline assessments |  |  |  |  |  |  |  |  |  |  |
| Interventions |  |  |  |  |  |  |  |  |  |  |
| Endline assessments |  |  |  |  |  |  |  |  |  |  |

**6.24 Ethical Consideration**

Ethical approval of the study will be obtained from the Ethical Review Board of Jimma University. Then, permission letter to precede the study will be obtained Regional health Bureau and Zonal Health department. The research participants will be enrolled into the study after informed verbal and written consent is obtained. Confidentiality of the respondents will also be maintained. The purpose of the study and the right of the respondent not to participate and not to answer the question for which she didn't want to will be explained carefully to respondents before asking informed and written consent to conduct interview. All interviews will be taken place privately at place and time chosen by the respondent. Strict confidentiality will be assured through anonymous recording and coding of questionnaire and placing it in a safe place after they have been collected. For qualitative study, the interview will be carried out in a private place for the purpose of concentration and privacy of participants. Permission to use a tape recorder will be obtained from the participants prior to the interview for all qualitative study participants.

# 7. Work plan

The project will be accomplished within 2022-2023 as indicated below in table 4

Table 4: work plan for effect of couples based violence prevention education on intimate partner violence during pregnancy in Hadiya zone, SNNPR from 2022-2023

| Activity | Responsible body | Time duration(2022-2023) | | | | | | | | | | | | | | | | | | | | | | | | |  |
| --- | --- | --- | --- | --- | --- | --- | --- | --- | --- | --- | --- | --- | --- | --- | --- | --- | --- | --- | --- | --- | --- | --- | --- | --- | --- | --- | --- |
|  |  | 2022 | | | | | | | | | | | | 2023 | | | | | | | | | | | | |  |
|  |  | Aug. | Sept. | Oct. | Nov. | Dec. | Jan | Fab | Mar | Apr. | May | Jun. | Jul. | | Jan. | Fab. | Mar. | Apr. | May. | Jun. | Jul. | Aug. | Sept. | Oct. | Nov. | Dec. | |
| Title selection and approval |  |  |  |  |  |  |  |  |  |  |  |  |  | |  |  |  |  |  |  |  |  |  |  |  |  | |
| Proposal development | Researcher |  |  |  |  |  |  |  |  |  |  |  |  | |  |  |  |  |  |  |  |  |  |  |  |  | |
| roposal defense | Researcher |  |  |  |  |  |  |  |  |  |  |  |  | |  |  |  |  |  |  |  |  |  |  |  |  | |
| Ethical clearance | Researcher |  |  |  |  |  |  |  |  |  |  |  |  | |  |  |  |  |  |  |  |  |  |  |  |  | |
| Pre-test &  Pilot study | Re, SV,DC & Ed |  |  |  |  |  |  |  |  |  |  |  |  | |  |  |  |  |  |  |  |  |  |  |  |  | |
| Baseline data collection | Re, SV, Ed and DC |  |  |  |  |  |  |  |  |  |  |  |  | |  |  |  |  |  |  |  |  |  |  |  |  | |
| Intervention  Datacollection | Re, SV, Ed and DC |  |  |  |  |  |  |  |  |  |  |  |  | |  |  |  |  |  |  |  |  |  |  |  |  | |
| Data entry ,cleaning and analysis | Re and DC |  |  |  |  |  |  |  |  |  |  |  |  | |  |  |  |  |  |  |  |  |  |  |  |  | |
| Manuscript writing and Publications | Researchers |  |  |  |  |  |  |  |  |  |  |  |  | |  |  |  |  |  |  |  |  |  |  |  |  | |
| PhD Dissertation | Researcher |  |  |  |  |  |  |  |  |  |  |  |  | |  |  |  |  |  |  |  |  |  |  |  |  | |

Re: Researcher; SV: Supervisors; Ed: Educators; DC: Data collectors

# 8. Budget

## 8.1 Budget Breakdown

To accomplish this project, about 276,880 birr (two hundred seventy six thousand and eight hundred and eighty Ethiopian birr) will be needed for salary of data collectors, supervisors, data clerk, transportation and communication.

Table 5: Budget breakdown for couple based violence prevention education in Hadiya zone, 2022/2

| S.No. | Budget category | Daily Payment in Birr | Quantity/Number | Number of days | Total cost in Birr |
| --- | --- | --- | --- | --- | --- |
| 1 | **Personal cost** |  |  |  |  |
|  | Data collectors | 300 | 10 | 30 | 90,000 |
|  | Supervisors | 300 | 4 | 30 | 36,000 |
|  | Investigators | 300 | 3 | 30 | 27,000 |
|  | Data entry clerk | 200 | 2 | 30 | 12,000 |
|  | **Subtotal** |  |  |  | **165,000** |
| 2 | **Stationary** | Quantity and unit | Unit cost (ETB) | - |  |
|  | Computer paper | 20pac | 300 |  | 6000 |
|  | Photocopy | Each | 1.50 |  | 15,000 |
|  | Pen | 21 pac | 10.0 |  | 1,050 |
|  | Pencil | 21 pieces | 5.0 |  | 525 |
|  | Eraser | pieces | 4 |  | 120 |
|  | Note book | 1,050 pieces | 30 |  | 31,500 |
|  | **Subtotal** |  |  |  | **54,195.00 birr** |
| 3 | **Training** | No. of participant | Payment /individual | No. of days |  |
|  | trainee | 20 | 300 | 5 | 30,000 |
|  | trainer | 1 | 300 | 5 | 1500 |
|  | **Subtotal** |  |  |  | **31,500.00 birr** |
| 4 | **Transport and communication** |  |  |  |  |
|  | **Transport** |  |  |  | 8000 |
|  | Communication |  |  |  | **5000** |
|  | **Subtotal** |  |  |  | **13,000** |
|  | Grand total | | | | **263,695.00 birr** |
|  | **Contingency (5%)** |  |  |  | **13,184.75** |
|  | **Sum Grand total** | | | | **276,879.75 birr** |

## 8.2 Budget summary

Table 6: budget summary for couple based violence prevention education in Hadiya zone, 2022-20223

|  | Budget category | **Total cost in ETB** |
| --- | --- | --- |
| 1 | Personal cost | **165,000.00birr** |
| 2 | Stationary | **54,195.00birr** |
| 4 | Transport and communication | **13000.00birr** |
| 5 | Contingency | **13,184.75** |
|  | Sum Grand total | **276,879.75 birr** |

Budget Source – Jimma and Wachemo University

# REFERENCES

ABAY WODAY TADESSE, NEGUSSIE DEYESS, ABIGIYA WONDIMAGEGNEHU, GEBEYAW BISET & MIHRET, S. 2018. <Intimate partner violence during pregnancy and preterm birth among mothers who gave birth in public hospitals, Amhara Region, Ethiopia: A case-control study>. *Ethiopian Journal of health develeopment,* 34.

ABDULAHI, M., FRETHEIM, A. & MAGNUS, J. H. 2018. Effect of breastfeeding education and support intervention (BFESI) versus routine care on timely initiation and exclusive breastfeeding in Southwest Ethiopia: study protocol for a cluster randomized controlled trial. *BMC Pediatr,* 18**,** 313.

ABRAMSKY, T., CHARLOTTE H WATTS, CLAUDIA GARCIA-MORENO, KAREN DEVRIES, LIGIA KISS, MARY ELLSBERG, HENRICA AFM JANSEN & HEISE, L. 2011. <What factors are associated with recent intimate partner violence? findings from the WHO multi-country study on women’s health and domestic violence>. *BMC public health* 11.

ADHENA, G., OLJIRA, L., DESSIE, Y. & HIDRU, H. D. 2020. Magnitude of Intimate Partner Violence and Associated Factors among Pregnant Women in Ethiopia. *Advances in Public Health,* 2020**,** 1-9.

ADHIA, A., GELAYE, B., FRIEDMAN, L. E., MARLOW, L. Y., MERCY, J. A. & WILLIAMS, M. A. 2019. Workplace interventions for intimate partner violence: A systematic review. *J Workplace Behav Health,* 34.

AFIAZ, A., BISWAS, R. K., SHAMMA, R. & ANANNA, N. 2020. Intimate partner violence (IPV) with miscarriages, stillbirths and abortions: Identifying vulnerable households for women in Bangladesh. *PLoS One,* 15**,** e0236670.

AGOSTINI, F., NERI, E., SALVATORI, P., DELLABARTOLA, S., BOZICEVIC, L. & MONTI, F. 2015. Antenatal depressive symptoms associated with specific life events and sources of social support among Italian women. *Matern Child Health J,* 19**,** 1131-41.

AHINKORAH, B. O. 2021. Polygyny and intimate partner violence in sub-Saharan Africa: Evidence from 16 cross-sectional demographic and health surveys. *SSM Popul Health,* 13**,** 100729.

AHMED, S., KOENIG, M. A. & STEPHENSON, R. 2006. Effects of domestic violence on perinatal and early-childhood mortality: evidence from north India. *Am J Public Health,* 96**,** 1423-8.

AL SHIDHANI, N. A., AL KENDI, A. A. & AL KIYUMI, M. H. 2020. Prevalence, Risk Factors and Effects of Domestic Violence Before and During Pregnancy on Birth Outcomes: An Observational Study of Literate Omani Women. *Int J Womens Health,* 12**,** 911-925.

ALEBEL, A., KIBRET, G. D., WAGNEW, F., TESEMA, C., FEREDE, A., PETRUCKA, P., BOBO, F. T., BIRHANU, M. Y., TADESSE, A. A. & ESHETIE, S. 2018. Intimate partner violence and associated factors among pregnant women in Ethiopia: a systematic review and meta-analysis. *Reprod Health,* 15**,** 196.

ALHUSEN, J. L., GELLER, R., DREISBACH, C., CONSTANTOULAKIS, L. & SIEGA-RIZ, A. M. 2017. Intimate Partner Violence and Gestational Weight Gain in a Population-Based Sample of Perinatal Women. *J Obstet Gynecol Neonatal Nurs,* 46**,** 390-402.

ALHUSEN, J. L., RAY, E., SHARPS, P. & BULLOCK, L. 2015. Intimate partner violence during pregnancy: maternal and neonatal outcomes. *J Womens Health (Larchmt),* 24**,** 100-6.

ANTAI, D. 2011. Controlling behavior, power relations within intimate relationships and intimate partner physical and sexual violence against women in Nigeria. *BMC Public Health* 11.

ASHENAFI, W., MENGISTIE, B., EGATA, G. & BERHANE, Y. 2020.

ASHENAFI, W., MENGISTIE, B., EGATA, G. & BERHANE, Y. 2021. <Prevalence and Associated Factors of Intimate Partner Violence During Pregnancy in Eastern Ethiopia>. *International Journal ofWomen's Health,* 12.

ASSEFA, N., BERHANE, Y. & WORKU, A. 2013. Pregnancy rates and pregnancy loss in Eastern Ethiopia. *Acta Obstet Gynecol Scand,* 92**,** 642-7.

ASSEFA, Y., GELAW, Y. A., HILL, P. S., TAYE, B. W. & VAN DAMME, W. 2019. Community health extension program of Ethiopia, 2003-2018: successes and challenges toward universal coverage for primary healthcare services. *Global Health,* 15**,** 24.

BELAY, S., ASTATKIE, A., EMMELIN, M. & HINDERAKER, S. G. 2019. Intimate partner violence and maternal depression during pregnancy: A community-based cross-sectional study in Ethiopia. *PLoS One,* 14**,** e0220003.

BERHANIE, E., GEBREGZIABHER, D., BERIHU, H., GEREZGIHER, A. & KIDANE, G. 2019. Intimate partner violence during pregnancy and adverse birth outcomes: a case-control study. *Reprod Health,* 16**,** 22.

BEYENE, G. M., AZALE, T., GELAYE, K. A. & AYELE, T. A. 2021. Depression remains a neglected public health problem among pregnant women in Northwest Ethiopia. *Arch Public Health,* 79**,** 132.

CHERNET, A. G. & CHERIE, K. T. 2020. Prevalence of intimate partner violence against women and associated factors in Ethiopia. *BMC Womens Health,* 20**,** 22.

DEVRIES, K. M., KISHOR, S., JOHNSON, H., STÖCKL, H., BACCHUS, L. J., GARCIA-MORENO, C. & WATTS, C. 2010. Intimate partner violence during pregnancy: analysis of prevalence data from 19 countries. *Reproductive Health Matters,* 18**,** 158-170.

DI NAPOLI, I., PROCENTESE, F., CARNEVALE, S., ESPOSITO, C. & ARCIDIACONO, C. 2019. Ending Intimate Partner Violence (IPV) and Locating Men at Stake: An Ecological Approach. *Int J Environ Res Public Health,* 16.

DORCAS E. BEATON, B., MSC, PHD,*†‡§ CLAIRE BOMBARDIER, MD, FRCP,*§⫼¶# FRANCIS GUILLEMIN, MD, MSC,** AND MARCOS BOSI FERRAZ, MD, MSC, PHD†† 2000. <Guidelines for the Process of Cross-Cultural Adaptation of Self-Report Measuresf>. *SPINE,* 25**,** 3186–3191.

DWORKIN, S. L. 2012. Sample size policy for qualitative studies using in-depth interviews. *Arch Sex Behav,* 41**,** 1319-20.

EL MORR, C. & LAYAL, M. 2020. Effectiveness of ICT-based intimate partner violence interventions: a systematic review. *BMC Public Health,* 20**,** 1372.

ETIENNE G. KRUG, LINDA L. DAHLBERG, JAMES A. MERCY, ZWI, A. B. & LOZANO, R. 2002. Worldreportonviolenceandhealth. Geneva, World Health Organization.

FEKADU DADI, A., MILLER, E. R. & MWANRI, L. 2020. Antenatal depression and its association with adverse birth outcomes in low and middle-income countries: A systematic review and meta-analysis. *PLoS One,* 15**,** e0227323.

FULU, E. & MIEDEMA, S. 2015. Violence Against Women: Globalizing the Integrated Ecological Model. *Violence Against Women,* 21**,** 1431-55.

GASHAW, B. T., MAGNUS, J. H., SCHEIB, B. & SOLBRAEKKEN, K. N. 2019. Community Stakeholders' Perspectives on Intimate Partner Violence during Pregnancy-A Qualitative Study from Ethiopia. *Int J Environ Res Public Health,* 16.

GEBRESLASIE, K. Z., WELDEMARIAM, S., GEBRE, G. & MEHARI, M. A. 2020. Intimate partner violence during pregnancy and risk of still birth in hospitals of Tigray region Ethiopia. *Ital J Pediatr,* 46**,** 107.

GEBREWAHD, G. T., GEBREMESKEL, G. G. & TADESSE, D. B. 2020. Intimate partner violence against reproductive age women during COVID-19 pandemic in northern Ethiopia 2020: a community-based cross-sectional study. *Reprod Health,* 17**,** 152.

GETINET, W., AMARE, T., BORU, B., SHUMET, S., WORKU, W. & AZALE, T. 2018. Prevalence and Risk Factors for Antenatal Depression in Ethiopia: Systematic Review. *Depress Res Treat,* 2018**,** 3649269.

HARVEY, S., ABRAMSKY, T., MSHANA, G., HANSEN, C. H., MTOLELA, G. J., MADAHA, F., HASHIM, R., KAPINGA, I., WATTS, C., LEES, S. & KAPIGA, S. 2021. A cluster randomised controlled trial to evaluate the impact of a gender transformative intervention on intimate partner violence against women in newly formed neighbourhood groups in Tanzania. *BMJ Glob Health,* 6.

HEALTH, F. D. R. O. E. M. O. 2010. <Ethiopia_HSDP_IV_Final_ 2010 -2015.pdf>.

JATTA, J. W. & OUEDRAOGO, J. C. R. P. 2021.

JILL RYAN & ROMAN, N. V. 2019. <Family-centered interventions for intimate partner violence: A systematic review>. *Journal of injury and violence prevention* 17.

K. M. DEVRIES, J. Y. T. MAK, C. GARCÍA-MORENO, M. PETZOLD, J. C. C., G. FALDER, S. L., L. J. BACCHUS, R. E. E., L. ROSENFELD, C. PALLITTO, T. VOS, N. ABRAHAMS & WATTS, C. H. 2013. Global prevalence of intimate partner violence against women *American Association for the Advancement of Science,* 340.

KIRANMAI DEVINENI, SHANTHA KUMARI, NAGAMANI SODUMU & GARG, R. 2018. Effects of Intimate Partner Violence on Pregnancy Outcome. *<Journal of South Asian Federation of Obstetrics and Gynaecologyf>*

10**,** 142-148.

LEIGHT, J. & WILSON, N. 2021. Intimate partner violence and maternal health services utilization: evidence from 36 National Household Surveys. *BMC Public Health,* 21**,** 405.

LENCHA, B., AMEYA, G., BARESA, G., MINDA, Z. & GANFURE, G. 2019. Intimate partner violence and its associated factors among pregnant women in Bale Zone, Southeast Ethiopia: A cross-sectional study. *PLoS One,* 14**,** e0214962.

LUHUMYO, L., MWALIKO, E., TONUI, P., GETANDA, A. & HANN, K. 2020. The magnitude of intimate partner violence during pregnancy in Eldoret, Kenya: exigency for policy action. *Health Policy Plan,* 35**,** i7-i18.

MARTIN-DE-LAS-HERAS, S., VELASCO, C., LUNA-DEL-CASTILLO, J. D. & KHAN, K. S. 2019. Maternal outcomes associated to psychological and physical intimate partner violence during pregnancy: A cohort study and multivariate analysis. *PLoS One,* 14**,** e0218255.

MCQUIGG, R. J. A. 2016. Domestic Violence as a Human Rights Issue:Rumor v. Italy. *European Journal of International Law,* 26**,** 1009-1025.

MEMIAH, P., BOND, T., OPANGA, Y., KINGORI, C., COOK, C., MWANGI, M., GITAHI-KAMAU, N., MUBANGIZI, D. & OWUOR, K. 2020. Neonatal, infant, and child mortality among women exposed to intimate partner violence in East Africa: a multi-country analysis. *BMC Womens Health,* 20**,** 10.

MULUNEH, M. D., STULZ, V., FRANCIS, L. & AGHO, K. 2020. Gender Based Violence against Women in Sub-Saharan Africa: A Systematic Review and Meta-Analysis of Cross-Sectional Studies. *Int J Environ Res Public Health,* 17.

MUSA, A., CHOJENTA, C. & LOXTON, D. 2020. High rate of partner violence during pregnancy in eastern Ethiopia: Findings from a facility-based study. *PLoS One,* 15**,** e0233907.

MUTISYA, R. K., NGURE, K. & MWACHARI, C. 2018. A psychosocial intervention to reduce gender-based violence and antepartum depressive symptoms in pregnant women in Kisumu County, Kenya: a quasi-experimental study. *The Pan African medical journal,* 29.

OCHE, O. M., ADAMU, H., ABUBAKAR, A., ALIYU, M. S. & DOGONDAJI, A. S. 2020. Intimate Partner Violence in Pregnancy: Knowledge and Experiences of Pregnant Women and Controlling Behavior of Male Partners in Sokoto, Northwest Nigeria. *International journal of reproductive medicine,* 2020**,** 1-10.

PUN, K. D., RISHAL, P., DARJ, E., INFANTI, J. J., SHRESTHA, S., LUKASSE, M. & SCHEI, B. 2019. Domestic violence and perinatal outcomes - a prospective cohort study from Nepal. *BMC Public Health,* 19**,** 671.

RIVARA, F. P., ANDERSON, M. L., FISHMAN, P., BONOMI, A. E., REID, R. J., CARRELL, D. & THOMPSON, R. S. 2007. Intimate partner violence and health care costs and utilization for children living in the home. *Pediatrics,* 120**,** 1270-7.

SARA COOLS & KOTSADAM, A. 2017. <Resources and Intimate Partner Violence in Sub-Saharan Africa>. *Elsevie,* 95**,** 211-230.

SEIDU, A. A., ABOAGYE, R. G., AHINKORAH, B. O., ADU, C. & YAYA, S. 2021. Intimate partner violence as a predictor of marital disruption in sub-Saharan Africa: A multilevel analysis of demographic and health surveys. *SSM Popul Health,* 15**,** 100877.

SHAMU, S., ABRAHAMS, N., TEMMERMAN, M., MUSEKIWA, A. & ZAROWSKY, C. 2011. A systematic review of African studies on intimate partner violence against pregnant women: prevalence and risk factors. *PLoS One,* 6**,** e17591.

SHARMA, B. 2016. A focus on reliability in developmental research through Cronbach’s Alpha among medical, dental and paramedical professionals. *Asian Pacific Journal of Health Sciences,* 3**,** 271-278.

SIGALLA, G. N., RASCH, V., GAMMELTOFT, T., MEYROWITSCH, D. W., ROGATHI, J., MANONGI, R. & MUSHI, D. 2017. Social support and intimate partner violence during pregnancy among women attending antenatal care in Moshi Municipality, Northern Tanzania. *BMC Public Health,* 17**,** 240.

SPERBER, A. D., DEVELLIS, R. F. & BOEHLECKE, B. 2016. Cross-Cultural Translation. *Journal of Cross-Cultural Psychology,* 25**,** 501-524.

TEMESGEN, T., TEJI, K., DHERESA, M. & ASEGID, A. 2019.

TESFA, A., DIDA, N., GIRMA, T. & ABOMA, M. 2020. Intimate Partner Violence, Its Sociocultural Practice, and Its Associated Factors Among Women in Central Ethiopia. *Risk Manag Healthc Policy,* 13**,** 2251-2259.

TROTT, C. D., HARMAN, J. J. & KAUFMAN, M. R. 2017. Women's Attitudes Toward Intimate Partner Violence in Ethiopia: The Role of Social Norms in the Interview Context. *Violence Against Women,* 23**,** 1016-1036.

TURNER, D. T., RIEDEL, E., KOBEISSI, L. H., KARYOTAKI, E., GARCIA-MORENO, C., SAY, L. & CUIJPERS, P. 2020. Psychosocial interventions for intimate partner violence in low and middle income countries: A meta-analysis of randomised controlled trials. *J Glob Health,* 10**,** 010409.

UN 1993. <General assembly on Declaration on the Elimination of Violence against

Womenp>.

UNDP 2015. <Sustainable Development Goals (SDGs)>.

UTHMAN, O. A., LAWOKO, S. & MORADI, T. 2009. Factors associated with attitudes towards intimate partner violence against women: a comparative analysis of 17 sub-Saharan countries. *BMC Int Health Hum Rights,* 9**,** 14.

WHO 2007. <WHO ethical and safety recommendations for researching, documenting and monitoring sexual violence in emergencies.>.

WHO 2010. <WHO Multi-country Study on Women's Health and Domestic Violence against Womeny .pdf>. *JSTOR,* 65.

WHO 2011a. <WHO Intimate partner violence during pregnancy information sheet.pdf>.

WHO. 2011b. <WHO intimate partner violence during pregnancy. an information sheet pdf>.

WILD, D., GROVE, A., MARTIN, M., EREMENCO, S., MCELROY, S., VERJEE-LORENZ, A., ERIKSON, P., TRANSLATION, I. T. F. F. & CULTURAL, A. 2005. Principles of Good Practice for the Translation and Cultural Adaptation Process for Patient-Reported Outcomes (PRO) Measures: report of the ISPOR Task Force for Translation and Cultural Adaptation. *Value Health,* 8**,** 94-104.

YOHANNES, K., ABEBE, L., KISI, T., DEMEKE, W., YIMER, S., FEYISO, M. & AYANO, G. 2019. The prevalence and predictors of domestic violence among pregnant women in Southeast Oromia, Ethiopia. *Reprod Health,* 16**,** 37.

ZHD, H. 2021. <Hadiya ZHD 2021MNCH-MH Annual performance Report .pdf>.

**ANNEXES**

**Annex I: Participants’ information sheet and Consent form/English/**

**Study Title**: ***Effect Couple based violence prevention education on intimate partner violence during pregnancy in Hadiya Zone, Southwest Ethiopia***

**Principal investigator: Zeleke Dutamo**

**Information sheet**

Good day! My name is----------------- I am working in the research project entitled “***effect Couple based violence prevention education on intimate partner violence during pregnancy in Hadiya Zone, Southwest Ethiopia’’.*** Study is being conducted in this health community by Mr. Zeleke Dutamo (MPH), who is studying for his PhD degree at Jimma University, institute of health. I kindly request you to lend me your attention to explain you about the study and being selected as the study participant. We are carrying out a study on women health and would appreciate your participation in the study. This form gives you information on the study procedures and your rights as a participant. Participation is voluntary. Now I will explain to you about this study and information so that you could make your choice and decision. This consent form may contain words that you do not understand. Please ask me to stop as we go through the information and I will take time to explain. If you have questions later, you can ask them of me or of another researcher.

**Purpose of the research**

The purpose of this study is to evaluate the effectiveness of couple based health-education on women’s health.

**Study Objective**: The objective of study is to assess the effectiveness of couples- based health education- on women’s health

**Study schedule and data collection**: The study involves six education sessions at health center, or health post found in the cluster. In addition, at the beginning, and after two months of delivery, an enumerator will collect informations

**Your Responsibility (couples):** You are expected to let researchers visit you at home, answer the questions asked by the research team. Additionally, you are also expected to attend about 40-45 minutes of discucation in each session provided by the research team.There will be an interview which may last 15 minutes before the health education and at the end-line.

**Confidentiality:** The information that you provide us will be confidential. There will be no information that will identify you and your organization/home /kebele. The findings of the study will be general for the study population and will not reflect anything particular of individual persons or housing. The questioner will be coded to exclude showing names; no references will be made in oral or written reports that could link participants to the research.

**Benefits**: The participants will benefit from free education on women’s health from our research team.

**Risks:** The study has no potential risks to the participants. No research-related injuries are anticipated. This is because no medications, chemicals or foods will be administered to the participants.

**Voluntarism**: Participation is voluntary and you can discontinue or decline to answer any questions, feel free to do so. We, however, encourage you to participate in the study as the findings will be important for designing programs for improving women’s health

**Payment**: You will not receive any payment for participating into the study. Similarly, you will not be charged any fee for participating into the study.

**CONTACTS**

Should you have any questions concerning the study that you feel are not addressed, you may enquire further from the **principal investigator** at the phone number- 0910489741/0930888897.

Do you have any opinion regarding this study? Do you agree to participate in this study?

Yes, continue No, thank you!

**Statement of Consent**

I have read/ (it was read) to me this consent form or read for me the participant information. I have clearly understood the purpose of the research, the procedure, risks and benefits, issues of confidentiality rights to or not to participate and contact address for any queries. I have given the opportunity to ask questions for things that may have been unclear. I was informed that I have the right to withdraw from the study at any time or not to answer any question that I do not want. Therefore, I declare my voluntary consent for to participate in this study with my signature or putting your left thumb print as indicated below.

Signature/ Thumb print of husband: ___________ Date: ________________________

Signature/ Thumb print of wife: ___________ Date: ___________________________

**Annex II: Structured Interviewer administered questionnaire for pregnant women (English version)**

Date of the interview (Eth. Calendar) ------------

Identification of interviewer-----------------

Questionnaire I.D----------------------

Interview kebele-----------------------

Interview start time-------------------

Interview end time-------------------

| **PART ONE:Socio-demographic charactersistics** | | | | | | | | | | | | | | | | | | | | | | |
| --- | --- | --- | --- | --- | --- | --- | --- | --- | --- | --- | --- | --- | --- | --- | --- | --- | --- | --- | --- | --- | --- | --- |
| S.No | Questions | | | Response | | | | | | | | | | | | | | | | | Skip to | |
| 101 | What is your age? | | | Enter complete age in years…………….… | | | | | | | | | | | | | | | | |  | |
| 102 | What was your age at marriage? | | | Enter complete age in years…………….… | | | | | | | | | | | | | | | | |  | |
| 103 | What is your place of residence? | | | Rural………………………………………….…1  Urban……………………………………………2 | | | | | | | | | | | | | | | | |  | |
| 104 | How long you have been in marriage? | | | Enter complete year/s…………………………… | | | | | | | | | | | | | | | | |  | |
| 105 | What is your husband’s age? | | | Enter complete age in year--------------------------- | | | | | | | | | | | | | | | | |  | |
| 106 | What is your religion? | | | Protestant.............................................................1  Orthodox..............................................................2  Muslim.................................................................3  Catholic ..............................................................4  Others, specify.....................................................5 | | | | | | | | | | | | | | | | |  | |
| 107 | What is your husband’s religion? | | | Protestant..............................................................1  Orthodox...............................................................2  Muslim..................................................................3  Catholic ...............................................................4  Others, specify.....................................................5 | | | | | | | | | | | | | | | | |  | |
| 108 | What is your ethnicity? | | | Hadiya..................................................................1  Kembata...............................................................2  Silte......................................................................3  Amhara.................................................................4  Others, specify.....................................................5 | | | | | | | | | | | | | | | | |  | |
| 109 | What is your husband’s ethnicity | | | Hadiya..................................................................1  Kembata...............................................................2  Silte......................................................................3  Amhara................................................................4  Others, specify.....................................................5 | | | | | | | | | | | | | | | | |  | |
| 110 | What is your education level? | | | No education.........................................................1  Attended elementary school(1-8).........................2  Attended high school(9-12)………………….….3  Attended college or higher(tertiary)education…..4 | | | | | | | | | | | | | | | | |  | |
| 111 | What is your husband’s education level? | | | No education.........................................................1  Attended elementary school(1-8).........................2  Attended high school(9-12)……………………..3  Attended college or higher(tertiary)education…..4 | | | | | | | | | | | | | | | | |  | |
| 112 | What is your occupation? | | | Housewife.............................................................1  Government employee..........................................2  Self-employed ……………………………..........3  Others, specify......................................................4 | | | | | | | | | | | | | | | | |  | |
| 113 | What is your husband’s occupation? | | | Farmer ..................................................................1  Government employee........................................2  Self-employed ……………………………..........3  Others, specify......................................................4 | | | | | | | | | | | | | | | | |  | |
| 114 | What is your average family monthly income in Birr? | | | Birr per month(estimate of the respondent)  ……………………………. | | | | | | | | | | | | | | | | |  | |
| 115 | Does your husband currently smoke cigarete? | | | Yes………………...………………………….....1  No………………...………………………….......2 | | | | | | | | | | | | | | | | |  | |
| 116 | Does your husband currently drink alcohol? | | | Yes………………...………………………….....1  No………………...………………………….......2 | | | | | | | | | | | | | | | | |  | |
| 117 | How many wives does your husband currently have? | | | One ……………...………………………...….....1  Two and above……………...……………...........2 | | | | | | | | | | | | | | | | |  | |
|  | **PART TWO: Household Variables** | | | | | | | | | | | | | | | | | | | |  | |
| 201 | What type of house does this household have? | | | | | Corrugated iron sheet house-----1  Grass roof house------------------2 | | | | | | | | | | | | | | |  | |
| 202 | Do you have separate room for sleeping? | | | | | 1.yes | | 2.No | | | | | | | |  | | | | | If no go to Q204 | |
| 203 | If yes for Q202, how many rooms? | | | | | 1.One | | 2. Two | | | | | | | | 3.Three | | | | |  | |
| 204 | Do you have a separate room for animals? | | | | | 1.yes | | 2.No | | | | | | | |  | | | | |  | |
| 205 | Do you have a separate room which is used as a kitchen? | | | | | 1.One | | 2. Two | | | | | | | |  | | | | |  | |
| 206 | Does any member of this household own cows? | | | | | 1. Yes | | 2. No | | | | | | | | If yes, how many? | | | | |  | |
| 207 | Does any member of this household own oxen? | | | | | 1. Yes | | 2. No | | | | | | | | If yes, how many? | | | | |  | |
| 208 | Does any member of this household own calves? | | | | | 1. Yes | | 2. No | | | | | | | | If yes, how many? | | | | |  | |
| 209 | Does any member of this household own other cattle? | | | | | 1. Yes | | 2. No | | | | | | | | If yes, how many? | | | | |  | |
| 210 | Does any member of this household own horses? | | | | | 1. Yes | | 2. No | | | | | | | | If yes, how many? | | | | |  | |
| 211 | Does any member of this household own donkeys? | | | | | 1. Yes | | 2. No | | | | | | | | If yes, how many? | | | | |  | |
| 212 | Does any member of this household own mules? | | | | | 1. Yes | | 2. No | | | | | | | | If yes, how many? | | | | |  | |
| 213 | Does any member of this household own sheeps? | | | | | 1. Yes | | 2. No | | | | | | | | If yes, how many? | | | | |  | |
| 214 | Does any member of this household own goats? | | | | | 1. Yes | | 2. No | | | | | | | | If yes, how many? | | | | |  | |
| 215 | Does any member of this household own chickens? | | | | | 1. Yes | | 2. No | | | | | | | | If yes, how many? | | | | |  | |
| 216 | Does any member of this household own beehives? | | | | | 1. Yes | | 2. No | | | | | | | | If yes, how many? | | | | |  | |
| 217 | Does any member of this household own any agricultural land? | | | | | 1. Yes | | 2. No | | | | | | | | If yes, how many hectare? | | | | |  | |
| 218 | Does any member of this household own Radio? | | | | | 1. Yes | | 2. No | | | | | | | | If yes, how many? | | | | |  | |
| 219 | Does any member of this household own Television? | | | | | 1. Yes | | 2. No | | | | | | | | If yes, how many? | | | | |  | |
| 220 | Does any member of this household own Telephone (Mobile)? | | | | | 1. Yes | | 2. No | | | | | | | | If yes, how many? | | | | |  | |
| 221 | Does any member of this household own table? | | | | | 1. Yes | | 2. No | | | | | | | | If yes, how many? | | | | |  | |
| 222 | Does any member of this household own chair? | | | | | 1. Yes | | 2. No | | | | | | | | If yes, how many? | | | | |  | |
| 223 | Does any member of this household own bed with cotton/sponge/spring mattress? | | | | | 1. Yes | | 2. No | | | | | | | | If yes, how many? | | | | |  | |
| 224 | Does any member of this household own a solar lamp? | | | | | 1. Yes | | 2. No | | | | | | | | If yes, how many? | | | | |  | |
| 225 | Does any member of this household own watch? | | | | | 1. Yes | | | | 2. No | | | | | | If yes, how many? | | | | |  | |
| 226 | Does any member of this household own bicycle? | | | | | 1. Yes | | | | 2. No | | | | | | If yes, how many? | | | | |  | |
| 227 | Does any member of this household own motor? | | | | | 1. Yes | | | | 2. No | | | | | | If yes, how many? | | | | |  | |
| 228 | Does any member of this household own animal-drawn cart? | | | | | 1. Yes | | | | 2. No | | | | | | If yes, how many? | | | | |  | |
| 229 | Does any member of this household have a bank account? | | | | | 1. Yes | | | | 2. No | | | | | | If yes, how many? | | | | |  | |
| 230 | Does any member of this household own khat farm? | | | | | 1.Yes | | | | 2.No | | | | | | If yes, how many hectare? | | | | |  | |
| 231 | Does any member of this household own eucalyptus farm? | | | | | 1.Yes | | | | 2. No | | | | | | If yes, how many hectare? | | | | |  | |
| 232 | How many quintals of the following cereals do the family produced in this year? | | | | | 1.Teff-- | | | | 3.Maize--------- | | | | | | 5. Barley  ---------- | | | | |  | |
|  |  |  |  |  |  | 2.Millet ----- | | | | 4. Wheat ……. | | | | | | 6. others, Specify | | | | |  | |
| 233 | How many quintals of the following legume do the family produced in this year? | | | | | 1.Bean  --------  2.pea  -------- | | | | 3. Lentil  -------  4.Nut  ------- | | | | | | 5. Chick pea  --------  6.grass pea  -----------  7.others,pecify | | | | |  | |
| 234 | How many kilograms of vegetables do the family produced? | | | | | 1.Gomen  2.Carrot  3.Potato | | | | 4. Tomato  5. Cabbage  6. Onion  10. Garlic | | | | | | 7.Beat root  8. Pumpkin  9.Pepper  11.Other specify | | | | |  | |
| 235 | How many kilograms of fruits do the family produced? | | | | | 1.Mango 2.Banana  3.Avocado | | | | 4. Papaya  5. Guava  6.Lemon | | | | | | 7. Other specify- | | | | |  | |
|  | **PART THREE: History of recent pregnancy and delivery** | | | | | | | | | | | | | | | | | | | | | |
| 301 | How many live births have you had? | | Enter number……………………………….. | | | | | | | | | | | | | | | | |  | | |
| 302 | Did you attend ANC for your recent pregnancy from skilled health personnel (nurse, mid-wife, and doctor or health officer)? | | Yes………………………….……………..….1  No……………………………………………..2 | | | | | | | | | | | | | | | | | If ‘**no**’  Skip to  **306** | | |
| 303 | If yes, where did you attend? | | Hospital..............................................................1  Health center…..................................................2  Health post/ clinic..............................................3  Home..................................................................4 | | | | | | | | | | | | | | | | |  | | |
| 304 | How many months pregnant were you when you first received antenatal care for the recent pregnancy? | | Enter the number months/weeks……………… | | | | | | | | | | | | | | | | |  | | |
| 305 | Has your baby’s father attended any antinatal visits with you for the most recent pregnancy? | | Yes……………………………….……………1  No………………….………………………….2 | | | | | | | | | | | | | | | | |  | | |
| 306 | Where did you deliver your recent baby? | | Hospital...........................................................1  Health center……….………………………….2  Health post/clinic……..……………………….3  Home…………….…………………………….4 | | | | | | | | | | | | | | | | |  | | |
| 307 | If it is at hospital or health center or health post/clinic, was the husband/partner present during labor or delivery? | | Yes ….………………………………………...1  No …………………………….………………2 | | | | | | | | | | | | | | | | |  | | |
|  | **PART FOUR: HISTORY OF CURRENT PREGNANCY** | | | | | | | | | | | | | | | | | | |  | | |
| 401 | At the time you became pregnant, did you want to become pregnant then, did you want to wait until later, or did you not want to have any (more) children at all in current pregnancy ? | | want to become pregnant……………...……...1  want to wait until later…………………….…..2  did not want to have any (more) children at all.3 | | | | | | | | | | | | | | | | |  | | |
| 402 | Did you attend ANC for your current pregnancy from skilled health personnel (nurse, mid-wife, and doctor or health officer)? | | Yes…………………………………………….1  No ………………………………………...…..2 | | | | | | | | | | | | | | | | | If ‘**No**’ skip to  405 | | |
| 403 | If yes, where did you attend? | | Hospital..............................................................1  Health center…..................................................2  Health post/ clinic..............................................3  Home..................................................................4 | | | | | | | | | | | | | | | | |  | | |
| 404 | Has your baby’s father attended any ante-natal visits with you for the current pregnancy? | | Yes…………………………………………….1  No …………………………………………….2 | | | | | | | | | | | | | | | | |  | | |
| 405 | During current pregnancy, Did you notice signs of danger during pregnancy? | | Yes…………………………………………….1  No …………………………………………….2 | | | | | | | | | | | | | | | | |  | | |
| 406 | What kind of health problems can endanger when you were at current pregnancy?  **More than one could be ticked**)  (**Do not read the responses**) | | Yes No  Severe vaginal bleeding  during pregnancy..................1 2  Swollen hands/face...............1 2  Severe headache...................1 2  Absent fetal movement.........1 2  Blurred vision........................1 2  Severe vaginal bleeding  during labor and child birth…1 2  Pro-longed labor (>12 hours)...1 2  Convulsions.............................1 2  Severe bleeding following  Childbirth................................1 2  Loss of consciousness………...1 2 | | | | | | | | | | | | | | | | |  | | |
| 407 | What is the sex of child prior to this pregnancy? | | Male………………………………………….1  Female………………………………………..2 | | | | | | | | | | | | | | | | |  | | |
|  | **PART FIVE : WOMEN’S AUTONOMY, SOCIAL SUPPORT AND SEL-EFFICACY** | | | | | | | | | | | | | | | | | | |  | | |
| 500 | **WOMEN’S AUTONOMY** | | | | | | | | | | | | | | | | | | |  | | |
|  | Who makes Final decisions in your household activities :  1. wife alone  2. Respondent and husband together  3. Husband alone  4. someone else | | | |  | | | | | | | | | | | | | | |  | | |
| 501 | On household purchases including small and large ones? | | | | 1 | | | | 2 | | | | | 3 | | | | 4 | |  | | |
| 502 | Visiting relatives and friends? | | | | 1 | | | | 2 | | | | | 3 | | | | 4 | |  | | |
| 503 | Spending the wife's earnings? | | | | 1 | | | | 2 | | | | | 3 | | | | 4 | |  | | |
| 504 | The number of children to have? | | | | 1 | | | | 2 | | | | | 3 | | | | 4 | |  | | |
| 505 | Obtaining health care for you? | | | | 1 | | | | 2 | | | | | 3 | | | | 4 | |  | | |
| 506 | **SOCIAL SUPPORT** | | | | | | | | | | | | | | | | | | |  | | |
| 507 | How many people are so close to you that you can count on them if you have great personal problems? | | | | None...................................................1  1–2.....................................................2  3–5.....................................................3  5+......................................................4 | | | | | | | | | | | | | | |  | | |
| 508 | How much interest and concern do people show in what you do? | | | | none…………………………………1  little………………………..………...2  uncertain…………….........…………3  some………………….……………..4  a lot………………….………………5 | | | | | | | | | | | | | | |  | | |
| 509 | How easy is it to get practical help from neighbors if you should need it? | | | | very difficult …...………………..…1  difficult…………..…………………2  possible…………………..…………3  Easy…………………….………..…4  very easy…..….…………….………5 | | | | | | | | | | | | | | |  | | |
|  | **PART SIX: KNOWLEDGE, ATTITUDE AND CONTROLLING BEHAVIOUR QUESTIONS** | | | | | | | | | | | | | | | | | | |  | | |
| 600 | **Knowledge** | | | | | | | | | | | | | | | | | | |  | | |
| 601 | Have you heard of Intimate partner violence? | | | | 1. Yes 2. No | | | | | | | | | | | | | | |  | | |
| 602 | Media, hospitals, health center are examples of sources of information for intimate partner violence | | | | Yes ----------------------------------1  No -----------------------------------2 | | | | | | | | | | | | | | |  | | |
| 603 | IPV is a serious public health issue | | | | Yes ………………………………….1  No …………………………………..2 | | | | | | | | | | | | | | |  | | |
| 604 | Slapping, kicking, dragging, beating, choking, pushing, etc., are examples of IPV | | | | Yes…………………………………..1  No …………………………………..2 | | | | | | | | | | | | | | |  | | |
| 605 | Forcing partner to have sex when he/she does not want to is sexual violence | | | | Yes………………………………….1  No…………………………………..2 | | | | | | | | | | | | | | |  | | |
| 606 | Forcing partner to do something sexual that he/she finds degrading or humiliating is sexual violence | | | | Yes………………………………….1  No………………………..………….2 | | | | | | | | | | | | | | |  | | |
| 607 | Belittling or humiliating a partner in front of other people is psychological violence | | | | Yes……………………………….….1  No…………………………………...2 | | | | | | | | | | | | | | |  | | |
| 608 | Intimate partner violence during pregnancy has adverse maternal and newborn outcomes | | | | Yes…………………………….…….1  No…………………………………...2 | | | | | | | | | | | | | | |  | | |
| 609 | Intimate partner violence is human right violation | | | | Yes……………………………….….1  No…………………………………...2 | | | | | | | | | | | | | | |  | | |
| 610 | **Attitude** | | | | | | | | | | | | | | | | | | |  | | |
| 611 | In your opinion, is a husband justified in hitting or beating his wife in the following situation   1. Yes 2. No | | | | | |  | | | | | | | | | | | | |  | | |
| 612 | If she goes out without telling him? | | | | | | 1 | | | | | | | | | | 2 | | |  | | |
| 613 | If she neglects the children? | | | | | | 1 | | | | | | | | | | 2 | | |  |  |  |
| 614 | If she argues with him? | | | | | | 1 | | | | | | | | | | 2 | | |  |  |  |
| 615 | If she refuses to have sex with him? | | | | | | 1 | | | | | | | | | | 2 | | |  |  |  |
| 616 | If she burns the food? | | | | | | 1 | | | | | | | | | | 2 | | |  |  |  |
| 617 | **Controlling behavior** | | | | | | | | | | | | | | | | | | |  |  |  |
|  | In your opinion, is the husband is justified in controlling you in the following situation?   1. Yes 2. No | | | | | | | | | | | | | | | | | | |  |  |  |
| 618 | Jealous if she talked with other men | | | | | | 1 | | | | | | 2 | | | | | | |  | | |
| 619 | Accused her of unfaithfulness | | | | | | 1 | | | | | | 2 | | | | | | |  |  |  |
| 620 | Did not permit her to meet her friends | | | | | | 1 | | | | | | 2 | | | | | | |  | | |
| 621 | Tried to limit her contact with family | | | | | | 1 | | | | | | 2 | | | | | | |  |  |  |
| 622 | insisted on knowing where she is | | | | | | 1 | | | | | 2 | | | | | | | |  | | |
| 623 | did not trust her with money | | | | | | 1 | | | | | 2 | | | | | | | |  |  |  |
| 700 | **SELF-EFFICACY**  This questionnaire is a series of statements about your personal attitudes and traits.  Each statement represents a commonly held belief. Read each statement and decide to what  extent it describes you. There are no right or wrong answers. You will probably agree with some  of the statements and disagree with others, Please indicate your own personal beliefs about each  statement below by marking the letter that best describes your attitude or feeling. Please be  truthful and describe yourself as you really are, not as you would like to be.  This questionnaire is a series of statements about your personal attitudes and traits.  Each statement represents a commonly held belief. Read each statement and decide to what  extent it describes you. There are no right or wrong answers. You will probably agree with some  of the statements and disagree with others, Please indicate your own personal beliefs about each  statement below by marking the letter that best describes your attitude or feeling. Please be  truthful and describe yourself as you really are, not as you would like to be.  This questionnaire is a series of statements about your personal attitudes and traits. Please indicate your own personal beliefs about each statement when the interviewer reads that best describes your attitude or feeling   1. Not at all true 2. Hardly true 3. Moderately true 4. Exactly true | | | | | | Encircle the choice | | | | | | | | | | | | |  | | |
| 701 | I can always manage to solve difficult problems if I try hard enough | | | | | | 1 | | | | 2 | | | | 3 | | | | 4 |  | | |
| 702 | If someone opposes me, I can find the means and ways to get what I want. | | | | | | 1 | | | | 2 | | | | 3 | | | | 4 |  | | |
| 703 | It is easy for me to stick to my aims and accomplish my goals. | | | | | | 1 | | | | 2 | | | | 3 | | | | 4 |  | | |
| 704 | I am confident that I could deal efficiently with unexpected events | | | | | | 1 | | | | 2 | | | | 3 | | | | 4 |  | | |
| 705 | Thanks to my resourcefulness, I know how to handle unforeseen situations | | | | | | 1 | | | | 2 | | | | 3 | | | | 4 |  | | |
| 706 | I can solve most problems if I invest the necessary effort. | | | | | | 1 | | | | 2 | | | | 3 | | | | 4 |  | | |
| 707 | I can remain calm when facing difficulties because I can rely on my coping abilities | | | | | | 1 | | | | 2 | | | | 3 | | | | 4 |  | | |
| 708 | When I am confronted with a problem, I can usually find several solutions. | | | | | | 1 | | | | 2 | | | | 3 | | | | 4 |  | | |
| 709 | If I am in trouble, I can usually think of a solution | | | | | | 1 | | | | 2 | | | | 3 | | | | 4 |  | | |
| 710 | I can usually handle whatever comes my way. | | | | | | 1 | | | | 2 | | | | 3 | | | | 4 |  | | |
|  | **PART SEVEN: experience of intimate partner violence in the recent pregnancy(last pregnancy)** | | | | | | | | | | | | | | | | | | | | |  |
| 801 | **Physical** | | | | | | | | | | | | | | | | | | |  | |  |
|  | During recent pregnancy, did your partner: | Slap you or throw something at you Yes No  that could hurt you? ………………………………1 2  Push you, shove you or pull your hair……………..1 2  Hit you with his fist or with something  else that could hurt you?..........................................1 2  Kick you, drag you about or beat you up?...............1 2  Attempt to choke you or burn you on purpose?.......1 2  Threaten to use or actually use a gun,  Knife or other weapon against you? …..…………1 2 | | | | | | | | | | | | | | | | | |  | |  |
| 802 | **Psychological** | Yes No  Say or do something to humiliate  you in front of another person?....................1 2  Threaten to hurt or harm you or  someone you care about? ………………...1 2  Insult you or make you feel bad about yourself?...1 2  Do things to scare or intimidate you  on purpose (e.g., by the way he looked at you,  by yelling and smashing things)?...................1 2 | | | | | | | | | | | | | | | | | |  | |  |
|  | During your most recent pregnancy, did your partner: |  |  |  |  |  |  |  |  |  |  |  |  |  |  |  |  |  |  |  |  |  |
| 803 | **Sexual** |  | | | | | | | | | | | | | | | | | |  | |  |
|  | During your recent pregnancy, did your partner | Yes No  Physically force you to have sexual  intercourse with him when you did not want to?.....1 2  Force you with threats or in any other way  to perform sexual acts you did not want?.............1 2  Physically force you to perform any  other sexual acts you did not want to? …………....1 2 | | | | | | | | | | | | | | | | | |  | |  |
| 804 | If a woman is yes for 801 or 802 or 803, what did you do? | Nothing……………………………………………………..…..1  Reported to him relations…………………………………….....2  Quit relationship……………………………………………...…3 | | | | | | | | | | | | | | | | | | If ‘nothing’ skip to  805 | |  |
| 805 | **If nothing for 804, why?** | Yes No  Uncertainty that the situation is abusive…………....1 2  Fear………………………………………………….1 2  I forgave him…………………………………… ....1 2  Shame and embarrassment………………………..,,..1 2 | | | | | | | | | | | | | | | | | |  | |  |

Thank you!

**Annex III: Structured interviewer admininistrative questionnaire for husbands (English-Version)**

Date of the interview (Eth. Calendar) --------------

Identification of interviewer-----------------

Questionnaire I.D----------------------

Interview kebele----------------------

Interview start time-------------------

Interview end time-------------------

| **PART ONE: Background characteristics** | | | | | | | | |  |
| --- | --- | --- | --- | --- | --- | --- | --- | --- | --- |
| S.No | Questions | | Response | | | | | Skip to |  |
| 101 | What is your age? | | Enter complete age in years---------------- | | | | |  |  |
| 102 | What is your wife’s age? | | Enter complete age in years---------------- | | | | |  |  |
| 103 | What is your place of residence? | | Rural………………………………………….…1  Urban……………………………………………2 | | | | |  |  |
| 104 | How long you have been in marriage? | | Enter complete year/s………………………… | | | | |  |  |
| 105 | What is your religion? | | Protestant.............................................................1  Orthodox..............................................................2  Muslim.................................................................3  Catholic ..............................................................4  Others, specify.....................................................5 | | | | |  |  |
| 106 | What is your wife’s religion? | | Protestant..............................................................1  Orthodox...............................................................2  Muslim.................................................................3  Catholic ...............................................................4  Others, specify.....................................................5 | | | | |  |  |
| 107 | What is your ethnicity? | | Hadiya..................................................................1  Kembata...............................................................2  Silte......................................................................3  Amhara.................................................................4  Others, specify.....................................................5 | | | | |  |  |
| 108 | What is your wife’s ethnicity | | Hadiya..................................................................1  Kembata...............................................................2  Silte......................................................................3  Amhara................................................................4  Others, specify.....................................................5 | | | | |  |  |
| 109 | What is your education level? | | No education.........................................................1  Attended elementary school(1-8).........................2  Attended junior or senior high school(9-12)…….3  Attended college or higher(tertiary)education…..4 | | | | |  |  |
| 110 | What is your wife’s education level? | | No education......................................................1  Attended elementary school(1-8).......................2  Attended junior or senior high school(9-12)…..3  Attended college or higher(tertiary)education...4 | | | | |  |  |
| 111 | What is your occupation? | | Farmer .................................................................1  Government employee.........................................2  Self-employed …………………………….........3  Others, specify......................................................4 | | | | |  |  |
| 112 | What is your wife’s occupation? | | Housewife.............................................................1  Government employee.........................................2  Self-employed …………………………….........3  Others, specify......................................................4 | | | | |  |  |
| 113 | What is your average family monthly income in Birr? | | Birr per month(estimate of the respondent)  ………………………………… | | | | |  |  |
| 114 | How many wives do you have? | | One ……………...……………………...….....1  Two and above……………...…………..........2 | | | | |  |  |
|  | **PART TWO: KNOWLEDGE, ATTITUDE AND CONTROLLING BEHAVIOUR QUESTIONS** | | | | | | |  | |
| 200 | **Knowledge** | | | | | | |  | |
| 201 | Have you heard of Intimate partner violence? | | | Yes----------------------------1  No-----------------------------2 | | | |  | |
| 202 | Media, hospitals, health center are examples of sources of information for intimate partner violence | | | Yes ………………………………….1  No …………………………………..2 | | | | 0 | |
| 203 | IPVp is a serious public health issue | | | Yes ………………………………….1  No …………………………………..2 | | | |  | |
| 204 | Slapping, kicking, dragging, beating, choking, pushing, etc., are examples of IPV | | | Yes…………………………………..1  No …………………………………..2 | | | |  | |
| 205 | Forcing partner to have sex when he/she does not want to is sexual violence | | | Yes………………………………….1  No…………………………………..2 | | | |  | |
| 206 | Forcing partner to do something sexual that he/she finds degrading or humiliating is sexual violence | | | Yes………………………………….1  No………………………..………….2 | | | |  | |
| 207 | Belittling or humiliating a partner in front of other people is psychological violence | | | Yes……………………………….….1  No…………………………………...2 | | | |  | |
| 208 | Restricting a partner from contact with family or friends | | | Yes…………………………….…….1  No…………………………………...2 | | | |  | |
| 209 | Intimate partner violence is human right violation | | | Yes……………………………….….1  No…………………………………...2 | | | |  | |
| 210 | **Attitude** | | | | | | |  | |
|  | In your opinion, is a husband justified in hitting or beating his wife in the following situation?  1---------------Yes  2…………… No | | | | | | |  | |
| 211 | If she goes out without telling him? | Yes…………………………………….….…...1  No…………………………………….…….…2 | | | | | |  | |
| 212 | If she goes out without telling him? | Yes…………………………………….….…...1  No…………………………………….…….…2 | | | | | |  | |
| 213 | If she neglects the children? | Yes…………………………………….….…...1  No…………………………………….…….…2 | | | | | |  | |
| 214 | If she argues with him? | Yes…………………………………….….…...1  No…………………………………….…….…2 | | | | | |  | |
| 215 | If she refuses to have sex with him? | Yes…………………………………….….…...1  No…………………………………….…….…2 | | | | | |  | |
| 216 | **Controlling behavior** | | | | | | |  | |
|  | In your opinion, is the husband is justified in controlling his wife in the following  Situation? | | | | | |  | | |
| 217 | Jealous if she talked with other men | | | | 1 | 2 | |  | |
| 218 | Accused her of unfaithfulness | | | | 1 | 2 | |  |  |
| 219 | Did not permit her to meet her friends | | | | 1 | 2 | |  |  |
| 220 | Tried to limit her contact with family | | | | 1 | 2 | |  |  |
| 221 | insisted on knowing where she is | | | | 1 | 2 | |  |  |
| 222 | did not trust her with money | | | | 1 | 2 | |  |  |
|  | **PART THREE: Male Involvement in maternal health care (MHC)** | | | | | | |  | |
| 301 | Did your wife take ANC during her recent pregnancy at health facility? | Yes………………………………………..…...1  No……………………………………….….…2 | | | | | |  | |
| 302 | How long did it take to reach the nearest health institution? | Time in hour……………………………… | | | | | |  | |
| 303 | Have you ever accompanied her to ANC at least once during her last pregnancy? | Yes…………………………….………..…....1  No……………………………….………....…2 | | | | | |  | |
| 304 | If the answer is No why did you not accompy? (**Do not read the alternatives. More than**  **one response is possible**) | Feeling a women’s duty……………………...1  Lack of time to go………………………….…2  Lack of knowledge…………………….……..3  Social stigma………………………….………4  Shyness/embarrassment……………………....5  Fear of HIV test……………………………....6  Non-invitation………………………….……..7  Other specify……………………………….…8 | | | | | |  | |
| 305 | Where did your wife give birth for the recent baby? | Health center ---------------------------------------1  Hospital ---------------------------------------------2  Clinic-------------------------------------------------3  Home------------------------------------------------4 | | | | | |  | |
| 306 | If health institution (hospital or health center or clinic), were you present during labor or delivery? | Yes -------------------------------------------------1  No --------------------------------------------------2 | | | | | |  | |
| 307 | After your wife gave birth to (NAME) did any nurse, midwife or doctor or health officer check on her health? | Yes --------------------------------------------------1  No ---------------------------------------------------2 | | | | | |  | |
| 308 | If Yes for Q, did you accompany during the check-up? | Yes ---------------------------------------------------1  No----------------------------------------------------2 | | | | | |  | |

**Appendix-iv: CBVP-Education manual to trainers**

**Introduction**

Couples education package is a set of preventive messages designed to help foster a process of change to reduce intimate partner violence during pregnancy amongst couples in Hadiya Zone, Southwest, and Ethiopia. The contents included in the education manual to prevent intimate partner violence in pregnancy includes the following

- Gender-based violence
- Violence against women
- Intimate partner violence
- Types of intimate partner violence (physical, sexual, and psychological or emotional violence)
- Magnitude of intimate partner violence in pregnancy
- Inequitable gender norms and consequences
- The relationship between intimate partner violence and power and control
- Common triggers of IPV in pregnancy
- Uhhealthy and healthy relationship
- Common triggers of IPV and ways to challenge the triggers
- Effective communication and conflict resolution in relationship

**Structure of the sessions**

There are 6 total sessions in the anual. Each session is a maximum of 60 minutes

**Couples take- home exercises and reflections**

All sessions contain take-home exercises that are assigned at the end of the session, and to help the couples reflect upon and apply new ideas in their own lives. Research shows that doing exercises at home following training significantly increases the effectiveness of the program. Each session, likewise, starts with a reflection of the take-Home exercise. The format of this reflection is the same for each session, with different guiding questions to facilitate debriefs.

**Participants**

The manual will be implemented with couples in selected clusters of intervention. The education takes place at health center or health post in group.

**Supplies needed**

Simple training supplies including, parker, flip chart paper and poster are needed for the training. Sessions are designed with the understanding that they will be implemented in an enclosed workshop space. However, accommodations can be made if that is not available. It is recommended to arrange chairs in a semi-circle to help build connections and discussion between couples and HEW educator.

**Language**

The Amharic or Hadiyisa (local language) will be used in this training manual according to the participants’ preference.

**The training manual objective**

1. To enable the participants describe the GBV, VAW, IPV, IPV, types of IPV and Magnitude of IPV
2. To enable the couples to describe the extent of intimate partner violence
3. To enable the couples to explain adverse consequences of intimate partner violence during pregnancy
4. To enable couples to describe common triggers of violence and state the common ways to challenge the triggers of IPV
5. To enable the couples to describe inequitable gender norms related to IPV during pregnancy
6. To enable the couples to describe an effective communication skills and nonviolent ways of conflict resolution

The CBVPE session schedules, titles and objectives

| Session schedule by Gestational ages | Session title | Session objectives |
| --- | --- | --- |
| 1^st^ session  (13-16 weeks) | Program introduction & understanding GBV, VAW and IPV | To describe the goal of the program  To define GBV, VAW and IPV  To explain types and magnitude of IPV |
| 2^nd^ session  (17-20 weeks) | Consequences and common triggers of IPV during pregnancy | To describe adverse maternal and new-born consequences of IPV during pregnancy  To explain economic and social consequences of IPV during pregnancy  To identify common triggers of IPV  To state the ways to challenge triggers of IPV |
| 3^rd^ session  (21-24 weeks) | Inequitable gender norms related to IPV during pregnancy | To describe common gender norms for women and men  To differentiate gender and sex  To understand how inequality gender norms contribute to IPV during pregnancy  To describe the ways to challenge and transform inequitable gender norms |
| 4^th^ session  (25-28 weeks) | Unhealthy and healthy relationship | To differentiate healthy and unhealthy behaviors in marital relationship  To state important characteristics of healthy relationships |
| 5^th^ session  (29-32 weeks) | Power and control in relationship &  Joint decision making | To define power and control  To describe healthy power balance within relationship  To explain benefits of joint decision making  To describe and apply seven strategies to joint decision making |
| 6^th^ session  (33-36 weeks) | Communication & conflict resolution | To describe effective communication skills in relationship  To explain nonviolent ways of conflict resolution in relationship |

**Contents of the session and time allocated**

| **Activity** | **Allocated time** |
| --- | --- |
| **Session 1( 13-16 weeks of gestational age)** | **60 minutes.** |
| - Introducing each other | 10 minutes |
| - Gender based violence (what is it) - Violence against women(what is it) - Intimate partner violence(what is it ) - Types of intimate partner violence - Physical, sexual and psychological - Magnitude of IPV - Take home execises | 40 minutes |
| Summarization and appointment to next session | 10 minutes |
| **Session 2 (17-20 weeks of gestational age)** | **60 minutes** |
| - Recap and reflection on take home exercise | 10 minutes |
| - Adverse maternal and new-born consequences of IPV during pregnancy - Economic and social consequences of IPV during pregnancy - common triggers of IPV - The ways to challenge triggers of IPV | 40 minutes |
| - Introduction on take-home exercise and appointment to next session | 10 minutes |
| **Session 3 (21-24 weeks of gestational age)** | 60 minutes |
| - Recap and reflection on take home exercise | 10 minutes |
| - Common gender norms for women and men - Gender and sex - Contribution of inequality gender norms to IPV during pregnancy - Ways to challenge and transform inequitable gender norms | 40 minutes |
| - Introduction on take-home exercise and appointment to next session | 10 minutes |
| **Session 4 (25-28 weeks of gestational age)** | 55 |
| - Recap and reflection on take home exercises | 10 minutes |
| - Healthy and unhealthy behaviors in marital relationship - Important characteristics of healthy relationships | 35 minutes |
| - Introduction on take-home exercise and appointment to next session | 10 minutes |
| **Session 5 (29-32 weeks of gestational age)** | 55 minutes |
| - Recap and reflection on take home exercises | 10 minutes |
| - Power and control in relationship - Healthy power balance in relationship of couples - Benefits of joint decision making - Strategies to joint decision making | 35 minutes |
| - Introduction on take-home exercise and appointment to next session | 10 minutes |
| **Session 6(33-36 weeks of gestational age)** | **60** |
| - Effective communication skills in relationship | 10 minutes |
| - Nonviolent ways of conflict resolution in relationship | 35 |
| - Acknowledgement and close up | 15 minutes |

**Session 1: Introduction to Gender Based Violence (GBV), Violence against Women (VAW) and Intimate partner violence (IPV)**

**Session one will be conducted first day or contact**

**Suggested time: 60 minutes**

**Session objective**

- To enable the couples to define a GBV, VAM and IPV
- To describe the types of IPV in pregnancy
- To explain the magnitude of IPV during pregnancy

**Activity**

**Steps**

1. Getting to know the HEW educator and couples on

2. Warmly welcome the couples to the first education session.

3. Introduce the process: today we will begin introduction to GBV, VAW and IPV. I will be here to guide the process. However, your participation will propel us forward. Each of us brings something unique to this space—our character, our ideas, and our experiences. Your contributions are what will enrich this process and give it meaning in your lives.

4. Prepare the materials like note book, pen and poster.

5. Start the teaching on the given topic

- What is GBV?
- What is VAW?
- What is IPV?
- Types of IPV
- Magnitude of IPV

6. Give time for questions or ask questions whether the couples understand or not

Make sure that the couples responded at least 75 percent of the questions asked

If the couples do not respond at least 75 percent of questions raised by the trainer, repeat the session for another time.

7. Give take home exercise, appoint for the next session and acknowledge the couples for their participation.

**Session 2: Consequences and common triggers of IPV during pregnancy**

1. **Session 2 is provided in the second contact**
2. **Suggested time: 60 minutes**
3. **Session objective**

- To describe adverse maternal and new-born consequences of IPV during pregnancy
- To explain economic and social consequences of IPV during pregnancy
- To identify common triggers of IPV
- To state the ways to challenge triggers of IPV

1. **Activity**

**Steps**

1. Warmly welcome the couples to the 2^nd^ education session
2. Reflections on take home exercises with the couples
3. Introduce the session: today we will begin on maternal, social and economic consequences of IPV and common triggers of IPV will be here to guide the lecture. However, your participation will also propel us forward. Your contributions are what will enrich this session and give it meaning in your lives.
4. Prepare materials like note book and pen
5. Start the teaching on the given topic
6. Give time for questions or ask questions whether the couples understand or not

- Make sure that the couples responded at least 75 percent of the questions asked
- If the couples do not respond at least 75 percent of questions raised by the trainer, repeat the session for another time.

1. Introduce the take home exercise, appointment for next session and acknowledge couples for the their participation

**Session 3:**

**Provide the session three at third contact**

1. **Suggested time: 45 minutes**
2. **Session objectives**

- To enable the couples on pregnancy related consequences of violence during pregnancy

1. **Activity**

**Steps**

1. Warmly welcome the couples to the 3^rd^ education session
2. Reflections on take home exercises with the couples
3. Introduce the session: today we will continue on pregnancy-related consequences of violence during pregnancy. Furthermore, your participation has a paramount role in the success of this session.
4. Prepare materials like note book, pen and posters
5. Start the teaching on the given topic

- Pregnancy related consequences of violence

Direct and indirect effect

- Effect of violence on perpetrators
- Effect of violence on society

1. Importance of male involvement in maternal health care
2. Give time for questions or ask questions whether the couples understand or not

- Make sure that the couples responded at least 75 percent of the questions asked
- If the couples do not respond at least 75 percent of questions raised by the trainer, repeat the session for another time.

1. Introduce the take home exercise and appointment for next session, acknowledge the participants

**Session4: power, control and intimate partner violence**

1. **Provide this session at fourth contact**
2. **Suggested time: 45 minutes.**
3. **Session objectives:**

- To enable the couples with power and control balance in relationship
- To enable the couples with a healthy power balance in relationship of couples
- To discuss the role of male partners during pregnancy

1. **Activities**

**Steps**

1. Warmly welcome the couples to the 4^th^ education session
2. Reflections on take home exercises with the couples
3. Introduce the session: today we will continue on power and control balance in relationship, how to have a healthy relationship among couples, and the role of male partners during pregnancy. Again, your participation is crucial in this session.
4. Prepare the quite place for lecture, materials like note book and pen
5. Start the teaching on the given topic

- Power and control balance in relationship
- How to have a healthy relationship among couples
- Role of male partners during pregnancy

1. Continue to lecture
2. Give time for questions or ask questions whether the couples understand or not

- Make sure that the couples responded at least 75 percent of the questions asked
- If the couples do not respond at least 75 percent of questions raised by the trainer, repeat the session for another time.

1. Introduce the take home exercise, appointment for next session and acknowledge the participants

**Session 5: triggers of IPV**

1. **Provide this session at fifth contact**
2. **Suggested time: 40 minutes**
3. **Objectives of the session**

- To discuss a health relationship in marital union
- To discuss the power and control balance in relationship
- To elaborate the roles of males during pregnancy

1. **Activity**

**Steps**

1. Warmly welcome the couples to the 5^th^ education session.
2. Reflections on take home exercises with the couples
3. Introduce the session: today we will continue on common triggers and methods of women empowerment. Furthermore, your participation has paramount role for the success of this session.
4. Prepare the quite place for lecture, materials like note book and pen
5. Start the teaching on the given topic

- Common triggers of IPV during pregnancy
- A healthy relationship in marriage
- The roles of male partners during pregnancy

1. Give time for questions or ask questions whether the couples understand or not

- Make sure that the couples responded at least 75 percent of the questions asked
- If the couples do not respond at least 75 percent of questions raised by the trainer, repeat the session for another time

**Session six 6: women empowerment**

1. **Provide this session at sixth contact**
2. **Suggested time: 40 minutes**
3. **Objectives of the session**

- To be familiar with the concepts of women empowerment with the couples
- To enrich the couples with the importance of women empowerment in prevention of IPV in pregnancy

1. **Activity**

**Steps**

1. Warmly welcome the couples to the 6^th^ education session.
2. Reflections on take home exercises with the couples
3. Introduce the session: today we will continue on women empowerment and of women empowerment. Furthermore, your participation has paramount role for the success of this session.
4. Prepare the quite place for lecture, materials like note book and pen
5. Start the teaching on the given topic

- Women empowerment
- Importance of women empowerment in IPV during pregnancy

1. Give time for questions or ask questions whether the couples understand or not

- Make sure that the couples responded at least 75 percent of the questions asked
- If the couples do not respond at least 75 percent of questions raised by the trainer, repeat the session for another time

1. Close up

Feedback from the couples

Acknowledge the couples

**The end!**

**Annex-V: Handout for Couple Based Violence Prevention Education (CBVPE)**

**Gender Based Violence (GBV)**

- Gender based Violence: refers to all forms of violence that happen to women, girls, men, and boys.
- It occurs due to unequal power relations between the victim and perpetrators.

**Violence against Women (VAW)**

- Violence against Women(VAW) is any act of gender-based violence that results in
  - - physical,
    - sexual,
    - psychological harm
    - suffering ,
    - threats ( acts, coercion, or arbitrary deprivations of liberty) to women
- It can be occurred in public or private life.

**Intimate Partner Violence (IPV)**

Intimate partner violence (IPV) refers any behavior within an intimate relationship that causes physical, psychological or sexual harm to the partner.

Intimate partner violence accounts for the lion share of Violence against Women.

Intimate partner violence occurs in the form of

- Physical,
- Sexual,
- Psychological or emotional,

**Physical intimate partner violence (IPPV) includes**

- Slap you or throw something that could hurt the partner
- Push the partner,
- Punch in abdomen
- Kick in abdomen
- shove the partner or pull the hair of the partner,
- Hit with his fist or with something else that could hurt the partner
- Kick ,
- Drag about or beat the partner up
- Attempt to choke or burn on purpose
- Threaten to use or actually use a gun, knife or other weapon against the partner

**Sexual Intimate Partner Violence (SIPV) includes**

- Physically force the partner to have sexual intercourse when she/he did not want to or
- Force the partner with threats or in any other way to perform sexual acts she/he did not or want
- Physically force the partner to perform any other sexual acts she /he did not want to

**Psychological or emotional Intimate Partner Violence (SEIPV) includes**

- The partner says or does something to humiliate her/him in front of another person or
- Threaten to hurt or harm her/him or someone she/he care about or
- Insult her him or make she/he feel bad about her/himself

**Magnitude of Intimate Partner violence (IPV)**

- Each year, between 1.5 and 5.3 million women are physically and/or sexually assaulted by an intimate partner.
- In Ethiopia, studies indicate that pregnancy intimate partner violence in pregnancy ranges from 26%-65%

**Session 2**

**Consequences of Intimate partner violence in pregnancy**

**Maternal health consequences**

***Physical***

- Injury(broken bone, burns, cuts, broken teeth, broken hands and legs)
- Physical impairment
- Lack of attachment to child
- Sexual and reproductive health problems(contracting sexually transmitted diseases, spread of HIV/AIDS, high-risk pregnancies, etc)
- Disability
- Homicide
- Antepartum hemorrhage
- Maternal death

***Psychological***

***Direct effect: anxiety, fear, mistrust of others, inability to concentrate, loneness,*** post-traumatic stress disorder, depression, homicide, suicide, etc.

***Behavioral***

- Multiple sexual partners
- Alcohol use during pregnancy
- Smoking during pregnancy
- Chat chewing
- Delayed antenatal care use

***Economic and social consequences***

- Rejection, ostracism, and social stigma at community level
- Reduced ability to participate in social and economic activities
- Acute fear of future violence, which extends beyond the individual survivors to other members in community
- Damage to women’s confidence resulting in fear of venturing into public spaces
- Increased vulnerability to other types of gender-based violence
- Job loss due to absenteeism as a result of violence
- Negative impact on women’s income generating power

***The impact on women’s family and dependents***

- Divorce, or broken families,
- Jeopardized family’s economic and emotional development ¸
- Premature birth or low birth weight
- Increased likelihood of violence against children growing up in households where there is domestic violence

**Session -3**

**Pregnancy consequences (adverse pregnancy outcomes)**

- Increased risk of intrauterine growth retardation
- Increased risk of preterm labour
- Increased risk of miscarriage
- Increased risk of unsafe abortion
- Increased risk of still birth
- Increased risk of perinatal death
- Low-birth weight
- Insufficient weight gain
- STI/HIV
- Neonatal death

***The impact of violence on perpetrators***

- Sanctioning by community
- Facing arrest and imprisonment
- Legal restrictions on seeing their families
- Divorce or the breakup of their families
- Feeling of alienation from their families
- Minimizing the significance of violence for which they are responsible
- Deflecting the responsibility for violence onto their partner and failure to associate it with their relationship
- Increased tension in the home

***The impact of violence on society***

- Burden on health and judicial systems
- Hindrance to economic stability and growth through women’s lost productivity
- Hindrance to women’s participation in the development processes and lessening of their contribution to social and economic development
- Constrained ability of women to respond to rapid social, political, or economic change
- Breakdown of trust in social relationships
- Weakened support networks on which people’s survival strategies depend.
- Strained and fragmented networks that are of vital importance in strengthening the capabilities of communities in times of stress and upheaval

**Importance of male involvement in maternal health care (Antenatal, delivery and postnatal)**

- Gets information on importance of antenatal care, facility delivery and postnatal check -up) from health care providers
- Reminds the partner (his wife) to antenatal appointments
- Arranges the transportation including money to go hospital or health center for antenatal check-up
- Yield benefits relating to maternal workload during pregnancy,
- Enables the couples ready for birth preparedness and complication readiness,
- Postnatal care attendance,
- Enables husbands communication and emotional support for women during pregnancy
- Improve child feeding practice including exclusive breast feeding
- Improves child immunization coverage
- Improves contraceptive use after child birth

Take-home exercise

Who make the final decision in the following activities?

- Household purchases including small and large ones,
- Visiting relatives and friends
- Spending the wife's earnings
- The number of children to have
- Obtaining health care for yourself

**Session-4**

***Power in relationships***

- Power in relationship enables the couples to control, make choices ,and have the capacity to impact the current circumstances
- A lack of power is a constant reminder of not being in control of our decisions or our destiny.

**Shared power**

- Occurs when partners are aware and confident about their self-worth and autonomy

**Power affects the relationship**

- In relationships that are strong and healthy, the influence both partners have is almost equal.
- One might have more financial power, the other more social connections, but ultimately they are respectful of one another and make decisions together

**Effects of imbalance of power in relationship**

- Damaged intimacy and relationship
- Intimate partner physical, sexual and psychological violence
- Frustration, anger, and depression
- Feelings of anxiety, fear and shame
- Impaired self-esteem, self-image and sense of person value
- Isolation, threats and abuse
- Lack of trust in the partner and endurance of the relationship
- Decreased overall satisfaction of the relationship
- End of relationship or marriage

**How to have healthy power balance in relationship of couples**

1. **Respect each other**

- Respect and trust are the foundations of any strong relationship
- Respect the views of your partner

1. **Make Money decisions together**

- Most of the time, the power balance in relationship is determined by money.
- Couples decide money expenditure together

1. **Develop good communication**

- One of the hallmarks of unhealthy or imbalanced power dynamics in relationship is lack of communication between couples.
- Both partners should have the freedom to address issues without fear
- Freedom to speak your mind is the key to a healthy relationship

1. **Increase your self-confidence**

- Lack of confidence or low self-esteem of one of the partners leads to power imbalance
- Work on your self-first
- Develop a healthy relationship with yourself,
- Learn to communicate your needs clearly and effectively

1. **Couples strive to meet relationship needs**

- Relationships are all bout give and take
- Realizing your own power as well as your partner one is key to a balanced and health relationship

**Role of husband/male partner during pregnancy**

“Pregnancy is a life changing phase for every woman”

“A wife supported by her husband will feel happier and less stressed”

1. **Accompany your wife during all medical appointments**

- Antenatal care
- During delivery
- Postnatal check-up
- Any medical check-up
- This allows the partner to follow the health condition of his wife and his baby
- Strengthen the emotional support for the women

1. **Participate in household chores**

- Understand that your wife is going through a transformation process that she needs
  - Rest
  - Support
  - Lots of understanding

1. **Communicate with your wife/partner**

- Very helpful to identify any anxiety that she has
- Opens the door to support your wife

1. **Prepare a birth plan**

- Financial preparation
- Prepare for transportation
- Plan for where to give birth

1. **Understand her moodiness**

- Hormonal changes in pregnancy may make moody due to discomfort, exhaustion and tiredness
- She may be happy at one second, may be any at another time
- Understand her moodiness

1. **Take care of her diet**

- You have to make sure that your wife is getting adequate nutrition.
- Encourage to eat more healthy food

1. **Get her enough sleep and rest**
2. **Become a good listener of your wife**
3. **Make change in your lifestyle**

- Avoid drinking too much coffee
- stop or avoid smoking around your pregnant wife
- Avoid drinking liquor

1. **Plan for walks**

Takes-home exercise

- What triggers of intimate partner violence during pregnancy in your family?
- How do you solve when disagreement appears in your relationship

**Session-4**

**Common triggers of Intimate partner violence**

- Alcohol use
- Drug abuse
- Ongoing anxiety of infidelity
- Mental health problems(depression, anxiety)
- Infidelity accusation
- Sexual jealousy

How to solve /minimize the triggers of intimate partner violence during pregnancy

- Avoid/minimize alcohol or alcohol use
- Talk things over-properly

If either of the partner has mistrust or misinformation, talk it over

Speak calmly, openly and honestly

- Don’t force things

It is not healthy to impose your beliefs on another person

Bring the trigger on the table and talk it over

- Don’t blame
- Make your relationship a priority
- Be honest to your partner
- Don’t lie
- Don’t dig up old wound
- Respect your partner’s boundary
- Don’t be jealous
- Be a good listener

**Session -6 women empowerment**

**Empowerment**: empowerment means that both women and men –can take control over their lives: set their agendas, gain skills (or have their own skills and knowledge recognized), increase self-confidence, solve problems, and develop self-reliance”

**Empowering women**: is ensuring that women participate fully in social, political and economic life.

When individuals are better able to make strategic choices in their lives, it has consequences for their economic and social well-being

Importance of empowering women

- - Effect of effective empowering of women
  - Free from violence
  - Have control on sexual and reproductive rights
  - Can get their voice herd
  - Have social and economic mobility
  - Have the ability to own and control financial assets
  - Have control over their income

**Ways of empowering women**

- End all forms of discrimination against all women and girls everywhere.
- Eliminate all forms of violence against all women and girls in the public and private spheres, including trafficking and sexual and other types of exploitation.
- Eliminate all harmful practices, such as child, early and forced marriage and female genital mutilation.
- Recognize and value unpaid care and domestic work through the provision of public services, infrastructure and social protection policies, and the promotion of shared responsibility within the household and the family
- Ensure women’s full and effective participation and equal opportunities for leadership at all levels of decision-making in political, economic and public life.
- Ensure universal access to sexual and reproductive health and reproductive rights
- Undertake reforms to give women equal rights to economic resources, as well as access to ownership and control over land and other forms of property, financial services,
- Expanding women education services

**Annex IA: ለጥንዶች መጠናዊ ጥናት የተሣታፊ መረጃ እና የስምምነት ቅጽ (Amharic version)**

**የተሳታፊዎች መረጃ ቅጽ**

**የጥናት ርዕስ፡** ጥንዶችን መሰረት ያደረገ ጥቃትን መከላከል ትምህርት በሀዲያ ዞን ደቡብ ምዕራብ ኢትዮጵያ

ዋና ተመራማሪ ፤ ዘለቀ ዱታሞ

**መግቢያ**

ጤና ይስጥልኝ ስሜ --------------------------------ይባላል፡፡ በዚህ ማህበረሰብ ውስጥ በአቶ ዘለቀ ዱታሞ (ኤም.ፒ.ኤች) ለሚካሄደው ምርምር ጥንዶችን መሰረት ያደረገ ጥቃትን መከላከል ትምህርት በምል ርዕስ በሀዲያ ዞን ደቡብ ምዕራብ ኢትዮጵያ ጥናት የጤና አስተማሪ/መረጃ ሰብሳቢ ሆኜ እየሰራሁ ነው፡፡አቶ ዘለቀ ዱታሞ በጅማ ዩኒቨርሲቲ የጤና ኢንስቲትዩት የዶክትሬት ዲግሪያቸውን በመከታተል ላይ ይገኛሉ፡፡ ስለ ጥናቱ እና የጥናቱ ተካፋይ ሆኖ መመረጥዎን ለማስረዳት ትኩረትዎን እንዲሰጡኝ በአክብሮት እጠይቃለሁ፡፡ የቅርብ አጋር(ባል) ጥቃት መከላከል ላይ ጥናት እያደረግን ነው እና በጥናቱ ላይ ተሳትፎዎን እናመሰግናለን፡፡ ይህ ቅጽ ስለ የጥናት ሂደቶች እና እንደ ተሳታፊ መብቶችዎ መረጃ ይሰጥዎታል፡፡ ተሳትፎ በፈቃደኝነት ነው። ምርጫዎን እና ውሳኔዎን እንዲወስኑ አሁን ስለዚህ ጥናት እና መረጃ እገልጽልዎታለሁ። ይህ የፈቃድ ቅጽ እርስዎ የማይረዱዋቸውን ቃላት ሊይዝ ይችላል። እባክዎን መረጃውን እንደምናልፍ እንዲያቆም ጠይቁኝ እና ለማብራራት ጊዜ ወስጃለሁ። በኋላ ላይ ጥያቄዎች ካሉዎት ከእኔ ወይም ከሌላ ተመራማሪ መጠየቅ ይችላሉ።

**የጥናቱ ዓላማ**

የዚህ ጥናት አላማ ለጥንዶች የሚሰጥ ትምህርት በሴቶች ጤና ላይ የተመሰረተ የጤና-ትምህርት ያለውን ውጤታማነት ለመገምገም ነው

**የጥናት ግብ:** የጥናቱ አላማ በጥንዶች ላይ የተመሰረተ የጤና ትምህርት - በሴቶች ጤና ላይ ያለውን ውጤታማነት መገምገም ነው

**የጥናት መርሃ ግብር እና የመረጃ አሰባሰብ፡-** ጥናቱ በክላስተር ውስጥ በተገኘው የጤና ጣቢያ ወይም ጤና ጣቢያ ስድስት የትምህርት ክፍለ ጊዜዎችን ያካትታል።

**የእርስዎ ኃላፊነት (ጥንዶች) ፡** ተመራማሪዎች እቤትዎ እንዲጎበኙዎት፣ በተመራማሪው ቡድን የተጠየቁትን ጥያቄዎች እንዲመልሱ ይጠበቃሉ። በተጨማሪም፣ በተመራማሪው ቡድን በሚቀርቡት 40-45 ደቂቃ የትምህርት ክፍለ ጊዜዎች ላይ እንድትገኙ ይጠበቃል፡፡

**ሚስጥር አጠባበቅ**፡ ለሚሰጡን መረጃ ሁሉም ምስጢርነቱ የተጠበቀ ነው፡፡ለዚሁምአርሶነትዎንና ቤትዎን የሚገልጽ ምንም ነገር የለም ፣የጥናቱ ውጤት ለግለሰብ ወይም ደግሞ ለቤት ብቻ ሳይሆን ለአጠቃላይ ህብረተሰብ የሚውል ይሆናል ፡፡ጥያቄው በኮድ ስለሆነ ምንም የእርሶን መልስ ከእረሶ ጋር የሚያያይዝ ነገር አይኖረም::

**ጥቅማ ጥቅሞች፡** ተሳታፊዎቹ ከኛ የምርምር ቡድን በሴቶች ጤና ላይ የነጻ ትምህርት ተጠቃሚ ይሆናሉ።

**ስጋቶች**፡ ጥናቱ ለተሳታፊዎች ምንም አይነት አደጋ የለውም። ከምርምር ጋር የተያያዙ ጉዳቶች አይጠበቁም፡፡ ይህ የሆነበት ምክንያት ምንም ዓይነት መድሃኒት, ኬሚካሎች ወይም ምግቦች ለተሳታፊዎች አይሰጡም፡፡

**በጎ ፈቃደኝነት፡** በጎ ፈቃደኝነት፡ ተሳትፎ በፈቃደኝነት ነው እና ማንኛውንም ጥያቄ ማቋረጥ ወይም ማቋረጥ ትችላለህ፣ ይህን ለማድረግ ነፃነት ይሰማህ:: እኛ ግን በጥናቱ እንድትሳተፉ እናበረታታዎታለን ምክንያቱም ግኝቶቹ የሴቶችን ጤና ለማሻሻል ፕሮግራሞችን ለመንደፍ ጠቃሚ ናቸው::

**ክፍያ፡** በጥናቱ ላይ ለመሳተፍ ወይም በመሳተፍዎ ምንም አይነት ክፍያ አያገኙም። በተመሳሳይ፣ በጥናቱ ለመሳተፍ ምንም አይነት ክፍያ አይጠየቁም።

**አድራ**ሻ

ስለጥናቱ አካሄድ ወይም ስለጥናቱ መጠይቅ ወይም ደግሞ ጥናቱን በተመለከተ ማንኛውም ጥያቄ ካሎት የሚከተሉትን አድራሻ ይጠቀሙ፡፡አቶዘለቀዱታሞ 0910489741/ 0930888897

ተጨማሪ የሚጠይቁት ነገር ካለ? ለመሳተፍ ፍቃደኛ ኖት?

አዎ፤ ቀጥል አይደለም፤ አቁም

**የስምምነትማረጋገጫፎርም**

የተሳታፊውን መረጃ ፎርም አንብቤዋለሁ ወይም ተነቦልኛል ፡፡የጥናቱ ዓላማ፣ ያለው ጉዳትና ጥቅም ምስጢር አጠባበቅ የመሳተፍ እና ያለመሳተፍ መብት እንዲሁም ችግር ካለ/ብፈጠር ከማንጋር መገኛኘት እንዳለብኝ ሁሉ ተገልጾልኝ ጥያቄ ካለኝ ደግሞ እንድጠይቅ እድል ተሰጥቶኝ በመሀል ደግሞ ጥናቱን ለማቆም ከፈለኩኝ በማንኛውም ጊዜ ከጥናቱ/ከተሳታፊነት/ መውጣት እንደምችል በመጨረሻም መመለስ የማልፈልገውን ጥያቄ አለመመለስ መብቱ እንዳለኝ ከተረዳሁኝ በኋላ በሙሉ ፈቃደኝነት በዚህ ጥናት ለመሳተፍ የወሰንኩኝ መሆኔን ከዚህ በታች በተቀመጠው ፊርማዬ አረጋግጣለሁ፡፡ስለዚህ፣ ከዚህ በታች በተገለፀው መሰረት በዚህ ጥናት ላይ ለመሳተፍ በፍቃዴ መስማማቴን በፊርሜ ወይም በግራ አውራ ጣት ህትመቴ አውጃለሁ።

የጥንዶች ኮድ------------------

የባል ፊርማ/ የጣት አሻራ.-------------------------

የሚስት ፍርማ/የጣት አሻራ-----------------------

**Annex VI: የአማርኛ መጠይቅ (የምስት)**

**መልሱን የያዘውን ፊደል በማክበብ ምረጡ ወይንም ክፍት ቦታውን የምሰጡትን ምላሽ በመጻፍ አሟሉ**

| **ክፍል አንድ፡ የግለሰብ፣ ግንኙነት፣ የማህበረሰብ እና የማህበረሰብ ደረጃ መጠይቆች** | | | |
| --- | --- | --- | --- |
| መለያ  ቁጥር | ጥያቄዎች | አማራጭመልስ | እለፍ |
| 101 | አሁን ዕድሜዎ ስንት ነዉ? | ሙሉ ዓመት…………….……… |  |
| 102 | ወደ ጋብቻ ስገቡ ዕድሜዎ ስንት ነበር? | ሙሉ ዓመት በቁጥር……………. |  |
| 103 | የሚኖሩበት ቦታ የት ነዉ? | ገጠር……………………………………….…1  ከተማ…………………………………………2 |  |
| 104 | ከተጋቡ ምን ያህል ዓመት ቆይተዋል? | ሙሉ ዓመት………………………………..1  አላዉቅም----------------------------------2 |  |
| 105 | የባሎት ዕድሜ ስንት ነዉ? | ሙሉ ዓመት _________________ |  |
| 106 | ሀይማኖትዎ ምንድነዉ? | ጴንጤ.................................................................1  ኦርቶዶክስ.........................................................2  ሙስሊም...............................................................3  ካቶሊክ..... ........................................................4  ሌላ ይገለጽ............................................................5 |  |
| 107 | የባሎት ሀይማኖት ምንድነዉ? | ኦርቶዶክስ.............................................................2  ሙስሊም.........................................................3  ካቶሊክ..... ............................................................4  ሌላ, ይገለጽ........................................................5 |  |
| 108 | ብሄሮት ምንድነዉ ነዉ? | ሀዲያ.................................................................1 ክምባታ...........................................................2  ስልጤ................................................................3  ጉራጌ.................................................................4  ሌላ, ይገለጽ......................................................5 |  |
| 109 | የባሎት ብሄር ምንድነዉ ነዉ? | ሀዲያ.................................................................1 ክምባታ...........................................................2  ስልጤ................................................................3  ጉራጌ.................................................................4  ሌላ, ይገለጽ......................................................5 |  |
| 110 | የትምህርት ደረጃዎት ስንት ነው? | ትምህርት የለም.................................................1  አንደኛ ደረጃ ትምህርት ቤት (1-8)......................2  ሁለተኛ ደረጃ ት/ቤት (9-12)………………..….3  ኮሌጅ ወይም ከፍተኛ ትምህርት ተከታትሏል …...4 |  |
| 111 | የባሎት የትምህርት ደረጃ ስንት ነዉ? | ትምህርት የለም.................................................1  አንደኛ ደረጃ ትምህርት ቤት (1-8)......................2  ሁለተኛ ደረጃ ት/ቤት (9-12)………………..….3  ኮሌጅ ወይም ከፍተኛ ትምህርት ተከታትሏል …...4 |  |
| 112 | ሥራዎት ምንድን ነው? | የቤት እመቤት ...................................................1  የመንግስት ተቀጣሪ...........................................2  የግል ስራ………………….……………….......3  ሌላ, ይገለጽ......................................................5 |  |
| 113 | የባሎት ስራ ምንድን ነዉ? | ገበሬ.................................................................1  የመንግስት ተቀጣሪ...........................................2  የግል ስራ………………….……………….......3  ሌላ, ይገለጽ......................................................5 |  |
| 114 | የትዳር አጋርዎ/ባልዎ በአሁኑ ጊዜ ሲጋራ ያጨሳል? | አዎ……………..…………………………......1  አይደለም………………………………….......2 |  |
| 115 | ባሎት/የትዳር አገርዎ በአሁኑ ጊዜ አረቄ /ቢራ ይጠጣል? | አዎ……………..…………………………......1  አይደለም………………………………….......2 |  |
| 116 | ባሎት ስንት ምስት አለዉ? | አንድ ………………………….……….……..1  ሁለትና ከዚያ በላይ………………….……........2 |  |
| **ክፍል ሁለት፡-ቤት እና ንብረትን የሚመለከቱ ጥያቄዎች** | | |  |

| 201 | የሚኖሩበት ቤት የተሰራው ከምንድን ነው? | | | | | 1.ከቆርቆሮ ክዳን | | | | 2.ከሳር ክዳን | | |  | |
| --- | --- | --- | --- | --- | --- | --- | --- | --- | --- | --- | --- | --- | --- | --- |
| 202 | የተለየ ምኝታ ክፍል አለዎት? | | | | | 1.አዎ | | 2.የለም | |  | | |  | |
| 203 | ለጥያቄ ቁ.202 መልሱ አዎ ከሆነ ስንት ክፍል? | | | | | 1.አንድ | | 2. ሁለት | | 3. ሶስት | | |  | |
| 204 | ከዋናው ቤት የተለየ የእንስሳት ቤት አለዎት? | | | | | 1.አዎ | | 2.የለም | |  | | |  | |
| 205 | ከዋናው ቤት የተለየ ማብሰያ ቤት አለዎት? | | | | | 1.አዎ | | 2.የለም | |  | | |  | |
| 206 | ከቤተሰብ አባላት ላም ያለው አለ? | | | | | 1.አዎ | | 2.የለም | | አዎ ካሉ ስንት-- | | |  | |
| 207 | ከቤተሰብ አባላት በሬ ያለው አለ? | | | | | 1.አዎ | | 2.የለም | | አዎ ካሉ ስንት-- | | |  | |
| 208 | ከቤተሰብ አባላት ጥጃ ያለው አለ? | | | | | 1.አዎ | | 2.የለም | | አዎ ካሉ ስንት-- | | |  | |
| 209 | ከቤተሰብ አባላት ከላይ ከተዘረዘሩት ሌላ ከብት ያለው አለ?(ጊደር፤ ወይፈን) | | | | | 1.አዎ | | 2.የለም | | አዎ ካሉ ስንት-- | | |  | |
| 210 | ከቤተሰብ አባላት ፈረስ ያለው አለ? | | | | | 1.አዎ | | 2.የለም | | አዎ ካሉ ስንት-- | | |  | |
| 211 | ከቤተሰብ አባላት አህያ ያለው አለ? | | | | | 1.አዎ | | 2.የለም | | አዎ ካሉ ስንት-- | | |  | |
| 212 | ከቤተሰብ አባላት በቅሎ ያለው አለ? | | | | | 1.አዎ | | 2.የለም | | አዎ ካሉ ስንት-- | | |  | |
| 213 | ከቤተሰብ አባላት በግ ያለው አለ? | | | | | 1.አዎ | | 2.የለም | | አዎ ካሉ ስንት-- | | |  | |
| 214 | ከቤተሰብ አባላት ፍየል ያለው አለ? | | | | | 1.አዎ | | 2.የለም | | አዎ ካሉ ስንት-- | | |  | |
| 215 | ከቤተሰብ አባላት ዶሮ ያለው አለ? | | | | | 1.አዎ | | 2.የለም | | አዎ ካሉ ስንት-- | | |  | |
| 216 | ከቤተሰብ አባላት ንብ ያለው አለ? | | | | | 1.አዎ | | 2.የለም | | አዎ ካሉ ስንት ቀፎዎች-- | | | |  |
| 217 | ከቤተሰብ አባላት መሬት ያለው አለ? | | | | | 1.አዎ | | 2.የለም | | አዎካሉ ስንትሄክታር--- | | | |  |
| 218 | ከቤተሰብ አባላት ሬድዮ ያለው አለ? | | | | | 1.አዎ | | 2.የለም | | አዎ ካሉ ስንት-- | | | |  |
| 219 | ከቤተሰብ አባላት ቴሌዠን ያለው አለ? | | | | | 1.አዎ | | 2.የለም | | አዎ ካሉ ስንት-- | | | |  |
| 220 | ከቤተሰብ አባላት ሞባይል ያለው አለ? | | | | | 1.አዎ | | 2.የለም | | አዎ ካሉ ስንት-- | | | |  |
| 221 | ከቤተሰብ አባላት የምግብ ጠረምጴዛ ያለው አለ? | | | | | 1.አዎ | | 2.የለም | | አዎ ካሉ ስንት-- | | | |  |
| 222 | ከቤተሰብ አባላት ሶፋ ወንበር ያለው አለ? | | | | | 1.አዎ | | 2.የለም | | አዎ ካሉ ስንት-- | | | |  |
| 223 | ከቤተሰብ አባላት ባለእስፖንጅ ፍራሽ አልጋ ያለው አለ? | | | | | 1.አዎ | | 2.የለም | | አዎ ካሉ ስንት-- | | | |  |
| 224 | ከቤተሰብ አባላት የሶላር መብራት ያለው አለ? | | | | | 1.አዎ | | 2.የለም | | አዎ ካሉ ስንት-- | | | |  |
| 225 | ከቤተሰብ አባላት ስዓት ያለው አለ? | | | | | 1.አዎ | | 2.የለም | | አዎ ካሉ ስንት-- | | | |  |
| 226 | ከቤተሰብ አባላት ብስክሌት ያለው አለ? | | | | | 1.አዎ | | 2.የለም | | አዎ ካሉ ስንት-- | | | |  |
| 227 | ከቤተሰብ አባላት ሞተር ያለው አለ? | | | | | 1.አዎ | | 2.የለም | | አዎ ካሉ ስንት-- | | | |  |
| 228 | ከቤተሰብ አባላት ጋሪ ያለው አለ? | | | | | 1.አዎ | | 2.የለም | | አዎ ካሉ ስንት-- | | | |  |
| 229 | ከቤተሰብ አባላት የባንክ ደብተር ያለው አለ? | | | | | 1.አዎ | | 2.የለም | | አዎ ካሉ ስንት-- | | | |  |
| 230 | ከቤተሰብ አባላት የጫት ማሳ ያለው አለ? | | | | | 1.አዎ | | 2.የለም | | አዎ ካሉ ስንት ሄክታር-- | | | |  |
| 231 | ከቤተሰብ አባላት የባህር ዛፍ ማሳ አለ? | | | | | 1.አዎ | | 2.የለም | | አዎ ካሉ ስንት ሄክታር-- | | | |  |
| **የሚከተሉትን እህል፤ ጥራጥሬ፤አትክልት እና ፍራፍሬዎች በዚህ አመት ምን ያህል እንዳመረቱ ጠይቁ** | | | | | | | | | | | | | | |
| 233 | ጤፍ-ኩ/ል | ዳጉሳ--ኩ/ል | | በቆሎ--ኩ/ል | ስንዴ--ኩ/ል | | ገብስ--ኩ/ል | | | | አጃ--ኩ/ል | ሩዝ--ኩ/ል | | |
| 234 | ባቄላ--ኩ/ል | አተር--ኩ/ል | | ምስር--ኩ/ል | ለውዝ--ኩ/ል | | ሽምብራ-ኩ/ል | | | | ጓያ--ኩ/ል | ሌላካለ ይጠቀስ---- | | |
| 235 | ጎመን--ኪ.ግ | | ካሮት--ኪ.ግ | | ድንች--ኪ.ግ | | | | ቲማቲም--ኪ.ግ | | | ጥቅልጎመን--ኪ.ግ | | |
|  | ቀይ ሽንኩርት--ኪ.ግ | | ነጭሽንኩርት--ኪ.ግ | | ቀይ ስር--ኪ.ግ | | | | ዱባ--ኪ.ግ | | | በርበሬ--ኪ.ግ | | |
| 236 | ማንጎ--ኪ.ግ | | ሙዝ--ኪ.ግ | | አቦካዶ--ኪ.ግ | | | | ፓፓያ--ኪ.ግ | | | ዘይቱና-ኪ.ግ | | |
|  | ሎሚ--ኪ.ግ | | ሌላ ካለ ይጠቀስ---- | | | | | | | | | | | |

|  | **ክፍል ሶስት፡ የቅርብ ጊዜ ቅድመ ወሊ እና ወሊድ እና በተመለከተ** | | | | | | | | | | | | |
| --- | --- | --- | --- | --- | --- | --- | --- | --- | --- | --- | --- | --- | --- |
| 301 | እስካሁን ስንት በህይወት ያለዉን ልጅ ወልደዋል(ስንት ጊዜ ወልደዋል) | | በቁጥር………………………………. | | | | | | | | | |  |
| 302 | ከዚህ በፊት ለነበረዉ የቅርብ ጊዜ እርግዝና የቅድመ ወሊድ ክትትል አድርገዉ ነበር? | | አዎ………………………….……………..….1  አይደለም…………………………….………..2 | | | | | | | | | | አይደለም  ከሆነ ወደ  **305 እለፊ** |
| 303 | የቅድመ ወሊድ ክትትል አድርገዉ ከሆነ ከየትኛዉ የጤና ተቋም ነዉ ስከታተሉ የነበረ? | | ሆስፕታል…………………….……………..….1  ጤናጣቢያ.…………………….…………..…..2  ክሊኒክ…………………….………………..….3  ጤና ኬላ--------------------------------…...------4  ሌላይገለጽ.…………………….…………..…..5 | | | | | | | | | |  |
| 304 | ላለፈው እርግዝና ወቅት ባሎት/የትዳር አጋርዎ ከእርስዎ ጋር የቅድመወሊድ ሲያደርጉ ከርሶ ጋር አብር ወደ ጤና ተቋም ሄዶ ያዉቃል ? | | አዎ……………………………….….…………1  አይደለም……………………………………….2 | | | | | | | | | |  |
| 305 | በቅርብ እርግዝና ወቅት በእርግዝና ወቅት የአደጋ ምልክቶች አጋጥሞ ያዉቃል ? | | አዎ……………………………….……………1  አይደለም…………………………………...….2 | | | | | | | | | |  |
| 306 | የቅርብ ልጅዎን የት ነው የወለዱት? | | ቤት…………………………………...………...1  ሆስፕታል…………………...………...………...2  ጤናጣቢያ…………………..………...……......3  ክሊኒክ.………………………………...…..…...4  ጤና ኬላ---------------------------------------5 | | | | | | | | | |  |
| 307 | ሆስስፕታል ወይም ጤና ጣቢያ ወይም ክሊኒክ ወይም ክሊኒክ ከሆነ የቅርብ ልጅዎን ስወልዱ ፣ባለቤቶት/የትዳር አጋሪዎ አብሮ ነበር? | | አዎ-------------------------------------------------------…1  አይደለም-------------------------------------------------2 | | | | | | | | | |  |
|  | **ክፍል አራት ፡ የአሁኑ እርግዝና በተመለከተ** | | | | | | | | | | | |  |
| 401 | የአሁኑን እርግዝና  ለማርገዝ ፈልገህ ነበር?  እስከ በኋላ ድረስ መጠበቅ ፈልገህ ነበር ? ወይስ  ምንም (ተጨማሪ) ልጅ መውለድ አይፈልጉም ነበር? | | ፈልጌ ነዉ …………..……………...…..…….....1  ከተወሰ ጊዜ በኋላ ነበር መርገዝ እፈልግ ነበር……..2  ማርገዝ ምንም አልፈልግም ነበር…………….….3 | | | | | | | | | |  |
| 402 | ለአሁኑ እርግዝናዎ የቅድመ ወሊድ ክትትል ከሰለጠኑ የጤና ባለሙያዎች (ከነርስ፣ አዋላጅ፣ ዶክተር ወይም የጤና መኮንን) ተገኝተዋል? | | አዎ……….……………………….……………1  አይደለም……………………………………….2 | | | | | | | | | | 2 አየደለም  ከሆነ ወደ 405 እለፊ |
| 403 | አዎ ከሆነ፣ የት ነዉ የቅድሚያ ወሊድ የተከታተሉት? | | ሆስፕታል……….……………………….……1  ጤናጣቢያ……….……………………….…..2  ክሊኒክ.......................................................3  ጤና ኬላ----------------------------…]-----------4  ሌላ, ይገለጽ.........................................................5 | | | | | | | | | |  |
| 404 | ለአሁኑ እርግዝና የልጅዎ አባት ከእርስዎ ጋር የቅድመ-ወሊድ ጉብኝት ተካፍሏል? | | አዎ……….……………………….……………1  አይደለም……………………………………….2 | | | | | | | | | |  |
| 405 | አሁን ባለው እርግዝና ወቅት በእርግዝና ወቅት የሚከሰቱ የጤና አደጋ ምልክቶችን አስተውለዋል? | | አዎ……….……………………….……………1  አይደለም……………………………………….2 | | | | | | | | | |  |
| 406 | በአሁኑ እርግዝና ላይ በነበሩበት ወቅት ሴትን ምን አይነት የጤና ችግሮች አጋጥሞወታል? ከአንድ በላይ ምልክት ሊደረግባቸው ይችላል (ምላሾችን አያነብቡ) | | አዎ አይደለም  በእርግዝና ወቅት በማህጸን  ከባድ የደም መፍሰስ...................1 2  እጆች / ፊት እብጠት................1 2  ከባድ ራስ ምታት....................1 2  የፅንስ እንቅስቃሴ አለመኖር........1 2  የደበዘዘ እይታ........................1 2  በወሊድ ጊዜ እና ልጅ በሚወልዱበት  ጊዜ ከባድ በማህጸን ደም መፍሰስ.1 2  ለረጅም ጊዜ የሚቆይ ሚጥ  መንቀጥቀጥ.............................1 2  እራስን መሳት ………………...1 2 | | | | | | | | | |  |
| 407 | ከዚህ እርግዝና በፊት የተወለደዉ ህጻን ጾታ ምንድነዉ? | | ወንድ………………………………………….1  ሴት……………………………..……………..2 | | | | | | | | | |  |
|  | **ክፍል አምስት : የሴቶች ራስን በራስ የማስተዳደር እና ማህበራዊ ድጋፍ** | | | | | | | | | | | |  |
| 500 | **የሴቶች በራሳቸዉ የመወሰን መጠይቅ** | | | | | | | | | | | |  |
|  | በሚከተሉት የቤት ዉስጥ ጉዳዮች ላይ የመጨራሻ ዉሳኔ ሰጪማን ነዉ?  1.ሚስትብቻ 2. ባልብቻ 3. ባልና ምስት አንድላይ 4 ሌላ ሰዉ/ የቤተብ አባል | | |  | | | | | | | | |  |
| 501 | ትናንሽ እና ትላልቅ ግዢዎችን ጨምሮ በቤተሰብ ግዢዎች ላይ? | | | 1 | 2 | | 3 | | | | 4 | |  |
| 502 | ዘመዶችን እና ጓደኞችን ለመጎበኛት? | | | 1 | 2 | | 3 | | | | 4 | |  |
| 503 | የሚስቱን ገቢ ወጪ በማድረግ? | | | 1 | 2 | | 3 | | | | 4 | |  |
| 504 | የሚወልዱ ልጆች ብዛት? | | | 1 | 2 | | 3 | | | | 4 | |  |
| 505 | ለእርስዎ የጤና እንክብካቤ ለማገኘት? | | | 1 | 2 | | 3 | | | | 4 | |  |
| 506 | **ማህበራዊ ድጋፍ** | | | | | | | | | | | |  |
| 507 | ምን ያህል ሰዎች ከእርስዎ ጋር በጣም ቅርብ ስለሆኑ ታላቅ የግል ችግሮች ካጋጠሙዎት በእነሱ ላይ መተማመን ይችላሉ? | | | የለም...................................................1  1–2.....................................................2  3–5.....................................................3  5+......................................................4 | | | | | | | | |  |
| 508 | ሰዎች በምታደርገው ነገር ምን ያህል ፍላጎት እና አሳቢነት ያሳያሉ? | | | ምንም………………………………1  ትንሽ……………………..……...…...2  እርግጠኛ አይደለሁም...........…………3  የተወነ……………….…………...…..4  ብዙ………………….………………5 | | | | | | | | |  |
| 509 | ከፈለጉ ከጎረቤቶች ተግባራዊ እርዳታ ማግኘት ምን ያህል ቀላል ነው? | | | በጣም ከባድ …...………………..…1  ከባድ…………..………….…………2  ይቻላል…………………..…..………3  ቀላል…………………….………...…4  በጣም ቀላል…..….………….………5 | | | | | | | | |  |
|  | **ክፍል ስድስት : የእውቀት፣ የአመለካከት እና የባህሪ መቆጣተር ጥያቄዎች** | | | | | | | | | | | |  |
| 600 | **እዉቀት** | | | | | | | | | | | |  |
| 601 | ስለ የቅርብ አጋር ጥቃት ሰምተዉ ያዉቃሉ? | | | አዎ ……………………….1  አላዉቅም----------------2 | | | | | | | | |  |
| 602 | የቅርብ አጋር ጥቃት ላይ የመረጃ ምንጮች ሚዲያ፣ሆስፕታል፣ጤና ጣቢያ ፣ጤና ጣቢያ ወዘተ..ናቸዉ? | | | አዎ ……………………….1  አላዉቅም----------------2 | | | | | | | | |  |
| 603 | የቅርብ አጋር( ጥቃት የህዝብ ጤና ጉዳይ ነው። | | | አዎ ………………………………….1  አይደለም……………..……………..2 | | | | | | | | |  |
| 604 | በጥፊ መምታት፣ መምታት፣ መጎተት፣ መደብደብ፣ ማነቅ፣ መግፋት፣ ወዘተ.የፍቅር አጋር ጥቃት ምሳሌዎች ናቸው። | | | አዎ ………………………………….1  አይደለም……………..……………..2 | | | | | | | | |  |
| 605 | ባልፈለገች ጊዜ ወሲብ እንዲፈጽም ማስገደድ ወሲባዊ ጥቃት ነው። | | | አዎ ………………………………….1  አይደለም……………..……………..2 | | | | | | | | |  |
| 606 | እሱ/እሷ የሚያዋርድ ወይም የሚያዋርድ ነገር እንዲፈጽም ማስገደድ ወሲባዊ ጥቃት ነው። | | | አዎ ………………………………….1  አይደለም……………..……………..2 | | | | | | | | |  |
| 607 | አጋርን በሌሎች ሰዎች ፊት ማዋረድ ወይም ማዋረድ የስነ-ልቦና ጥቃት ነው። | | | አዎ ………………………………….1  አይደለም……………..……………..2 | | | | | | | | |  |
| 608 | በእርግዝና ወቅት የቅርብ ወዳጃዊ ጥቃት የእናቶች እና አዲስ የሚወለዱ ሕፃናት ላይ አሉታዊ ውጤቶች አሉት፡፡ | | | አዎ ………………………………….1  አይደለም……………..……………..2 | | | | | | | | |  |
| 609 | የቅርብ አጋር /የትዳር አጋር ጥቃት የሰብአዊ መብት ጥሰት ነው። | | | አዎ …………………………….…….1  አይደለም……………..………..……..2 | | | | | | | | |  |
| 610 | **የአመለካከት ጥያቄዎች** | | | | | | | | | | | |  |
|  | በእርስዎ አስተያየት ባል በሚከተለው ሁኔታ ሚስቱን ለመምታት ይጸድቃል   1. አዎ 2. አይደለም | | | | | | | | | | | |  |
| 611 | ሳትነግረው ከወጣች? | | | | | 1 | | | 2 | | | |  |
| 612 | ልጆቹን ችላ ካላች? | | | | | 1 | | | 2 | | | |  |
| 613 | ከሱ ጋር ብትጨቃጨቅ? | | | | | 1 | | | 2 | | | |  |
| 614 | ከእሷ ጋር የግብረ ሥጋ ግንኙነት ለመፈጸም ፈቃደኛ ካልሆነች? | | | | | 1 | | | 2 | | | |  |
| 615 | ምግቡን ካቃጠለች? | | | | | 1 | | | 2 | | | |  |
| 616 | **የባህሪ መቆጣጠር ጥያቄዎች** | | | | | | | | | | | |  |
|  | በእርስዎ አስተያየት ባል በሚከተለው ሁኔታ ምስትን በመቆጣጠር ረገድ ትክክል ነው?   1. አዎ 2. አይደለም | | | | | | | | | | | |  |
| 617 | ከሌሎች ወንዶች ጋር ብታወራ መቅናት | | | | | 1 | | | | 2 | | |  |
| 618 | ታማኝነት በማጣት ብከሳት | | | | | 1 | | | | 2 | | |  |
| 619 | ከጓደኞቿ ጋር እንድትገናኝ አለመፍቀድ | | | | | 1 | | | | 2 | | |  |
| 620 | ከቤተሰብ ጋር ያላትን ግንኙነት ለመገደብ መምከር | | | | | 1 | | | | 2 | | |  |
| 621 | የት እንዳለች ለማወቅ ማጥበቅ | | | | | 1 | | | | 2 | | |  |
| 622 | በገንዘብ ባያምናት | | | | | 1 | | | | 2 | | |  |
| 700 | ይህ መጠይቅ ስለ እርስዎ የግል አመለካከት እና ባህሪያት ተከታታይ መግለጫዎች ይዞዋል፡፡ እባኮትን ጠያቂው በሚያነብበት ጊዜ ስለ እያንዳንዱ መግለጫ የራስዎን የግል እምነት ያመልክቱ ይህም የእርስዎን አመለካከት እና ስሜት በተሻለ ሁኔታ የሚገልጽ ነው  1. በፍፁም እውነት አይደለም  2. እምብዛም እውነት  3. መጠነኛ እውነት  4. በትክክል እውነት | | | | | ምርጫዉን አክብቢ | | | | | | |  |
| 701 | በቂ ጥረት ካደረግኩ ሁል ጊዜ አስቸጋሪ ችግሮችን መፍታት እችላለሁ | | | | | 1 | | 2 | | 3 | | 4 |  |
| 702 | አንድ ሰው የሚቃወመኝ ከሆነ የምፈልገውን ለማግኘት ስልቶችን እና መንገዶችን ማግኘት እችላለሁ | | | | | 1 | | 2 | | 3 | | 4 |  |
| 703 | በአላማ ላይ መጽናትና እና ግቦቼን ማሳካት ለእኔ ቀላል ነው | | | | | 1 | | 2 | | 3 | | 4 |  |
| 704 | ያልተጠበቁ ክስተቶችን በብቃት ማስተናገድ እንደምችል እርግጠኛ ነኝ | | | | | 1 | | 2 | | 3 | | 4 |  |
| 705 | ለሀብቴ ምስጋና ይግባውና ያልተጠበቁ ሁኔታዎችን እንዴት እንደማስተናግድ አውቃለሁ | | | | | 1 | | 2 | | 3 | | 4 |  |
| 706 | አስፈላጊውን ጥረት ካደረግኩ ብዙ ችግሮችን መፍታት እችላለሁ | | | | | 1 | | 2 | | 3 | | 4 |  |
| 707 | ችግሮች ሲያጋጥሙኝ መረጋጋት እችላለሁ ምክንያቱም በመቋቋም ችሎታዬ መታመን እችላለሁ | | | | | 1 | | 2 | | 3 | | 4 |  |
| 708 | ከችግሩ ጋር ሲያጋጥመኝ ብዙ መፍትሄዎችን ማግኘት እችላለሁ | | | | | 1 | | 2 | | 3 | | 4 |  |
| 709 | ችግር ካጋጠመኝ, ብዙውን ጊዜ መፍትሄውን ማሰብ እችላለሁ | | | | | 1 | | 2 | | 3 | | 4 |  |
| 710 | ብዙውን ጊዜ በመንገዴ የሚመጣውን ማንኛውንም ነገር መቋቋም እችላለሁ | | | | | 1 | | 2 | | 3 | | 4 |  |
|  | **ክፍል ሰባት : ከዚህ በፊት በነበራቸዉ እርግዝና ውስጥ የቅርብ አጋር(ባል) ጥቃት በተመለከተ** | | | | | | | | | | | | |
| 801 | **አካላዊ ጥቃት**  በቅርብ እርግዝና ወቅት, የትዳር ጓደኛዎ : | 1.አዎ 2. አይደለም  በጥፊ መትተዋሉ ወይም ሊጎዳህ የሚችል ነገር ወርውሮቦታል ?..1 2  ገፍትሯሉ፣አናዉጠዋሉ ወይም ፀጉሮትን ጎትተዋሉ……..1 2  በጥፊ ወይም በሌላ ሊጎዳዎት በሚችል ነገር መትተዋሉ?.,.1 2  መትተዋሉ፣ ጎትተዋሉ ወይስ ደብድበዋሉ?....................1 2  ሆን ብለዉ እርሶን ለማፈን ወይም ለማቃጠል ሞክረዋሉ?..1 2  በእርሶ ላይ ሽጉጥ፣ ቢላዋ ወይም ሌላ መሳሪያለመጠቀም ወይም በትክክል ለመጠቀም አስፈራርተዋሉ?....................1 2 | | | | | | | | | | |  |
| 802 | **ስነ-ልቦናዊ**  በቅርብ እርግዝና ወቅት, የትዳር ጓደኛዎ ፣ | 1.አዎ 2. አይደለም  እርሶን በሌላ ሰው ፊት ለማዋረድ አንድ ነገር ለመናገር  ሞክረዋሉ ወይስ ተናግረዋሉ?.................................1 2  እርስዎን ወይም እርስዎን የሚንከባከቡትን ለመጉዳት አስፈራርተዋሉ?………………………..……..1 2  ሰድቦዋሉ ወይም እርሶ መጥፎ ስሜት እንዲሰማ አድርገዋሉ?………………….…………………1 2  ሆን ብለዉ ማንቋሸሽ ወይም ማስፈራራት  አድርገዋል (ለምሳሌ፡ በአመለካከት  በመጮህ እና ነገሮችን በመሰባበር)?.........................1 2 | | | | | | | | | | |  |
| 803 | **ወሲባዊ**  በቅርብ እርግዝና ወቅት, የትዳር ጓደኛዎ | 1.አዎ 2. አይደል  እረሶ ሳይፈልጉ በጉልበት ወሲብ  ለመፈጸም አስገድደዋሉ?........................................1 2  በጉልበት በማስፈራራት ወይም በሌላ በማንኛዉም  መንገድ ሳይፈልጉ ወሲብ ፈጽመዋል?...................1 2  እርሲ ሳይፈልጉ በጉልበት ሌላ የወሲብ  ንክክዎችን እንድፈጽሙ አድርገዋሉ?……….…....1 2 | | | | | | | | | | |  |
| 804 | ከሶስቱ (801፣802እና 803) ብያንስ አንዱ አዎ ከሆነ፣ ምን አደረጉ? | ምንም…………………………………………………………..1  ለጓደኞቹ አመልክተዋሉ……………………………………..........2  ግንኙነት ማቋረጥ……………………………………………...…3 | | | | | | | | | | | ምንም ከሆነ ወደ 805 እለፊ |
| 805 | ምንም ካላደረጉ(ጥያቄ 804(1))፣ለምን | 2. አዎ 2. አይደለም  ሁኔታው አላግባብ የመሆኑ እርግጠኛ አለመሆን ……....1 2  ፍርሃት……………………………………………….1 2  ይቅርታ አልኩት……………………………………....1 2  ማፈር እና መሸማቀቅ…………………….………..,,..1 2 | | | | | | | | | | |  |

**Annex vii: የባሎች መጠይቅ -አማርኛ ትርጉም**

መጠይቁ የተሞላበት ቀን (በኢት. አቆጣጠር)------------

የመጠይቅ ቁጥር--------------------

የጠያቂ መለያ ቁጥር-----------------

መጠይቁ የተጠየቀበት ቀበል-----------

መጠይቅ የተጀመረበት ሰዓት-----------

መጠይቅ ያለቀበት ሰዓት---------------

|  | | | | **ክፍል አንድ፡ ስነ ማሀበራዊ መጠጥቅ** | | | | | | | | |  |  |
| --- | --- | --- | --- | --- | --- | --- | --- | --- | --- | --- | --- | --- | --- | --- |
| መለያ  ቁጥር | | | | ጥያቄዎች | | አማራጭ መልስ | | | | | | | እለፍ |  |
| 101 | | | | አሁን ዕድሜዎት ስንት ነዉ ? | | ሙሉ ቁጥር ዕድሜ…………………………….. | | | | | | |  |  |
| 102 | | | | የት ነዉ የሚኖሩት ? | | ገጠር ………………………………………….…1  ከተማ ……………………………………………2 | | | | | | |  |  |
| 103 | | | | በትዳር ዉስጥ ምን ያክል ጊዜ ቆይተዋሉ? | | ሙሉ ዓመት/ወር…………………………… | | | | | | |  |  |
| 104 | | | | የባለቤቶት ዕድሜ ስንት ነዉ? | | ዕድሜን በሙሉ ቁጥር…………………………… | | | | | | |  |  |
| 105 | | | | ሀይማኖቶት ስንት ነዉ? | | ፕሮቴስታንት .........................................................1  ኦርቶዶክስ.............................................................2  ሙስሊም ..............................................................3  ካጦሊክ ..............................................................4  ሌላ , ይገለጽ.........................................................5 | | | | | | |  |  |
| 106 | | | | የባላቤቶት ሐይማኖት ነዉ? | | ፕሮቴስታንት .........................................................1  ኦርቶዶክስ.............................................................2  ሙስሊም ..............................................................3  ካጦሊክ ..............................................................4  ሌላ , ይገለጽ.........................................................5 | | | | | | |  |  |
| 107 | | | | ብሄሮት ምንድነዉ? | | ሀዲያ.................................................................1 ክምባታ.............................................................2  ስልጤ................................................................3  ጉራጌ.................................................................4  ሌላ, ይገለጽ......................................................5 | | | | | | |  |  |
| 108 | | | | የባለቤቶት ብሔር ምንድነዉ? | | ሀዲያ.................................................................1 ክምባታ..............................................................2  ስልጤ................................................................3  ጉራጌ.................................................................4  ሌላ, ይገለጽ......................................................5 | | | | | | |  |  |
| 109 | | | | የትምህርት ደረጃዎት ስንት ነዉ? | | ትምህርት የለም.................................................1  አንደኛ ደረጃ ትምህርት ቤት (1-8)......................2  ሁለተኛ ደረጃ ት/ቤት (9-12)………………..….3  ኮሌጅ ወይም ከፍተኛ ትምህርት ተከታትሏል …...4 | | | | | | |  |  |
| 110 | | | | የባለቤቶት ትምህርት ደረጃ ምነድነዉ? | | ትምህርት የለም...................................................1  አንደኛ ደረጃ ትምህርት ቤት (1-8)..........................2  ሁለተኛ ደረጃ ት/ቤት (9-12)………………..…….3  ኮሌጅ ወይም ከፍተኛ ትምህርት ተከታትሏል ……...4 | | | | | | |  |  |
| 111 | | | | ሥራዎት ምንድነዉ? | | ገበሬ....................................................................1  የመንግስት ተቀጣሪ..............................................2  የግል ስራ………………….…………….…….......3  ሌላ, ይገለጽ.........................................................5 | | | | | | |  |  |
| 112 | | | | የባለቤቶት ስራ ምንድነዉ? | | የቤት እመቤት ...................................................1  የመንግስት ተቀጣሪ...........................................2  የግል ስራ………………….…………………......3  ሌላ, ይገለጽ......................................................5 | | | | | | |  |  |
| 113 | | | | አማካይ የቤተሰብዎ ወርሃዊ ገቢ በብር ስንት ነው?? | | ብር በወር (የመላሽ ግምት)……………………………… | | | | | | |  |  |
| 114 | | | | ስንት ምስት አሎት? | | አንድ…………...……..………………...….......1  ሁለትና ከዚያ በላይ ……………...……….........2 | | | | | | |  |  |
|  | | | | **ክፍል አራት : የእውቀት፣የአመለካከትና ባህሪ መቖጣተር ጥያቄዎች** | | | | | | | |  | | |
| 401 | | | ስለ የቅርብ አጋር ጥቃት ሰምተዉ ያዉቃሉ? | | | | | 1.አዎ  2.አላዊቅም | | | |  | | |
| 402 | | የቅርብ አጋር ጥቃት ላይ የመረጃ ምንጮች ምንድናቸው? | | | | | | **1.አዎ 2. አይደለም**  ሚዲያ …………………….…1 2  የግል ልምድ…………......……1 2  ሆስፕታል/ጤና ጣቢያ/ጤና ኬላ..1 2  ከጤና ኤክስቴንሽ ባለሙያዎች…..1 2  ከሌክተር…………………....1 2 | | | |  | | |
| 403 | | የቅርብ አጋር( ጥቃት የህዝብ ጤና ጉዳይ ነው። | | | | | | አዎ ………………………………….1  አይደለም……………..……………..2 | | | |  | | |
| 404 | | በጥፊ መምታት፣ መምታት፣ መጎተት፣ መደብደብ፣ ማነቆ፣ መግፋት፣ ወዘተ.የፍቅር አጋር ጥቃት ምሳሌዎች ናቸው። | | | | | | አዎ ………………………………….1  አይደለም……………..……………..2 | | | |  | | |
| 405 | | የትዳር አጋሮ ባልፈለገች ጊዜ ወሲብ እንዲፈጽም ማስገደድ ወሲባዊ ጥቃት ነው። | | | | | | አዎ ………………………………….1  አይደለም……………..……………..2 | | | |  | | |
| 406 | | እሱ/እሷ የሚያዋርድ ወይም የሚያዋርድ ነገር እንዲፈጽም ማስገደድ ወሲባዊ ጥቃት ነው። | | | | | | አዎ ………………………………….1  አይደለም……………..……………..2 | | | |  | | |
| 407 | | አጋርን በሌሎች ሰዎች ፊት ማዋረድ ወይም ማዋረድ የስነ ልቦና ጥቃት ነው። | | | | | | አዎ ………………………………….1  አይደለም……………..……………..2 | | | |  | | |
| 408 | | በእርግዝና ወቅት የቅርብ ወዳጃዊ ጥቃት የእናቶች እና አዲስ የሚወለዱ ሕፃናት ላይ አሉታዊ ውጤቶች አሉት፡፡ | | | | | | አዎ ………………………………….1  አይደለም……………..……………..2 | | | |  | | |
| 409 | | የቅርብ አጋር ጥቃት የሰብአዊ መብት ጥሰት ነው። | | | | | | አዎ…………………………..………….….1  አይደለም…………………………………...2 | | | |  | | |
| **የአመለካከት ጥያቄዎች** | | | | | | | | | | | |  | | |
|  | በእርስዎ አስተያየት ባል በሚከተለው ሁኔታ ሚስቱን ለመምታት ይጸድቃል  1.አዎ  2. አይደለም | | | | | | | | | | |  | | |
| 410 | ሳትነግረው ከወጣች? | | | | | | 1 | | 2 | | |  | | |
| 411 | ልጆቹን ችላ ካላት? | | | | | | 1 | | 2 | | |  | | |
| 412 | ከሱ ጋር ብትጨቃጨቅ? | | | | | | 1 | | 2 | | |  | | |
| 413 | ከእሷ ጋር የግብረ ሥጋ ግንኙነት ለመፈጸም ፈቃደኛ ካልሆነ? | | | | | | 1 | | 2 | | |  | | |
| 414 | ምግቡን ካቃጠለች? | | | | | | 1 | | 2 | | |  | | |
| **የባህሪ መቆጣተር ጥያቄዎች** | | | | | | | | | | | |  | | |
|  | | | | |  | | | |  | | |  | | |
|  | | | | በእርስዎ አስተያየት ባል በሚከተለው ሁኔታ ምስትን በመቆጣጠር ረገድ ትክክል ነው?   1. አዎ 2. አይደለም | | | | | | | |  | | |
| 415 | | | | ከሌሎች ወንዶች ጋር ብታወራ ቅናት | | | | | | 1 | 2 |  | | |
| 416 | | | | ታማኝነት በማጣት ብከሳት | | | | | | 1 | 2 |  |  |  |
| 417 | | | | ከጓደኞቿ ጋር እንድትገናኝ አለመፍቀድ | | | | | | 1 | 2 |  |  |  |
| 418 | | | | ከቤተሰብ ጋር ያላትን ግንኙነት ለመገደብ መምከር | | | | | | 1 | 2 |  |  |  |
| 419 | | | | የት እንዳለች ለማወቅ ማጥበቅ | | | | | | 1 | 2 |  |  |  |
| 420 | | | | በገንዘብ ባያምናት | | | | | | 1 | 2 |  |  |  |
|  | | | | **ክፍል አምስት : የባሎች ተሳትፎ በቅርብ ጊዜ እርግዝና** | | | | | | | |  | | |
| 501 | | | | ከዚህ በፊት ለነበረዉ የቅርብ ጊዜ እርግዝና ባለቤቶት የቅድመ ወሊድ ክትትል አድርገዉ ነበር? | | | አዎ…………………………………….….…...1  አይደለም…………….……………….…….…2 | | | | |  | | |
| 502 | | | | አዎ ከሆነ፣ ከባለቤቶት ጋር ቢያንስ አንድ ጊዜ ቅድመ ወሊድ ክትትል አድርገዋሉ? | | | አዎ………………………………….………....1  አይደለም……………………………….….…2 | | | | |  | | |
| 503 | | | | አይደለም ከሆነ፣ ለምንድነዉ ቅድመ ወሊድ ክትትል ያላደረጉት ? (**ምርጫዎችን አይነበቡ).**  **ከአንድ መልስ በላይ ማክበብ ይቻላል**) | | | ክትትል የሴቶች ጉዳይ ስለሆነ…………………...1  ለመሄድ ጊዜ ስለለሌኝ…………………….….…2  እዉቀት ስለለለኝ…………………………..3  ማህበራዊ መገለል ስለሚደርስብኝ …………4  ስለሚፈራ/ስለምሳቀቅ……………………….....5  የኤች.አይ.ቪ ምርመራ ስለሚፈራ………….…....6  ስለማልጋበዝ……………….…………….……..7  ሌላ፣ ይገለጽ……………………………….……8 | | | | |  | | |
| 504 | | | | ባለቤትዎ የቅርብ ጊዜ እርግዝናን የት ነበር የወለዱት ? | | | ሆስፕታል--------------------------------------1  ጤና ጣቢያ------------------------------------2  ክሊኒክ----------------------------------------3  ቤት-------------------------------------------4 | | | | |  | | |
| 505 | | | | ጤና ተቋም(ሆስፕታል፣ጤና ጣቢያ ዎይም ክሊኒክ) ከሆነ፣ ባለቤቶት ስወልዱ እርሶ አብሮ ነበሩ ? | | | አዎ-------------------------------------------1  አይደለም-------------------------------------2 | | | | |  | | |
| 506 | | | | በላቤቶት ከወለዱ በሓላ ለድክረ ወሊድ እንክብካቤ አብረዉ ጤና ተቋም(ሆስፕታል፣ጤና ጣቢያ ወይም ክሊኒክ ) ሄደዉ ያዉቃሉ ? | | | አዎ-------------------------------------------1  አይደለም-------------------------------------2 | | | | |  | | |

**Annex viii: ጥንዶች ላይ የተመሰረተ ጥቃትን መከላከል ጥቅል መመሪያ-የትምህርት ጥቅል ለጥንዶች**

**መግቢያ**

የባለትዳሮች ትምህርት ፓኬጅ በሀዲያ ዞን፣ ደቡብ ምዕራብ እና ኢትዮጵያ ውስጥ ባሉ ጥንዶች በእርግዝና ወቅት የሚደርስ የቅርብ ወዳጃዊ ጥቃትን ለመቀነስ የለውጥ ሂደትን ለማገዝ የተነደፈ የመከላከያ መልእክቶች ስብስብ ነው። በእርግዝና ወቅት የቅርብ አጋሮች ጥቃትን ለመከላከል በትምህርት ሞጁል ውስጥ የተካተቱት ይዘቶች የሚከተሉትን ያጠቃልላል፡፡

- ሥርዓተ ጾታን መሠረት ያደረጉ ጥቃቶች፣
- በሴቶች ላይ የሚደርሱ ጥቃቶች እና የቅርብ አጋር ጥቃቶች።
- የጥቃት ዓይነቶች (አካላዊ፣ ጾታዊ እና ስነ ልቦናዊ ወይም ስሜታዊ ጥቃት
- በሴቶች ላይ የሚደርሱ ጥቃቶች መጠን፣ ተፈጥሮ እና በእርግዝና ወቅት የቅርብ ጓደኛሞች ጥቃትን ጨምሮ።
- ቅርብ አጋር ጥቃት እና የስልጣን እና ቁጥጥር ግንኙነት
- ሴቶች ማብቃት የሚያስከትላቸው ውጤቶች
- ሥርዓተ ጾታ፣ኃይል እና ጾታዊነት
- በእርግዝና ወቅት የአይፒቪ የተለመዱ ቀስቅሴዎችጤናማ ግንኙነትን የሚያመጣው ምንድን ነው?

**የክፍለ-ጊዜዎች መዋቅር**

በአጠቃላይ ወደ ስድሰት የሚሆን የትምህርት ክፍለ -ጊዜ ይኖራል፡፡ እያንዳንዱ ክፍለ ጊዜ ከ60 እስከ 90 ደቂቃ ይወስዳል፡፡

**ጥንዶች በቤት ውስጥ መልመጃዎችን እና የመልመጃዎች መልስ**

አንዳንድ ክፍለ ጊዜዎች በክፍለ-ጊዜው መጨረሻ ላይ የሚመደቡትን የቤት ውሰዱ ልምምዶችን ይይዛሉ፣ እና ጥንዶች በራሳቸው ህይወት ውስጥ እንዲያስቡ እና አዳዲስ ሀሳቦችን እንዲተገብሩ ለመርዳት፡፡ ከስልጠና በኋላ በቤት ውስጥ የአካል ብቃት እንቅስቃሴዎችን ማድረግ የፕሮግራሙን ውጤታማነት በእጅጉ እንደሚጨምር ጥናቶች ያሳያሉ። እያንዳንዱ ክፍለ ጊዜ፣ በተመሳሳይ፣ በቤት ውሰድ የአካል ብቃት እንቅስቃሴን በማንፀባረቅ ይጀምራል።

የዚህ ነጸብራቅ ቅርጸት ለእያንዳንዱ ክፍለ ጊዜ አንድ አይነት ነው, የተለያዩ የመመሪያ ጥያቄዎችን ለማቃለል

**ተሳታፊዎች**

ሞጁሉ በተመረጡ ስልጠና በሚሰጥባቸዉ ቦታዎች ላይ ላሉ ጥንዶች ይሰጣል፡፡ ትምህርቱ የሚካሄደው በጤና ጣቢያ ወይም በጤና ኬላ በቡድን ነው።

**አስፈላጊ አቅርቦቶች**

ለሥልጠናው ቀላል የሥልጠና አቅርቦቶች፣ ገበታ ወረቀት፣ ፖስተር፣ ማስታወሻ ደብተር፣ እስክሪብቶ እና እርሳሶች እና የእርሳስ ሹል ያስፈልጋሉ። ክፍለ-ጊዜዎች የተነደፉት በተዘጋ ዎርክሾፕ ቦታ ላይ እንደሚተገበሩ በመረዳት ነው። ገር ግን ይህ ከሌለ ማመቻቻዎች ሊደረጉ ይችላሉ. በባለትዳሮች እና በጤና ኤክስቴንሽን ባለሙያ አስተማሪዎች መካከል ግንኙነት እና ውይይት ለመፍጠር እንዲረዳ ወንበሮችን በግማሽ ክበብ ውስጥ ማዘጋጀት ይመከራል።

**ቋንቋ**

በዚህ የሥልጠና ሞጁል የአማርኛ ቋንቋ ጥቅም ላይ ይውላል

**የሥልጠና ሞጁል ዓላማ**

1. ተሳታፊዎችን የእናቶች ጤና፣ጾታን መሰረት ያደረገ ጥቃት እና የትዳር አጋር ጥቃትነ ምን ማለት እንደሆነ ስለማሳወቅ
2. ጥንዶችን ሴቶችን መሰረት ያደረገ ጥቃት ልከ-መጠንን እና በእረግዝና ወቅት የሚከሰት ሴቶች ላይ ጥቃት የሚያመጣቸዉ ጉዳቶችን ለማሳወቅ
3. ጥንዶች በእርግዝና ወቅት የሚከሰት ጥቃት የሚያመጣቸዉን ጉዳቶች እንዲያዉቁ ማስቻል
4. ጥንዶች በእረግዝና ወቅት ለሚከሰቱ ጥሰት/ትቃት መንስኤዎች ምን እንደሆኑ እንዲያዉቁ ማስቻልና ጤናማ ግኑኝነት እንዴት መፍጠር እንደሚቻል ማሳወቅ
5. ባሎች በእናቶች ጤና እንክብካቤ መሳተፍ የሚኖረዉን ጥቅም ማሳወቅ
6. ጥንዶችን የሴቶች ማብቃት በቅርብ አጋር ጥቃት/ጥሰት ላይ የሚኖረዉን ተጽኖ ማሳወቅ

የእያንዳንዱ ክፍለ-ጊዜ የሚሰጡ የት/ት ይዘቶች

| ት/ት ይዘት | የምወስደዉ ደቂቃ |
| --- | --- |
| **ክፍለ-ጊዜ አንድ1(በመጀመሪያ ጊዜ)** | **45 ደቂቃ** |
| - ትዉዉቅ | 10 ደቂቃ |
| - የእናቶች ጤና ምን ማለት እንደሆነ - ጾታን መሰረት ያደረገ ጥቃት ምን ማለት እነደሆነ - ሴቶች ላይ የሚደርስ ጥቃት/ጥሰት ምንእንደሆነ - የቅረብ ትዳር አጋር ጥቃት/ጥሰት ምን እንደሆነ - የጥቃት/ጥሰት አይነቶች - የቅርብ የትዳር አጋር ጥቃት ልከ-መጠን | 30 ደቂቃ |
| ማጠቃለያና እና የሚቀጥለዉ ክፍለ-ጊዜ ቀጠሮ መያዝ | 5 ደቂቃ |
| **ክፍለ ጊዜ ሁለት (በሂለተኛ ቀጠሮ)** | **40 ደቂቃ** |
| **በእርግዝና ወቅት የሚከሰት ጥቃት የሚያመጣቸዉ መዘዞች**   - በእናቶች ላይ የሚያመጣ መዘዝ - ኢኮኖሚያዊና ማህበራዊ መዘዝ - የቤተሰብና ጥገኝነት መዘዝ | 30 ደቂቃ |
| - የቤት ስራ ማብራሪያ እና ለምቀጥለዉ ክፍለ-ጊዜ ቀጠሮ መያዝ | 10 ደቂቃ |
| **ክፍለ-ጊዜ ሶስት(በሶስተኛ ቀጠሮ)** | 45 ደቂቃ |
| የቤት ስራ ግብረ-መልስና ማብራሪያ | 10 ደቂቃ |
| - ጥቃት በእርግዝና ላይ የሚያስከትለዉ መዘዝ   ቀጥታና ተዘዋዋሪ   - ጥቃት ፈጻሚዎች ላይ የሚያመጠዉ መዘዝ - ማህበረሰብ ላይ የሚያመጣ መዘዝ - ባሎች በእናቶች ጤና እንክብካቤ ላይ መሳተፍ ጥቅሞች | 30 ደቂቃ |
| **ክፍለ -ጊዜ አራት (በአራተኛ ቀጠሮ)** | 45 ደቂቃ |
| የቤት ስራ ማብራሪያ እና ለምቀጥለዉ ክፍለ-ጊዜ ቀጠሮ መያዝ | 10 ደቂቃ |
| - የሀይል ምዛን - ጤናማ የሀይል ሚዛን በትዳር ግኑኝነት ዉስጥ - በእረግዝና ወቅት የትዳር አጋሮች ሚና | 25 ደቂቃ |
| የቤት ስራ ማብራሪያ እና ለምቀጥለዉ ክፍለ-ጊዜ ቀጠሮ መያዝ | 5 ደቂቃ |
| **ክፍለ-ጊዜ አምስት(በአራተኛ ቀጠሮ)** | **40 ደቂቃ** |
| - የቤት ስራ ግብረ-መልስና ማብራሪያ | 10ደቂቃ |
| - የተለመዱ የትዳር አጋር ጥቃትን የሚያበብሱ ነጋሮች - በእረግዝና ወቅት የትዳር አጋር ጥቃትን እንዴት መቅረፍ እንደምቻል | 30ደቂቃ |
| **ክፍለ-ጊዜ ስድስት (የመጨራሻ ቀጠሮ)** | 40 ደቂቃ |
| - ሴቶችን ማብቃት ጥቅሞች   ሴቶችን ማብቂያ መንገዶች | 30ደቂቃ |
| - ምስጋናና መዝጊያ | 10 ደቂቃ |

**ክፍለ-ጊዜ አንድ ፡ማብራሪያ በእናትነት ጤና፣ጾታን መሰረት ያደረገ ጥቃት፣ የሴቶች ጥቃት እና የቅረብ አጋር ጥቃት**

1. ይህ ክፍል በመጀመሪያ ቀጠሮ ላይ ይሰጣል
2. የምወስደዉ ሰዓት: 45 ደቂቃ
3. የክፍለ-ጊዜ ዋና አላማዎቸ

ጥንዶችን የእንትንት ጤና፣ ጾታን መሰረት ያደረገ ጥቃት፣ ሴቶች ላይ የሚፈጸም ጥቃት እና የትዳር አጋር ጥቃት ምን እንደሆነ ማስጨበጥ

ለጥንዶች በእርግዝና ወቅት የሚከሰት ጥቃቶችን ማስጨበጥ

ለጥንዶች የትዳር አጋር ጥቃቶች በእርግዝና ወቅት ያለዉን ልከ-መጠን ማብራራት

1. **ክንዉኖች**

**ደረጃዎች ቅደም ተከተል**

1. ወደ መጀመሪያ የት/ት ክፍለ ጊዜ እንኳን ዳህና መጣችሁ ብሎ መቀበል
2. የእለቱን መማሪያ ረእሶችን ማሳወቅ እንድሁም የሚኖረዉን ጥቅም
3. የመማሪያ ደብትር ፣ እስክብርቶ እና ፖስተር ማዘጋጀት
4. በሚከተሉት ርዕሶች ላይ ማስተማር

- የእናቶች ጤና
- ጾታን መሰረት ያደረገ ጥቃት
- ጤችን መሰረት ያደረገ ጥቃት?
- በትዳር አጋር የሚፈጸም ጥቃት
- በትዳር አጋር የሚፈጸሙ የጠቃት አይነቶች
- ከቅርብ/ትዳር አጋር የሚፈጸም ጥቃት ልከ-መጠን

1. ለጥያቄ ጊዜ መስጠት እና ተሳታፊዎቹ መረዳታቸዉን ማረጋገጥ

- ብያንስ ጥንዶቹ ቢያንስ 75% ጥያቄ መመላሳቸዉን ማረጋገጥ ካልሆነ ሌላ ትምህርት ክፍለ ጊዜ መያዝ

1. የሚቀጥለዉን ክፍለ-ጊዜ ቀጠሮ መያዝ እና ተሳታፊቺን ማመስገን

**ክፍለ-ጊዜ ሁለት: በእርግዝና ወቅት የሚከሰተዉ ጥሰት መዘዝ**

1. **ክፈለ-ጊዜ ሁለት የሚሰጠዉ በሁለተኛ ቀጠሮ ነዉ**
2. **የተሰጠ ሰዓት: 40 ደቂቃ**
3. **የክፍለ-ጊዜው ዋና-ዋና ግቦች**

- በእርግዝና ወቅት የሚደርሰዉ ጥቃት በእናቶች ላይ የሚያስከትለዉ መዘዝ
- ጥንዶችን በእርግዝና ወቅት የሚከሰት ጥቃት ማህበራዊና ኢኮኖሚያዊ መዘዞችን ለማሳወቅ
- በእርግዝና ወቅት የባሎች ሚና ምን እንደሆነ እንዲያዉቁ ማስቻል

1. **ክንዉኖች**

**የስራዎች ቀቅደም ተከተል**

1. ወደሁለተኛ ትምህርት ክፍለ-ጊዜ እንኳን ዳህና መጣችሁ ብሎ መጀመር
2. የቤት ስራዎች ላይ ማብራሪያ መስጠት

የዕለቱን ት/ት ክፍለ ጊዜ ማሳወቅ፡ ዛሬ የምንማረዉ በእርግዝና ወቅት የሚከሰተዉ የትዳር አጋር ጥቃት የሚያመጣቸዉ. ትምህርቱን ለመምራት እዚህ እሆናለሁ። ሆኖም፣ የእርስዎ ተሳትፎም ወደፊት እንድንገፋ ያደርገናል። ይህን ክፍለ ጊዜ የሚያበለጽጉት እና በህይወቶ ውስጥ ትርጉም የሚሰጡት የእርስዎ አስተዋጽዖዎች ናቸው።

1. እንደ ማስታወሻ ደብተር፣ እስክሪብቶ እና ፖስተሮች ያሉ ቁሳቁሶችን ያዘጋጁ
2. በተሰጠው ርዕስ ላይ ትምህርቱን ጀምር
3. ለጥያቄዎች ጊዜ ስጡ ወይም ጥንዶቹ ተረድተው ወይም እንዳልተረዱ ጥያቄዎችን ይጠይቁ

ጥንዶቹ ከተጠየቁት ጥያቄዎች ቢያንስ 75 በመቶውን ምላሽ መስጠታቸውን ያረጋግጡ

ጥንዶች በአሰልጣኙ ለተነሱት ጥያቄዎች ቢያንስ 75 በመቶ ምላሽ ካልሰጡ፣ ክፍለ ጊዜውን ለሌላ ጊዜ ይድገሙት።

1. ቤት ውስጥ መልመጃውን ያስተዋውቁ ፣ ለሚቀጥለው ክፍለ ጊዜ ቀጠሮ እና ጥንዶች ለተሳትፏቸው እውቅና ይስጡ

**ክፍል 3፡ በእርግዝና ላይ የሚደርሰው ጥቃት መዘዙ ቀጥሏል።**

1. **የሶስተኛውን ክፍለ ጊዜ በሶስተኛ ግንኙነት ያቅርቡ**
2. **የሚመከር ጊዜ: 45 ደቂቃዎች**
3. **የክፍለ ጊዜ ዓላማዎች**

- በእርግዝና ወቅት ጥንዶች ከእርግዝና ጋር በተያያዙ መዘዞች ላይ ለማስቻል

1. **ክንዉኖች**
2. **የክኑዉን ቅደም ተከተል**
3. ጥንዶቹን ወደ 3ኛው የትምህርት ክፍለ ጊዜ ሞቅ ያለ አቀባበል አድርጉላቸው
4. ከጥንዶች ጋር የቤት ውስጥ ልምምዶችን በተመለከተ አስተያየቶች
5. ክፍለ-ጊዜውን ያስተዋውቁ: ዛሬ ከእርግዝና ጋር በተያያዙት በእርግዝና ወቅት የሚደርሱ ጥቃቶችን እንቀጥላለን. በተጨማሪም፣ በዚህ ክፍለ ጊዜ ስኬት ውስጥ የእርስዎ ተሳትፎ የላቀ ሚና አለው።
6. እንደ ማስታወሻ ደብተር፣ እስክሪብቶ እና ፖስተሮች ያሉ ቁሳቁሶችን ያዘጋጁ
7. በተሰጠው ርዕስ ላይ ትምህርቱን ጀምር

- ከእርግዝና ጋር የተያያዙ ሁከት ውጤቶች በቀጥታም ሆነ በተዘዋዋሪ
- የጥቃት ፈጻሚዎች ላይ የሚያሳድረው ተጽዕኖ
- የጥቃት ተጽእኖ በህብረተሰቡ ላይ
- በእናቶች ጤና አጠባበቅ ላይ የወንዶች ተሳትፎ አስፈላጊነት

1. ለጥያቄዎች ጊዜ ስጡ ወይም ጥንዶች ተረድተዋል ወይም አልተረዱም ጥንዶች ከተጠየቁት ጥያቄዎች ቢያንስ 75 በመቶ ምላሽ መስጠታቸውን ያረጋግጡ ጥንዶች በአሰልጣኙ ለተነሱት ቢያንስ 75 በመቶ ጥያቄዎች ምላሽ ካልሰጡ ፣ ክፍለ ጊዜውን ለሌላ ጊዜ ይድገሙት ።
2. ለሚቀጥለው ክፍለ ጊዜ ወደ ቤት የመሄድ ልምምድ እና ቀጠሮ ያስተዋውቁ እና ተሳታፊዎችን እውቅና ይስጡ

**ክፍለ4፡ ኃይል፣ ቁጥጥር እና የቅርብ አጋር ጥቃትProvide this session at fourth contact**

1. **ክፈለ-ጊዜ አራት የሚሰጠዉ በአራተኛ ቀጠሮ ነዉ**
2. **የሚመከር ጊዜ: 45 ደቂቃዎች**
3. **የክፍለ ጊዜ ዓላማዎች**

- በግንኙነት ውስጥ ያሉ ጥንዶች የሃይል እና የቁጥጥር ሚዛን እንዲኖራቸው ለማስቻል
- ጥንዶች በጥንዶች ግንኙነት ጤናማ የሃይል ሚዛን እንዲኖራቸው ለማስቻል
- በእርግዝና ወቅት የወንድ አጋሮች ሚና ለመወያየት

1. **ክንዉኖች**

**v. የክንዉኖች ቅደም ተከተል**

1. ጥንዶችን ወደ 4ኛው የትምህርት ክፍለ ጊዜ በአክብሮት እንኳን ደህና መጣችሁ
2. ከጥንዶች ጋር በቤት ውስጥ በሚደረጉ ልምምዶች ላይ ነጸብራቆች
3. ክፍለ-ጊዜውን ያስተዋውቁ: ዛሬ በግንኙነት ውስጥ የኃይል እና የቁጥጥር ሚዛን, በጥንዶች መካከል ጤናማ ግንኙነት እንዴት እንደሚፈጠር እና በእርግዝና ወቅት የወንድ አጋሮች ሚና ላይ እንቀጥላለን.
4. እንደ ማስታወሻ ደብተር ፣ እስክሪብቶ እና ፖስተር ያሉ ቁሳቁሶችን ለንግግር ምቹ ቦታ ያዘጋጁ
5. በተሰጠው ርዕስ ላይ ትምህርቱን ጀምር

- በግንኙነት ውስጥ የኃይል እና ቁጥጥር ሚዛን እንዴት
- በጥንዶች መካከል ጤናማ ግንኙነት መፍጠር እንደሚቻል
- በእርግዝና ወቅት የወንድ አጋሮች ሚና

1. ለጥያቄ ጊዜ ስጡ ወይም ጥንዶች ተረድተዋል ወይም አልተረዱም

- ጥንዶች ከተጠየቁት ጥያቄዎች ቢያንስ 75 በመቶውን ምላሽ መስጠታቸውን ያረጋግጡ
- ጥንዶች በአሰልጣኙ ለተነሱት ጥያቄዎች ቢያንስ 75 በመቶውን ምላሽ ካልሰጡ፣ ለሌላ ጊዜ ይድገሙት።

1. ወደ ቤት የመውሰድ መልመጃውን ያስተዋውቁ፣ ለቀጣዩ ክፍለ ጊዜ ቀጠሮ ይያዙ እና ተሳታፊዎችን እውቅና ይስጡ

**ክፍል 5፡ የጓደኛ ጥቃት ቀስቅሴዎች**

1. **ይህ ክፍለ-ጊዜ የሚሰጠዉ በአምስተኛ ግኑኝነት ወቅት ነዉ**
2. **የተሰጠዉ ሰዓት፡ 40 ደቂቃ**
3. **የክፍለ -ጊዜ አላማዎች**

- በጋብቻ ህብረት ውስጥ ስላለው የጤና ግንኙነት ለመወያየት
- በግንኙነት ውስጥ ስላለው የኃይል እና የቁጥጥር ሚዛን ለመወያየት
- በእርግዝና ወቅት የወንዶችን ሚና ለማብራራት

1. **ክንዉኖች**

**የክንዉኖች ቅደም ተከተል**

1. ወደ 5ኛው የትምህርት ክፍለ ጊዜ ጥንዶችን በአክብሮት እንኳን ደህና መጣችሁ ብሎ መቀብል
2. ከጥንዶች ጋር ወደ ቤት ውሰዱ ላይ ያሉ አስተያየቶች
3. የእለቱን ትምህርት ማብራሪያ ስጥ
4. እንደ ማስታወሻ ደብተር፣ እስክሪብቶ እና ፖስተሮች ያሉ ቁሳቁሶችን ያዘጋጁ
5. በተሰጠው ርዕስ ላይ ትምህርቱን ጀምር

- የታወቁ የቅርብ አጋር ጥቃት ቀስቅሴዎች
- በትዳር ውስጥ ጤናማ ግንኙነት
- በእርግዝና ወቅት የወንድ አጋሮች ሚና

1. ለጥያቄዎች ጊዜ ስጡ ወይም ጥንዶቹ ተረድተው ወይም እንዳልተረዱ ጥያቄዎችን ይጠይቁ

- ጥንዶች ከተጠየቁት ጥያቄዎች ቢያንስ 75 በመቶውን ምላሽ መስጠታቸውን ያረጋግጡ
- ጥንዶች በአሰልጣኙ ለተነሱት ጥያቄዎች ቢያንስ 75 በመቶውን ምላሽ ካልሰጡ፣ ለሌላ ጊዜ ይድገሙት።

**ክፍል 6: ሴቶችን ማብቃት**

- - 1. ክፈለ-ጊዜ የሚሰጠዉ በስድስተኛ ቀጠሮ ነዉ
    2. የሚመከር ጊዜ: 45 ደቂቃዎች
    3. የክፍለ ጊዜ ዓላማዎች
  - ከጥንዶች ጋር ስለሴቶች ማብቃት ጽንሰ-ሀሳቦችን ማወቅ
  - በእርግዝና ወቅት አይፒቪን በመከላከል ረገድ ጥንዶችን በሴቶች ማጎልበት አስፈላጊነት ማበልጸግ ።
    1. ክንዉኖች
    2. የክንዉኖች ቅደም-ተከተል

1. ጥንዶቹን ወደ 6ኛው የትምህርት ክፍለ ጊዜ ሞቅ ያለ አቀባበል አድርጉላቸው.
2. ክፍለ ጊዜውን አስተዋውቁ:
3. እንደ ማስታወሻ ደብተር ፣ እስክሪብቶ እና ፖስተር ያሉ ቁሳቁሶችን ያዘጋጁ
4. በተሰጠው ርዕስ ላይ ትምህርቱን ጀምር

- ሴቶችን ማብቃት
- በእርግዝና ወቅት የቅርብ ወዳጃዊ ጥቃት የሴቶችን ማብቃት አስፈላጊነት

1. ለጥያቄዎች ጊዜ ስጡ ወይም ጥንዶቹ ተረድተው ወይም እንዳልተረዱ ጥያቄዎችን ይጠይቁ

- ጥንዶች ከተጠየቁት ጥያቄዎች ቢያንስ 75 በመቶውን ምላሽ መስጠታቸውን ያረጋግጡ
- ጥንዶች በአሰልጣኙ ለተነሱት ጥያቄዎች ቢያንስ 75 በመቶውን ምላሽ ካልሰጡ፣ ለሌላ ጊዜ ይድገሙት።

1. መዝጊያ

ከተሳታፊዎች የተሰጠ አስተያየት

ተሳታፊዎችን ማመስገን

**መጨራሻ!**

**Annex-ix: ጥንዶችን መሰረት ያደረገ ጥቃት መከላከል ትምህርት-ማስተማሪ**

**የእናትነት ጤና**

- የእናትነት ጤና ማለት የእናቶች ጤናማነት በእርግዝና፣ ወሊድና ድህረ-ወሊድ ወቅት ማለት ነዉ
- ይህም የሚሆነዉ የቅድመ-ወሊድ ክትትል ማድረግ፣ ጤና ተቋም መዉለድ እና ድህረ- ወሊድ ክትትል በማድረግ ነዉ፡፡
- **ጸታን መሰረት ያደረገ ጥቃት(ጾታዊ ጥቃት)**
- ማንንኛዉም ጾታን መሰረት ያደረገ(ሴት በመሆነዋ፣ ወንድ በመሆኑ ጥቃት ጾታን መሰረት ያደረገ ጥቃትይባላል፡፡
- የሚከሰተዉም በጥቃት ፈጻሚዉና ተጎጂ መካካከል ያለዉ ያልተመጣጠነ ግኑግነት ነዉ፡፡

**ሴቶች ላይ የሚፈጸም ጥቃት**

- በሴቶች ላይ የሚፈጸም ጥቃት ማለት ማንኛዉም ጾታን መሰረት ያደረገ ጥቃት ስሆን በሴቶች ላይ ጥቃት ሲሆን፡-
  - - አካለዊ ጥቃት
    - ወሲባዊ ጥቃት
    - ስነልቦናዊ ጥቃት
    - ስቃይ ,
    - ማስፈራራት ናቸዉ

**የቅርብ አጋር ጥቃት**

የቅረብ አጋር ጥቃት ማለት ማንኛዉም በቅርብ ግኑኝነት የሚፈጠር ባህሪ ሲሆን አካላዊ ፣ስነልቦናዊ ወይም ወሲባዊ ጉዳት በቅርብ ጓደኛ ላይ ማድረስ ነዉ፡፡

የቅርብ አጋር/ባል/ጥቃት በሴቶች ላይ ከሚፈጸሙ ጥቃቶች የአንበሳዉን ድርሻ ይወስዳል፡፡

የቅርብ አጋር ጥቃት በሚከተሉት መልክ ልገለጽ ይችላል

- አካላዊ,
- ወሰ,
- ሥነ ልቦናዊ ወይም ስሜታዊ,

**አካላዊ የቅርብ አጋር ጥቃቶች**

- በጥፊ መምታት ወይም አጋርን ሊጎዳ የሚችል ነገር መወርወር
- አጋርን መግፋት
- በሆድ መምታት
- ባልደረባን መግፋት ወይም የባልደረባን ፀጉር መሳብ፣
- በእጁ ወይም በሌላ አጋርን ሊጎዳ በሚችል ነገር መምታት
- ምታ፣
- አጋርን መጎተት ወይም መምታት
- ሆን ብሎ ለማፈን ወይም ለማቃጠል መሞከር
- በባልደረባ ላይ ሽጉጥ፣ ቢላዋ ወይም ሌላ መሳሪያ ለመጠቀም ወይም ለመጠቀም ማስፈራራት

**የወሲብ የቅርብ አጋር ጥቃት ዉስጥ የሚካተቱት**

- ባልፈለገች ወይም ባልፈለገች ጊዜ በግብረ ሥጋ ግንኙነት እንድትፈጽም አስገድደው ወይም
- ባልደረባውን በማስፈራራት ወይም በማናቸውም ሌላ መንገድ እሷ/እሷ የማትፈልገውን ወይም ያልፈለገችውን ወሲባዊ ድርጊቶችን እንዲፈትፈጽም ማስገደድ ወይም
- ባልደረባው/እሷ የማትፈልጋቸውን ሌሎች ወሲባዊ ድርጊቶችን እንዲፈጽም በአካል ማስገደድ

**ስነ ልቦናዊ ወይም ስሜታዊ የቅርብ አጋር ጥቃቶች**

- ባልደረባው እሷን/እሱን በሌላ ሰው ፊት ለማዋረድ አንድ ነገር መናገር ወይም ማድረግ ወይም
- የሕመምተኛውን/እህትን ወይም የምትጨነቅበትን/የምትፈልገውን ሰው ለመጉዳት ወይም ለመጉዳት ማስፈራራት
- መሳደብ ወይም እሷ/እሷ ስለራሷ/እሷ መጥፎ ስሜት እንዲሰማቸው ማድረግ

**ክፍል -ሁለት**

**በእረግዝና ወቅት የቅርብ አጋር ጥቃት መዘዞች**

**በእናቶች ጤና ላይ የሚያመጠዉ መዘዝ**

- ጉደት(የአጥንት መሰበር, መቃጠል, መቆረጥ, ጥርስ መሰበር/መርገፍ, እጅና እግር መሰበር)
- አካል መጉደል
- ለደወለደዉ ህጻን ፍቅር አለመኖር
- ለአባላዘርና ስነተዋልዶ ጤና ችግር መጋለጥ (ኤች አይ ቪን ጨምሮ)
- የአካል ጉዳት
- ነፍስ መግደል
- በእርግዝና ወቅት ደም መፍሰስ
- የእናቶች ሞት

***ስነ-ልቦናዊ***

*ቀጥተኛ ተጽኖ፡ስጋት፣ ፍርሃት፣ ሌሎችን አለማመን፣ ትኩረት አለመስጠት፣ ብቸኝነት ስሜት፣ ድብርት ፣ እራስን ማጥፋ፣ነፍስ ማጥፋት ወዘተ..*

***ባህሪያዊ***

- ብዙ የሴት ጓደኛ መኖር
- በእረግዝና ወቅት አስካሪ መጠጥ/አልኮል መዉሰድ
- በእርግዝና ወቅት ማጨስ
- ጫት መቃም
- በግዜ የቅድመ-ወሊድ ክትትል አለማድረግ

***ኢኮኖሚያዊና ማህበራዊ መዘዞች***

- አለመቀበል፣እራስን ማግለል እና ማህበራዊ መገለል
- አነስተኛ የሆነ ማህበራዊና ኢኮኖሚያዊ ተሳትፎ
- ወደፊት ጥቃት ይደርስብኛል ብሎ ማሰብ ከግለሰብ አልፎ በማህበረሰብ ላይ
- የሴቶች ራስ መተማመን መቀነስ
- ለሌሎች ጾታዊ ጥቃት ተጋላች መሆን
- ስራ ማጣት ብዙ ጊዜ በጥቃት ምክንያት በመቅረት
- ሴቶች ገቢ እንዳያገኑ ማድረግ

***በቤተሰብ ላይ የሚያመጠዉ ተጽኖ***

- ፍቺ ወይም ቤተሰብ መበተን
- የቤተሰብ ኢኮኖምያዊና ስነል-ቦናዊ አደጋ ላይ መጣል
- ልጅ ያለ -ጊዜ መወለድ ወይም ከመጠን በታች ክብደት ያለዉ ልጅ መወለድ
- የበት ዉስጥ ጥቃት ባለበት ልጅ ሲያድግ በጥቃት ማደግ እድሉ ከፍተኛ ነዉ

**ክፍል-3**

**ጥቃት በእርግዝና ላይ የሚያመጣቸዉ መዘዝ**

- በሆድ ዉስጥ ያለዉ ህጻን የእድት ዉስንነት የማጋጠም ዕድሉ ከፍተኛ ነዉ
- ያለጊዜ ሚጥ የመጀመር እድሉ ከፍተኛ ነዉ
- ጽንስ ያለመቀጠል ሁኔታ ይጨምራል
- አግባብ ባልሆነ መንገድ ጽንስ የማቋረጥ እድሉ ይጨምራል
- ህይወት የለለ ልጅ የመወለድ እድል ይጨምራል
- ህጻን ከ28 ሳምንት እስከተወለደ 7 ቀን ዉስጥ የመምት እድል ይቸምራል
- ክብደቱ ያነሰ ልጅ የመወለድ ዕድሉ ይቸምራል
- በቂ ያለሆነ ክብደት መቸመር
- ለአባላዘር ወይም ለኤች አይ ቪ የመጋለጥ እድሉ ይቸምራል
- ህጻን ከተወለደ 28 ቀን ዉስጥ የመሞት እድሉ ይቸምራል

***በጥቃት ፈጻምዎች ላይ የሚያመጣ ተጽኖ***

- በማህበረሰብ ዉስጥ እንዳይሳተፍ ይደረጋል
- ለእስር ያደርጋል
- ቤተሰቦቹን እንዳይገናኝ ይደረጋል
- ፍቺ ወይም ቤተሰብ መበተን
- ለቤተሰብ ባይታዋር መሆን
- ቤት ዉስጥ ዉጥረትነ ፍርሃት እንድኖር ያደርጋል

***በህብረተሰብ ላይ የሚያመጠዉ መዘዝ***

- በጤና ስርዓትና ፍትህ ስርኣት ላይ ተጽኖ ያደር
- በጠፋው የሴቶች ምርታማነት የኢኮኖሚ መረጋጋትና እድገት ማደናቀፍ
- ሴቶች በልማት ሂደት ውስጥ የሚኖራቸው ተሳትፎ እና ለማህበራዊ እና ኢኮኖሚያዊ እድገት የሚያበረክቱትን አስተዋፅኦ መቀነስ
- የሴቶች ፈጣን ማህበራዊ፣ፖለቲካዊ ወይም ኢኮኖሚያዊ ለውጥ ምላሽ የመስጠት አቅም ውስንነት
- በማህበራዊ ግንኙነቶች ላይ እምነት መፈራረስ
- የሰዎች የመዳን ስልቶች የተመካባቸው የተዳከሙ የድጋፍ መረቦች
- በጭንቀት እና በግርግር ጊዜ የማህበረሰቡን አቅም ለማጠናከር ወሳኝ ጠቀሜታ ያላቸው የተጣሩ እና የተበታተኑ አውታረ መረቦች

**በእናቶች ጤና ክብካቤ (ቅድመ ወሊድ፣ ወሊድ እና ድህረ ወሊድ) የወንድ ተሳትፎ አስፈላጊነት**

- በቅድመ ወሊድ እንክብካቤ፣በተቋማት መዉለድ እና የድህረ ወሊድ ምርመራ/ አስፈላጊነት መረጃን ከጤና አጠባበቅ ሰጭዎች ያገኛል
- የትዳር ጓደኛውን (ባለቤቱን) የቅድመ ወሊድ ቀጠሮዎችን ያስታውሳል
- የቅድመ ወሊድ ምርመራ ለማድረግ ወደ ሆስፒታል ወይም ጤና ጣቢያ የሚሄዱትን ገንዘብ ጨምሮ መጓጓዣን ያዘጋጃል
- በእርግዝና ወቅት ከእናቶች የስራ ጫና ጋር በተያያዘ ጥቅማ ጥቅሞችን ያስገኛል፣
- ጥንዶች ለወሊድ ዝግጁነት እና ውስብስብነት ዝግጁነት፣
- በድህረ-ወሊድ ክትትል፣
- በእርግዝና ወቅት የባል ግንኙነትን እና ስሜታዊ ድጋፍን ለሴቶች ያስችላል
- ጡት ማጥባትን ጨምሮ የህጻናት አመጋገብን ማሻሻል
- የህፃናት የክትባት ሽፋንን ያሻሽላል
- ከወሊድ በኋላ የወሊድ መከላከያ አጠቃቀምን ያሻሽላል።

**የቤት ስራ**

**በሚከተሉት ተግባራት የመጨረሻ ውሳኔ የሚሰጠው ማነው?**

የቤት ውስጥ ግዢ ከትናንሽ እና ከትልቅ፣•

ዘመዶች እና ጓደኞች መጎብኘት

የሚስትን ገቢ ማውጣት

የሚወልዷቸው ልጆች ብዛት

ለራስዎ የጤና አገልግሎት ማግኘት

**ክፍል-አራት**

***በትዳር ዉስጥ የሀይል ሚዛን ውስጥ ኃይል***

- በግንኙነት ውስጥ ያለው ሃይል ጥንዶች እንዲቆጣጠሩ፣ እንዲመርጡ እና በወቅታዊ ሁኔታዎች ላይ ተጽእኖ እንዲያሳድሩ የሚያስችል አቅም እንዲኖራቸው ያደርጋል።
- በትዳር ግንኙነት ወቅት የስልጣን ማነስ በውሳኔአችን ወይም በዕጣ ፈንታችን ላይ ቁጥጥር እንዳንሆን የማያቋርጥ ማሳሰቢያ ነው

**የጋራ ኃይል/ሚዛናዊ ግኑግነት**

- የሚከሰተው አጋሮች ስለራሳቸው ግምት እና በራስ የመመራት ችሎታ ሲያውቁ እና ሲተማመኑ ነው

**ኃይል ግንኙነቱን ተጽኖ**

- ጠንካራ እና ጤናማ በሆኑ ግንኙነቶች የሁለቱም አጋሮች ተጽእኖ ከሞላ ጎደል እኩል ነው.
- አንዱ የበለጠ የገንዘብ ሃይል ሊኖረው ይችላል, ሌላኛው ደግሞ የበለጠ ማህበራዊ ግንኙነቶች, ግን በመጨረሻም እርስ በርስ ይከባበራሉ እና ውሳኔዎችን ያደርጋሉ.

**በትዳር ውስጥ የኃይል አለመመጣጠን ውጤቶች**

- የተበላሸ ቅርበት እና ግንኙነት
- የቅርብ አጋሮች አካላዊ፣ፆታዊ እና ስነ ልቦናዊ ጥቃት
- ብስጭት፣ ቁጣ እና ድብርት
- የጭንቀት፣ፍርሃት እና እፍረት ስሜት
- የራስ ግምት መጓደል፣የራስን አመለካከት እና የሰውን ስሜት ማግለል
- መነጠል፣ዛቻ እና ማጎሳቆል
- በባልደረባ ላይ እምነት ማጣት እና የግንኙነቱ ጽናት
- በግንኙነት አጠቃላይ እርካታን መቀነስ
- ግንኙነት ወይም ጋብቻ መጨረሻ/ፍቺ/መለያየት

**በጥንዶች ግንኙነት ውስጥ ጤናማ የኃይል ሚዛን እንዴት እንደሚኖር**

1. **መከባበር**

- መከባበር እና መተማመን የየትኛውም ጠንካራ ግንኙነት መሰረት ናቸው
- የባልደረባዎን አስተያየት ያክብሩ2.

1. የገንዘብ ውሳኔዎችን አንድ ላይ ያድርጉ

- ብዙውን ጊዜ በግንኙነት ውስጥ ያለው የኃይል ሚዛን የሚወሰነው በገንዘብ ነው
- ጥንዶች በገንዘብ ወጪ ላይ አብረው መወሰን

1. **ጥሩ የሐሳብ ልውውጥን ማዳበር**

- በግንኙነት ውስጥ ጤናማ ያልሆነ ወይም ሚዛናዊ ያልሆነ የሃይል ለውጥ ከሚያሳዩት ምልክቶች አንዱ በትዳር ጓደኞች መካከል አለመነጋገር ነው
- ሁለቱም አጋሮች ችግሮችን ያለፍርሃት የመፍታት ነፃነት ሊኖራቸው ይገባል
- ሃሳብዎን የመናገር ነፃነት ለጤናማ ግንኙነት ቁልፍ መሆኑን ማወቅ

1. **በራስ መተማመንን መቸመር**

- የአንዱ አጋር በራስ መተማመን ማጣት ወይም ለራስ ያለው ግምት ማነስ የሃይል ሚዛን መዛባትን ያስከትላል
- በራስ ቀዳሚ ስራ ላይ መስራት
- ከራስዎ ጋር ጤናማ ግንኙነት መፍጠር፣ፍላጎትዎን በግልፅ እና በብቃት ማሳወቅን ይማሩ

1. **ጥንዶች የግንኙነት ፍላጎቶችን ለማሟላት መጣር**

- ግንኙነቶች ሁሉም ነገር መስጠት እና መውሰድ
- የእራስዎን እና የትዳር አጋርዎን ማወቅ ለተመጣጠነ እና ጤናማ ግንኙነት ቁልፍ ነው

**እርግዝና ወቅት የባል/የወንድ አጋር ሚና**

**"እርግዝና ለእያንዳንዱ ሴት ሕይወትን የሚለውጥ ደረጃ ነው"**

**" ሚስት በባልዋ የምትደገፍ ሴት ደስተኛ ትሆናለች እና ጭንቀት ይቀንሳል"”**

1. **በሁሉም የሕክምና ቀጠሮዎች ጊዜ ከሚስትዎ ጋር አብረው መሄድ**

- የቅድመ ወሊድ እንክብካቤ
- በወሊድ ጊዜ
- የድህረ ወሊድ ምርመራ አብሮ መሔድ
- ይህም ባልደረባው የሚስቱን እና የልጁን የጤና ሁኔታ እንዲከታተል ያስችለዋል,
- ለሴቶች የሚሰጠውን ስሜታዊ ድጋፍ ማጠናከር

1. **በቤት ውስጥ ሥራዎች ውስጥ መሳተፍ**

- ሚስትህ በምትፈልገው የለውጥ ሂደት ውስጥ እንደምትገኝ ተረዳ

እረፍት

ድጋፍ

ብዙ ግንዛቤ

1. **ከባለቤትዎ/ባልደረባዎ ጋር መነጋጋር**

- እሷ ያለባትን ማንኛውንም ጭንቀት ለመለየት በጣም ጠቃሚ ነው ሚስትዎን ለመደገፍ በር ይከፍታል

1. **የልደት እቅድ ያዘጋጁ**

- የገንዘብ ዝግጅት
- ለመጓጓዣ ተዘጋጁ
- ወዴት እንደሚወልዱ ያቅዱ

1. **የሚስትን ስሜት መረዳት**

- በእርግዝና ወቅት የሚደረጉ የሆርሞን ለውጦች ምቾት ማጣት፣ ድካም እና ድካም ስሜት ሊሰማቸው ይችላል፣
- አንድ ሰከንድ ላይ ደስተኛ ልትሆን ትችላለች፣ በሌላ ጊዜም ቢሆን ምናልባት ስሜቷን መረዳት ።

1. አመጋገቧን ማስተካከል

- በቂ ምግብ እየተመገበች እንደሆነ ማረጋገጥ
- ጤናማ የሆኑ ምግቦችን እንድትጠቀም ማበረታታት

1. **በቂ እረፍትና እንቅልፍ እነድቶስድ ማድረግ**
2. **ምስትህን ደንብ ማዳመጥ**
3. **አኗኗሯን ማስተካካል**

- በጣም ብዙ ቡና እንደትጠጣ መከልከል
- በአጠገቧ አላማጫስ
- አስካሪ መጠጥ አለመዉሰድ

1. **የተወሰነ እግር ጉዞ እንደ መዝናኛ መሄድ**

የቤት ስራ/ተወያዩ

- የቅርብ አጋር ጥቃትን በእርግዝና ወቅት የሚያበባብሱ ነገሮች ምንድናቸዉ?
- በቤት ዉስጥ አላመግበባት ብፈጠር እንዴት ትፈታላችሁ?

**ክፍል-5**

**የቅርብ-አጋር ጥቃት እነድከሰት መንስኤዎች**

- አስካሪ መጠጦችን መዉሰድ
- የልተፈቀዱ መድኃኒቶችን መዉሰድ
- ተማኝነት ማጣትና መጠርጠር
- የአእምሮ ህመም (ድብርት , ጭንቀት )
- ጠርጣሬ

በእርግዝና ወቅት የሚከሰቱ ጥቃቶችን እንዴት መከላከል/መቅረፍ ይቻላል

አስካሊ መጠጥ አለመዉሰድ ወይም ካልተቻለ መቀነስ

- ነገሮችን በመነጋገር መፍታት
- ባል ወይም ምስት ጥርጣሬ ወይም የተዛባ መረጃ ካሌ፣በሱ ላይ መነጋገር፣ ተረጋግቶ መናገር፣ በግልጽና በመተማመን
- በነገሮች ላይ ጉልበት አለመጠቀም
- የራስን እምነትና አመላካከት በሌሎች ላይ መጫን ጤናማ አይደለም
- ለግጭት የሚዳርጉ ነገሮችን ወዴ ጠረጴዛ ዙሪያ ማምጣትና መነጋገር
- አለመዉቀስ/መተቸት
- ለጤናማ ግኑግነት ቅድሚያ መስጠት
- ለትደር ጓደኛ ታማኝ መሆን
- አለመዋሸት
- ያለፈዉን ቁስል/ስህተት አለማንሳት
- የትዳር አጋርን ድንበር ማክበር
- ተጠራጣሪ አለመሆን
- ጥሩ ሰሚ መሆን

**ክፍል -6**

**ሴቶችን ማብቃት**

ማብቃት ማለት፡ ማብቃት ማለት ሴቶች ሆኑ ወንዶች የራሳችዉን ህይወት በራሳቸዉ መቆጣጠር ስችሉ ሲሆን የራሳቸዉን አጀንዳ መወሰን፣ ክህሎት መቅሰም (የራሳችዉ ክህሎትና እዉቀት መገንዘብ)፣ የራስ መተማመን መጨመር፣ ችግሮችን መፍታት ናቸዉ

**ሴቶችን ማብቃት** ማለት፡ሴቶች በማሀበራዊ ፣ ፖሎቲካዊና እና ኢኮኖሚያዊ ህይወት ላይ ሙሉ በሙሉ መሳተፍ ስችሉ ነዉ

**ሴቶችን ማብቃት የሚያስገኙ ጥቅሞች በጥቂቱ**

- ጥቃቶች አይኖሩም ወይም ይቀንሳል
- ስነ-ወሲባዊና ስነ-ተዋልዶ ጤና ላይ የራሳቸዉ መብት እንድኖር ያደርጋል
  - የሴቶች ድምጽ ይሰማል
  - ማህበራዊና ኢኮኖሚያዊ እንቅስቃሴን ያፈጥናል
  - ሴቶች ገብያቸዉን በራሳቸዉ እንድቆጣጠሩ ያደርጋል

**ሴቶችን ማብቂያ መንገዶች**

- በሁሉም ሴቶች እና ልጃገረዶች ላይ የሚደርሰውን ማንኛውንም አይነት መድልዎ በማቆም፣
- በሁሉም ሴቶች እና ልጃገረዶች ላይ የሚደርሰውን ማንኛውንም አይነት ጥቃት እና በመንግስት እና በግሉ ዘርፍ፣ህገወጥ የሰዎች ማዘዋወር፣ ወሲባዊ እና ሌሎች ብዝበዛን በማሰወገድ
- እንደ ልጅ፣ ያለ እድሜ እና አስገዳጅ ጋብቻ እና የሴት ልጅ ግርዛትን የመሳሰሉ ጎጂ ልማዶችን ማስወገድ.
- ያልተከፈለ እንክብካቤን እና የቤት ውስጥ ሥራን በሕዝብ አገልግሎቶች አቅርቦት ፣ በመሠረተ ልማት እና በማህበራዊ ጥበቃ ፖሊሲዎች እና በቤተሰብ እና በቤተሰብ መካከል የጋራ ኃላፊነትን በማሳደግ እውቅና እና ዋጋ መስጠት
- በፖለቲካዊ፣ ኢኮኖሚያዊ እና ህዝባዊ ህይወት ውስጥ በሁሉም የውሳኔ ሰጪነት ደረጃዎች የሴቶችን ሙሉ እና ውጤታማ ተሳትፎ እና የአመራር እኩል እድል ማረጋገጥ
- ሴቶች በኢኮኖሚያዊ ሀብቶች እኩል መብት እንዲኖራቸው፣ እንዲሁም በመሬትና በሌሎች የንብረት ዓይነቶች፣ የፋይናንስ አገልግሎቶች ባለቤትነት እና ቁጥጥር እንዲያገኙ ማሻሻያዎችን ማድረግ፣
- የሴቶች ትምህርት አገልግሎትን ማስፋፋት
- ብቻል የራሳቸዉ ገቢ እንድኖር ማድረግ

**CURRICULUM VITAE- ZELEKE DUTAMO AGDE**

**PERSONAL INFORMATION**

- Full name: Zeleke Dutamo Agde
- Department of Reproductive health, College of Medicine and Health Sciences, Wachemo University, Hossana, Ethiopia

**Education**

Year

2020-now PhD Fellow, Jimma University, Institute of Health, Jimma, Ethiopia

2012-2014 MPH/RH, Haramaya University, College of medical and Health Sciences

2007-2010 BSc Nursing, Addis Ababa University, Addis Ababa, Ethiopia

**Academic experience**

2010-2012 Assistant Lecturer, Samara University, Department of Nursing,

2015-20176 Lecturer in Reproductive Health in Samara University, Department

Of Nursing, Samara, Ethiopia

2017-2019 Lecturer in Reproductive Health, Wachemo University, Department of Public Health

2020-now PhD fellow, Jimma University, Institute of Health, Jimma, Ethiopia

**Leadership positions**

2010-2011 I worked as Nursing department head, Samara University, Ethiopia

2018-2019 I worked as post graduate program coordinator of college of medicine and Health sciences, Wachemo University, Ethiopia

**Language skills**

| **Language** | **Listening** | **Speaking** | | **Reading** | **Writing** |
| --- | --- | --- | --- | --- | --- |
| Hadiyisal (local language) | Excellent | | Excellent | Excellent | Excellent |
| Amharic | Excellent | Excellent | | Excellent | Excellent |
| English | Very Good | Very Good | | Excellent | Excellent |

**Training and conferences attended**

1. Palliative care from July5-10, 2010 at Zewditu Hospital, Addis Ababa ,Ethiopia

2. STI/PEP from July 2-4, 2010 at Zewditu Hospital, Addis Ababa, Ethiopia

3. PMTCT from July 13-16, 2010 at Zewditu Hospital, Addis Ababa, Ethiopia

4. PICT from July 9-12, 2010 at Zewditu Hospital, Addis Ababa, Ethiopia

5. TB/HIV from July 19-22, 2010 at Zewditu Hospital, Addis Ababa, Ethiopia

6. IMNCI from February 04-09, 2012 at Dubti Hospital, Afar, Ethiopia

7. Syndromic management of STI, Addis Ababa, Ethiopia

8. Attended early conference of Ethiopian Public Health Associations 2015-2022

9. The international basic clinical ethics course from May 9-12, 2023, Oslo, Norway

10. Attended 7th National PhD Conference in Global Health, organized by NRSGH – Norwegian Research School of Global Health at Reine Rorbuer, Lofoten, Norway from 24 – 27 April 2023

**Publications**

1. **Zeleke Dutamo**, Nega Assefa2 and Gudina Egata. Maternal health care use among married women in Hossaina, Ethiopia. Dutamo et al. BMC Health Services Research (2015) 15:365. DOI 10.1186/s12913-015-1047-1

2. **Zeleke Dutamo**, Skilled Delivery Care Utilization among Currently Married Women of Reproductive Age in Hossana, Southwest Ethiopia . Journal of Pregnancy and Child Health Journal of Pregnancy and Child Health. **ISSN: 2376-127X** Dutamo et al., J Preg Child Health 2015, 2:3: <http://dx.doi.org/10.4172/2376-127X.1000162>

3. Belachew Melese Hunde, **Zeleke Dutamo Agde**. Statistical Analysis of Road Traffic Car Accident in Dire Dawa Administrative City, Eastern Ethiopia. Science Journal of Applied Mathematics and Statistics 2015; 3(6): 250-256. doi: 10.11648/j.sjams.20150306.14 ISSN: 2376-9491 (Print); ISSN: 2376-9513

4. Tekle G, **Dutamo Z** (2019). Survival Analysis of Determinants of Breast Cancer Patients at Hossana Queen Elleni Mohammad Memorial Referral Hospital, South Ethiopia: Bayesian Application of Hypertabastic Proportional Hazards Model. International Journal of Public Health and Epidemiology Research, 5(2): 108-118

**REFERENCE**

- Nega Assefa (PhD, professor) –College of health and medical sciences, Haramaya University, Ethiopia
- Gudina Egata(PhD,associate professor)- College of health and medical sciences, Haramaya University, Ethiopia
