# Supplementary material for: The protocol for a cluster randomized controlled trial to evaluate couple-based violence prevention education and its ability to reduce intimate partner violence during pregnancy in Southwest Ethiopia
Source: PLoS One. 2024 May 13;19(5):e0303009. doi: 10.1371/journal.pone.0303009 (PMC11090299; doi:10.1371/journal.pone.0303009)
Supplement: Attachment — Submitted filename: Plos_one_23_review.pdf [file pone.0303009.s006.pdf]

This paper presents a protocol for a randomized controlled trial analyzing an intervention targeting IPV among couples including a pregnant woman in Ethiopia. The trial seems valuable in terms of its learning potential, but I have a number of comments.

First, the protocol does not seem well-situated in the literature at all. There are a number of recent papers analyzing couples' interventions targeting IPV, including one in Ethiopia, that are not cited here.

- For a couples' intervention in Ethiopia: Sharma V, Leight J, Verani F, Tewolde S, Deyessa N (2020) Effectiveness of a culturally appropriate intervention to prevent intimate partner violence and HIV transmission among men, women, and couples in rural Ethiopia: Findings from a cluster-randomized controlled trial. PLOS Medicine 17(8): e1003274.
- For a couples' intervention in Rwanda: Dunkle K, Stern E, Chatterji S, et al. Effective prevention of intimate partner violence through couples training: a randomised controlled trial of Indashyikirwa in Rwanda. BMJ Global Health 2020;5:e002439.
- A recent systematic review (that encompasses both of the above): Leight J, Cullen C, Ranganathan M, Yakubovich A. Effectiveness of community mobilisation and group-based interventions for preventing intimate partner violence against women in low- and middle-income countries: A systematic review and meta-analysis. J Glob Health. 2023 Oct 20;13:04115.

The protocol should provide a better overview of the existing literature and clarify what the incremental contribution of this paper is and how the theory of change relates to these existing papers and is suggestive of potential effectiveness.

Second, I would like to hear more about how the implementers will ensure implementation fidelity given that they rely on HEWs for implementation, and there is already substantial evidence that HEWs do not execute their core responsibilities effectively given a high volume of work. See for example: Warren, A. M., and Frongillo, E. A. (2017). Mid-level actors and their operating environments for implementing nutrition-sensitive programming in Ethiopia. Global Food Security, 13, 66–73. How can they be sure that HEWs will deliver the planned intervention?

Third, sample and statistical power: in my view the sample is too small, and statistical power seems insufficient. The systematic review and meta-analysis cited above does not suggest that intervention effects of this size for IPV are observed for the average intervention. The authors should consider expanding the sample and if not feasible, have a longer discussion in the paper as to what limited statistical power implies for the potential of the evaluation. (Is statistical power higher for other outcomes?)

Fourth, motivation: why does the trial focus on IPV among pregnant women in particular, as opposed to IPV among women in general? Is it primarily because of the potential for adverse health effects among pregnant women (the current motivation in the introduction seems to suggest this)? Do you expect that pregnant women and their spouses may be uniquely receptive to interactions with HEWs or to behavioral change given the particular moment they are passing through related to pregnancy? Is any of the content uniquely tailored to the life stage of pregnancy (sounds like some of it, but perhaps not all of it)? Right now, the protocol reads more like an evaluation of an IPV

prevention intervention that just happens to be delivered to pregnant women, but it would be optimal to have more clarity about how this context matters.
