# Supplementary material for: The protocol for a cluster randomized controlled trial to evaluate couple-based violence prevention education and its ability to reduce intimate partner violence during pregnancy in Southwest Ethiopia
Source: PLoS One. 2024 May 13;19(5):e0303009. doi: 10.1371/journal.pone.0303009 (PMC11090299; doi:10.1371/journal.pone.0303009)
Supplement: Attachment — Submitted filename: Rebuttal letter.docx [file pone.0303009.s007.docx]

**Rebuttal Letter**

**Dear editor/s and reviewers;**

**Subject: Submission of revised manuscript ID: PONE-D-23-32195**

We hope this email finds you well. We would like to thank the editorial team and reviewers for their time and efforts to provide valuable feedback. All the comments and questions raised by the editor(s) and reviewers are really important to further improve our manuscript. We have carefully addressed all the comments and questions raised by the editorial team and reviewers, point by point. We highlighted it with a yellow color in the revised manuscript.

**Responses to editor(s)**

1. Comment: when submitting your revision, we need you to address these additional requirements. Please ensure that your manuscript meets PLOS ONE's style requirements, including those for file naming.

-We prepared according to PLOS ONE manuscript guideline including file naming

2. Comment: In your Data Availability statement, you have not specified where the minimal data set underlying the results described in your manuscript can be found.

-Not applicable

3. Comment: Please include your full ethics statement in the ‘Methods’ section of your manuscript file. In your statement, please include the full name of the IRB or ethics committee who approved or waived your study, as well as whether or not you obtained informed written or verbal consent. If consent was waived for your study, please include this information in your statement as well.

-We moved the ethics statement section into methods section and mentioned the full name of the IRB

**Responses to reviewers**

**Reviewer #1:**

Comment: this paper presents a protocol for a randomized controlled trial analyzing an intervention targeting IPV among couples including a pregnant woman in Ethiopia

-Thank you for your concern, dear reviewer. We modified the title in the revised manuscript.

Comment: here are a number of recent papers analyzing couples’ interventions targeting IPV, including one in Ethiopia, that are not cited here.

-Thank you for your concern, dear reviewer. We have rigorously reviewed the existing literatures and synthesized in the introduction part

Question: what the incremental contribution of this paper is?

-We addressed in the introduction section of the revised manuscript

Comment: how the theory of change relates to these existing papers and is suggestive of potential effectiveness

-We appreciate your suggestion, dear reviewer. Theory of change has not been mentioned explicitly as we have reviewed the existing papers. But, we have already adapted the ecological theory to effectively addresses the multilevel factors (individual, relational, community and societal) in the intervention which is not mutually exclusive from theory of change.

Comment: I would like to hear more about how the implementers will ensure implementation fidelity given that they rely on HEWs for implementation.

- Thank you for your concern, dear reviewer. We put strategies to ensure the intervention's fidelity in the revised manuscript. To minimize the workload, we have selected one HEW of the two HEWs in the selected cluster, and sessions will probably be held on weekends, which do not affect government working times.

Comment: sample and statistical power: in my view the sample is too small, and statistical power seems insufficient.

- Thank you for your concern, dear reviewer. It was beneficial to have a larger sample size to obtain more precise and powerful results. However, the feasibility concern limited our further improvement of the study’s power as this is a PhD project with a limited budget and no other sources of funding. We will address it as a limitation during the final study report.

Question: is statistical power higher for other outcomes?

-Thank you for your concern, dear reviewer. No, we have calculated for all outcomes and considered the larger sample size for our study.

Question: why does the trial focus on IPV among pregnant women in particular, as opposed to IPV among women in general? Is it primarily because of the potential for adverse health effects among pregnant women (the current motivation in the introduction seems to suggest this)

-Thank you for your concern, dear reviewer. Absolutely, it is primarily because of potential adverse maternal and newborn effects. We addressed this in the introduction section of the revised manuscript.

Question: do you expect that pregnant women and their spouses may be uniquely receptive to interactions with HEWs or to behavioral change given the particular moment they are passing through related to pregnancy

- Thank you for your concern, dear reviewer. We believe couples are uniquely receptive to interactions with HEWs. Because HEWs have close contact with community members and are well familiarized with the local contexts and cultural norms, in our perspective, providing health education by someone the community knows and trusts will improve the effectiveness of the intervention.

Question: is any of the content uniquely tailored to the life stage of pregnancy?

- Thank you for your concern, dear reviewer. In the 2nd trimester of pregnancy, contents such as an overview of GBV, VAW, and IPV, consequences and common triggers of IPV during pregnancy, and inequitable gender norms will be provided. Contents such as unhealthy and healthy marital relationships, power, control, and IPV, and effective communication and conflict resolution will be given in the third trimester of the pregnancy. This was incorporated into the study protocol approved by the IRB.

**Responses to Reviewer#1:**

Comment: the authors present ‘the protocol for a randomized controlled trial to evaluate couple-based violence prevention education and its ability to reduce intimate partner violence during pregnancy in Southwest Ethiopia’

-We modified the title according to your comment in the revised manuscript.

Comment: authors provide details on the rationale, setting, design and methods, though there are some aspects that require clarification or additional information

-Thank you for your concern, dear reviewer. We addressed in the revised manuscript.

Comment: in the sample size determination section, it is not clear what was calculated versus what was assumed. The way it is presented, all of the information was set. Authors need to clarify what they assumed and what was actually calculated. Also, a cluster size of 27 for each of 8 clusters in a group would result in 216 couples, not 213 couples, so that should also be clarified.

-We corrected and incorporated in the revised manuscript.

Comment: in line 169: "applying permuted block randomization methods" is too broad. For a protocol paper, authors should provide exactly what was (or will be) used to do the randomization.

-We appreciate your concern, dear reviewer. We corrected and incorporated in the revised manuscript

Comment: Is the "Appointment and follow-up section just for the intervention group? This should be clarified. Also, since the intervention is administered in a group format, the entire group agrees on the date of the next session? (I ask, because line 231 says the "educator and the participant" which suggests individual appointments rather than a group session.

-Thank you for your concern, dear reviewer. An appointment and follow-up section is for the intervention group. We corrected and incorporated this in the revised manuscript. Exactly, the entire group should agree on the date of the next session. We corrected and incorporated in the revised manuscript

Comment: line 236 - will authors also identify which couples attend each session (in addition to the number of couples who attended).

-Yes, we corrected and incorporated in the revised manuscript.

Comment 9: Authors should provide more information about the planned GEE models. How is cluster being accounted for? Will authors use the baseline outcome as a covariate in a model for the 8 month assessment or do they plan to model the two time points for each couple? It looks like some information will be collected separately from the wife and from the husband. How will that be handled? Are there any covariates to be included in the models? Are there any planned sub-group analyses or sensitivity analyses? Authors mention using QIC to select the best-fitted models - which models are being compared (this doesn't need to be the exact models, but authors should specify what aspects of the models they are using QIC to help identify)

Comment: how is cluster being accounted for?

-Thank you for your concern, dear reviewer. A working correlation structure will be specified to account within cluster correlation. The choice of preferred working correlation structure will be based on the correlation structure matrix of the observed data. This was incorporated in the revised manuscript.

Comment: Will authors use the baseline outcome as a covariate in a model for the 8 month assessment or do they plan to model the two time points for each couple?

-We plan to model the two time points for each couple.

Comment: It looks like some information will be collected separately from the wife and from the husband. How will that be handled?

-We appreciate your concern, dear reviewer. Couple code will be used to link data from husband and wife. Separate analysis will be conducted for data from wife and husband.

Question: are there any covariates to be included in the models?

-Yes, potential covariates will be included in the model.

Question: are there any planned sub-group analyses or sensitivity analyses?

- Currently, we do not have a plan for subgroup analysis since the trial will be conducted solely in rural kebeles of the districts.

Sensitivity analysis will be conducted to assess the robustness of the intervention effect by changing the assumptions made in the model, such as the working correlation structure, outliers, missing data, and controlling potential confounders. This was incorporated in the revised manuscript.

Comment: Authors mention using QIC to select the best-fitted models - which models are being compared (this doesn't need to be the exact models, but authors should specify what aspects of the models they are using QIC to help identify).

Thank you for your concern, dear reviewer. The Quasi-likelihood under the Independence Model Criterion (QIC) and the Akaike Information Criterion (AIC) will be used to assess model fitness. The QIC and AIC values will be calculated for each model and compared. A model with lower diagnostic criteria (deviance or -2 x log-likelihood ratio) will be used as the best-fitted model

Minor edits

Comment: lines 31 and 361: "STATA" should be "Stata"

-We corrected and incorporated in the revised manuscript.

Comment: lines 32 and 370: "appreciate" should be "assess"

-We corrected and incorporated in the revised manuscript.

Comment: lines 34 and 372: "continues" should be "continuous"

-We corrected and incorporated in the revised manuscript.

Comment: line 66: "such antepartum" should be "such as antepartum"

-We corrected and incorporated in the revised manuscript.

Comment: lines 70 and 72: authors have not defined SDG or IPV

-We defined in the revised manuscript.

Comment: Authors define CBVPE multiple times (for example, lines 85, 88, 92, 95) - it only needs to be defined once in the body of the manuscript and then can be used throughout the rest of the manuscript).

-We defined in only the first place it appeared.

Comment: line 116: "which is one of the ..." is confusing since the start of the sentence talked about 4 out of the 13 districts...authors should clarify.

-We rephrased in the revised manuscript.

Comment: lines 138-139: "Couples who lived at least six months." (Lived together?, lived in the study area?) - This should be clarified.

-We corrected and incorporated in the revised manuscript.

Comment: line 166 - authors mention an alphabetical list - alphabetical list of what?

-We corrected typological error in the revised manuscript.

Comment: line 173: "trimester before." - Before what? I think a word or a word is missing.

-We corrected.

Comment: lines 178-179 contradicts what is said in lines 164-165

-We corrected.

Comment: lines 184-185: authors describe something that took place in June-July 2023, so they should use past tense not future tense ("were" instead of "will be"). Also, not all pregnant women living with their husbands in the clusters were enrolled, right? They had to meet eligibility criteria and consent.

-We used past tense. Also, we revised the inclusion criteria and incorporated in the revised manuscript.

Comment: there are numerous places in the manuscript where lines are duplicated (for example, lines 192-194, 277-279, 281-282m 284-287). Authors should carefully read the manuscript to ensure information is not repeated.

-We corrected.

Comment: line 211 - authors mention 4 main topics, but it looks like there are 5 topics listed (unless health education and communication is considered one topic).

-There are four main topics. Health education and communication is one topic.

Comment 15: line 235: "rule out the reason for the missing" should be rephrased - I don't think authors are ruling out anything, maybe trying to understand the reason for why a couple missed an appointment.

-We rephrased and incorporated in the revised manuscript.

Comment: lines 238-239: "after two months of the intervention" should be rephrased, since I believe the 8 month assessment is being done after the intervention is complete.

-We have rephrased and incorporated in the revised manuscript.

Comment: line 240-241 - would a disagreement or dispute be due to the intervention?

-Yes, dispute may occur due to misunderstanding of the content of the education or pre-existing relationship issue (pre-existing husband violence against wife).

Comment: line 246: authors are not capturing the magnitude of intimate partner violence - they are just capturing whether or not it happened.

-We corrected.

Comment 19: line 362: "tables, diagrams, and figures" are not descriptive statistics - they are ways in which to display descriptive statistics. Authors should clarify their statement.

Absolutely, we corrected and incorporated in the revised manuscript.
