# Supplementary material for: The protocol for a cluster randomized controlled trial to evaluate couple-based violence prevention education and its ability to reduce intimate partner violence during pregnancy in Southwest Ethiopia
Source: PLoS One. 2024 May 13;19(5):e0303009. doi: 10.1371/journal.pone.0303009 (PMC11090299; doi:10.1371/journal.pone.0303009)
Supplement: Attachment — Submitted filename: Rebutal letter.docx [file pone.0303009.s008.docx]

Rebuttal Letter

Dear editor/s

Subject: submission of revised manuscript ID PONE-D-23-32195R1

We hope this email finds you well. We would like to thank all editorial team and reviewer for their investing time and efforts made to provide the valuable feedback on revised manuscript. All the comments raised by editor/s are really important to further improve our manuscript. We have carefully addressed all the comments raised by editorial team point by point. We highlighted with a yellow color in the revised manuscript with track changes.

**Responses to editor(s)**

Journal Requirements: Please review your reference list to ensure that it is complete and correct. If you have cited papers that have been retracted, please include the rationale for doing so in the manuscript text, or remove these references and replace them with relevant current references. Any changes to the reference list should be mentioned in the rebuttal letter that accompanies your revised manuscript. If you need to cite a retracted article, indicate the article’s retracted status in the References list and also include a citation and full reference for the retraction notice.

Response: We have carefully reviewed the reference list so that it is complete and correct.

Editorial comments:

1) Per PLOS One guidelines - please use three heading levels that are all the same size font and bolded.

Response: We have corrected as all level 1 headings are font size of 18, all level 2 headings are font size of 16 and all level 3 headings are font size of 14 and all headings are bolded.

2) When stating author's names in the text, please use the last name only. For example,
a) Pg 5 of the revised manuscript should be Leight et al. (not Jessica)
b) Pg 11 of the revised manuscript should be Nega et al.
c) Pg 12 of the revised manuscript should be Dunkle et al.
d) Pg 12 of the revised manuscript should be Sharma et al.
e) Pg 18 of the revised manuscript should be Peng et al.

Response: corrected
